# Interconnected nanoconfining pore networks enhance catalyst CO_2_ interaction in electrified reactive capture

**DOI:** 10.1038/s41467-025-61407-8

**Published:** 2025-07-04

**Authors:** Hengzhou Liu, Lun An, Peiyao Wang, Christine Yu, Jie Zhang, Heejong Shin, Bosi Peng, Jiantao Li, Matthew Li, Hongmin An, Jiaqi Yu, Yuanjun Chen, Peiying Wang, Kug-Seung Lee, Kanika Lalit, Zeyan Liu, Omar K. Farha, Wenyu Huang, Jefferson Zhe Liu, Long Qi, Ke Xie, Edward H. Sargent

**Affiliations:** 1 Department of Chemistry, Northwestern University, Evanston, IL USA; 2 U.S. DOE Ames National Laboratory, Iowa State University, Ames, IA USA; 3 Department of Chemical Engineering, The University of Melbourne, Parkville, VIC Australia; 4 Chemical Sciences and Engineering Division, Argonne National Laboratory, Lemont, IL USA; 5 Pohang Accelerator Laboratory (PAL), Pohang University of Science and Technology (POSTECH), Pohang, Republic of Korea; 6 Department of Chemistry, Iowa State University, Ames, IA USA; 7 Department of Electrical and Computer Engineering, Northwestern University, Evanston, IL USA

**Keywords:** Electrocatalysis, Chemical engineering, Chemical engineering, Catalyst synthesis, Electrocatalysis

## Abstract

Systems that sequentially capture and upgrade CO_2_ from air to fuels/fuel-intermediates, such as syngas and ethylene, rely on an energy-intensive CO_2_ release process. Electrified reactive capture systems transform CO_2_ obtained directly from carbonate capture liquid into products. Previous reactive capture systems show a decline in Faradaic efficiencies (FE) at current densities above 200 mA/cm^2^. Here we show the chemical origins of this problem, finding that prior electrocatalyst designs failed to arrest, activate, and reduce in situ-generated CO_2_ (*i*-CO_2_) before it traversed the catalyst layer and entered the tailgas stream. We develop a templated synthesis to define pore structures and the sites of Ni single atoms, and find that carbon-nitrogen-based nanopores are effective in accumulating *i*-CO_2_ via short-range, non-electrostatic interactions between CO_2_ molecules and the nanochannel walls. These interactions confine and enrich *i*-CO_2_ within the pores, enhancing its binding and activation. We report as a result carbonate electrolysis at 300 mA/cm^2^ with FE to CO of 50% ± 3%, and with <1% CO_2_ in the tailgas outlet stream. This corresponds to a projected energy efficiency (EE) to 2:1 syngas of 46% at 300 mA/cm^2^ when H_2_ is added using a water electrolyzer.

## Abstract (web summary)

Electrified reactive capture converts CO_2_ from carbonate solutions but suffers from low Faradaic efficiency. Here, the authors report a templated synthesis with defined pores and Ni single atoms to accumulate CO_2_, achieving 46% energy efficiency to syngas at 300 mA/cm^2^

## Introduction

**Fig. 1:**
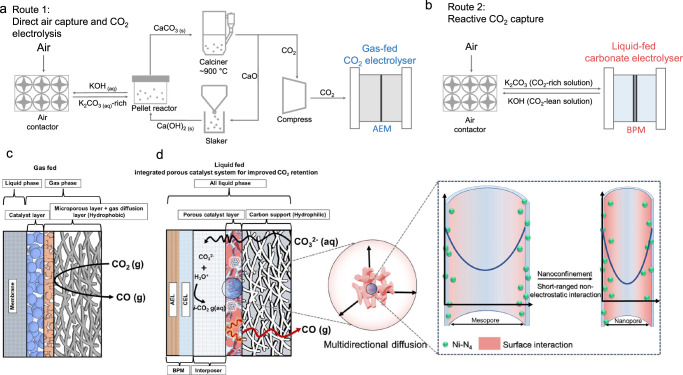
Schematic illustration of systems. **a** Sequential process of direct air capture (DAC) coupled with CO_2_ electrolysis (Route 1) vs. **b** integrated process of reactive CO_2_ capture (Route 2). **c**, **d** Comparison of CO_2_ transport and conversion in two systems: **c** A gas-fed CO_2_ electrolysis system incorporating a hydrophobic microporous layer, a gas diffusion layer, and a catalyst layer. The CO_2_ reduction reaction took place at the catalyst surface within a three-phase boundary. **d** The current carbonate-based reactive capture system operates in a hydrophilic environment. In this work, we integrate a porous catalyst supported on carbon, featuring multidirectional nanoporous diffusion channels. These structures promote short-range, non-electrostatic interactions between pore walls and reactants, enhancing the retention and accumulation of *i*-CO_2_ and thereby maximizing its utilization. Source data for the results are provided as a Source Data file.

Carbon capture and utilization (CCU)—such as direct air capture (DAC) followed by electrochemical upgrade to fuels/fuel precursors—offers, if energy-efficient and powered using low-carbon electricity, to lower the carbon intensity of fuels and chemicals (Fig. 1a)^1–5^. For the DAC stage, an alkaline solution such as KOH captures CO_2_ as K_2_CO_3_^5^, and concentrated CO_2_ is released through energy-intensive drying and calcination steps at ~900 °C, requiring 8–10 GJ/tonne CO_2_. Considering the case of upgrade of this CO_2_ (44 g/mol) to CO (28 g/mol), this adds a further energy consumption of (44/28) × (8-10 GJ/tonneCO_2_) = 13–16 GJ/tonneCO. This value, which is additive atop the CO_2_-to-CO electrolyzer energy, is appreciable, itself residing well above the LHV of the final intended product.

Reactive capture, by contrast, integrates CO_2_ capture and conversion in a single system (Fig. 1b)^6–10^. In comparison with the low CO_2_ concentration in air (~400 ppm), a post-capture liquid consisting of carbonate-rich solution^5,11^ provides a more concentrated carbon-containing feedstock. Both bipolar membrane (BPM) and cation exchange membrane (CEM)-based electrolyzers have been advanced in carbonate-liquid-fed system^12–15^.

In conventional, gas-fed, CO_2_ reduction systems (Fig. 1c), a hydrophobic support is employed, and CO_2_ gas-phase transport occurs through the gas diffusion layer and a microporous layer, providing an abundance of the needed feedstock to the catalyst surface^16–19^.

In contrast, carbonate-liquid-fed systems (Fig. 1d and Supplementary Fig. 1) are flooded, and CO_2(g(aq))_, i.e., gaseous CO_2_ dissolved in aqueous solution, is generated only once CO_3_^2−^(aq) reaches the cation exchange layer (CEL) side of the BPM and is acidified via H_3_O^+^ provided by water dissociation in the BPM. The CO_2(g(aq))_ must then travel back to the catalyst, moving first through a ~ 100 µm thick porous interposer (its purpose to establish a pH difference between CEL and catalyst: acidic at the CEL edge for CO_2_ regeneration, near-alkaline at the catalyst to avoid undue HER)^14^. The rate of provisioning of this *i*-CO_2_ is limiting in reactive capture systems (Supplementary Note 1): at current density 100 mA/cm^2^, the best-case *i*-CO_2_ supply rate is ~0.7 mL min^–1^ cm^–2^; the value is lower during electrolysis, during which OH^-^ generation leads to an even higher local pH that further consumed *i*-CO_2_. Thus, successful reactive capture systems must be maximally selective and active for CO_2_ reduction, even in an *i*-CO_2_-starved environment^14^.

The cathode catalyst in prior reactive capture systems has typically been a substantially planar catalyst (Supplementary Fig. 2), such as Ag (for CO) and Cu (for C_2+_ products)^12,13,15,20–22^, the layer residing atop the flooded hydrophilic support. We noted that such systems diminish in their performance (e.g., the FE to reduced carbon products declines below 30%) even at moderate current densities such as 100 mA/cm^2^ and above. Similar trends have been seen in reactive capture using Ni single-atom^21^ and molecular^14,23^ catalysts.

## Results

### Nickel single-atom catalysts with engineered pore structure

We hypothesized that, in carbonate-fed reactive capture systems, a porous catalyst (Fig. 1d) could—once optimal conditions of pore size and pore density were found—facilitate CO_2_ retention and accumulation at the 3D solid-phase dispersion of electrocatalytic sites^24^. We therefore adopted an approach in which the porous transport layer itself served as the catalyst. Once *i*-CO_2_ was delivered, it would then locally bind and activate to provide CO_2_ reduction. We posited that engineering of (1) the degree of pore alignment vs. anisotropy (2) the size of pores could maximize the *i*-CO_2_:catalyst interaction, enabling selectivity in favor of CO_2_ electroreduction even as current density increased.

Since Ni single atoms (Ni-SAC) are known-good CO_2_-to-CO catalysts, we sought to form this porous catalyst from Ni, coordinated with nitrogen-carbon matrix, in turn dispersed and coordinated into a conductive carbon support. We airbrushed the resulting catalyst onto a hydrophilic substrate made of carbon paper. Porous Ni single-atom catalysts are prepared through a template-controlled coordination-condensation-carbonization synthesis (Supplementary Fig. 3 and “Methods”)^25^. Ni^2+^ is first coordinated with ethylenediamine and polymerized with carbon tetrachloride within the pores of silica template, after which the template is removed. This synthesis process enables the pre-coordination of Ni with N, improves Ni dispersion, and allows precise control over the morphology and porous structure.

**Fig. 2:**
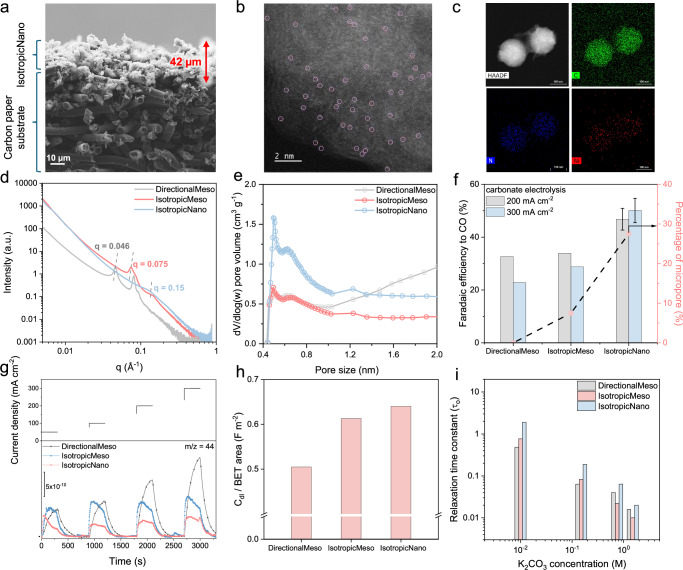
*i-*CO_2_ transport kinetics in pores. **a** Cross-sectional SEM image of the cathode electrode consisting of the IsotropicNano catalyst coated on a hydrophilic carbon paper substrate. **b** HRTEM image and **c** HAADF-STEM-EDS mapping of the IsotropicNano catalyst. **d** Synchrotron-based Small-Angle X-ray Scattering (SAXS) profiles and **e** BET pore size distribution in the 0.4–2 nm range. **f** Faradaic efficiency (FE) to CO and percentage of nanopores as a function of cathode electrode at 200 and 300 mA/cm^2^ in a carbonate electrolyzer with BPM. Where error bars are shown, values are means, and error bars indicate s.d. (*n* = 3 replicates). **g** EC-MS signal of CO_2_ (m/z = 44) measured across a current density range of 50–300 mA/cm^2^. **h** Total capacitance (C_dl_) normalized by the BET surface area. **i** Calculated τ_o_ in dilute K_2_CO_3_ electrolytes. Relaxation time constant (τ_o_) is calculated from the peak frequency (f_o_) of imaginary part of complex capacitance (C_im_) using the relation τ_o_ = (2πf_o_)^–1^. Source data for the results are provided as a Source Data file.

We varied the pore structure of candidate porous catalysts by using a library of templates that modulated both the pore channel topology and the average pore diameter. We explored three templates (Supplementary Fig. 4 and Supplementary Table 1) and also varied the post-synthesis NH_3_ treatment. One class of template, whose corresponding porous catalyst we term DirectionalMeso, is expected to provide substantially 1D transport through the catalyst layer. A second, IsotropicMeso, consists of mesopores (2–50 nm) having no directional preference. A third, IsotropicNano, includes both meso and nano (<2 nm) pores. In each case, we optimized the porous catalysts thickness, finding that a 40 µm (Fig. 2a) thickness maximized electrolysis performance.

Each class of porous catalyst consisted of similar graphitic carbon (XRD and HRTEM, Supplementary Figs. 5–13). XPS (Supplementary Fig. 14) showed in each case N 1 *s* spectra of four types of nitrogen (pyridinic, pyrrolic, graphitic, and oxidized N) in similar ratios. Elemental analysis showed similar nitrogen content (9–10 wt%, Supplementary Table 2). The Ni 2*p* spectra, with binding energies ~854.2 eV, indicated a slightly oxidized state of Ni^26^. STEM-HAADF imaging (Fig. 2b and Supplementary Figs. 6–13) indicated the formation of single-atom Ni, while STEM-energy-dispersive X-ray spectroscopy (STEM-EDS) mapping suggested a uniform distribution of Ni within each porous catalyst (Fig. 2c).

Synchrotron-based Small-Angle X-ray Scattering (SAXS) indicated periodic and symmetric pore arrangements^27–29^, as shown by correlation peaks in the profiles (Supplementary Note 2 and Fig. 15). Fractal analysis^29^ showed mass fractal structures (fractal dimensions: D = 1–3), where the mass of the porous network scales non-linearly with size, consistent with self-similar structures formed via templating. Higher D values in IsotropicNano suggest increased spatial complexity, indicating greater surface roughness and a more disordered pore structure. The primary SAXS peaks (Fig. 2d), corresponding to different Miller indices, reflect average pore-to-pore distances that decrease from ~15 nm in DirectionalMeso to ~3 nm in IsotropicNano. It indicated IsotropicNano possesses the smallest average pore diameter, consistent with its high nanopore fraction and the cubic lattice arrangement of its pore network (Supplementary Note 2).

N_2_ adsorption-desorption isotherms further showed type-IV hysteresis loops indicative of a mesoporous structure (Supplementary Figs. 16–20 and Table S3). Brunauer–Emmett–Teller (BET) surface area and average pore size analysis indicated a predominance of mesopores in DirectionalMeso and IsotropicMeso, whereas IsotropicNano exhibited the expected higher proportion of nanopores (Supplementary Figs. 16–20 and Fig. 2e). SEM, TEM, and HAADF-STEM imaging revealed distinctive pore morphologies (Supplementary Figs. 6–13, 21–26): DirectionalMeso displayed a cylindrical structure with unidirectional channels and a linear pore array, whereas IsotropicMeso and IsotropicNano exhibited nanosheet and nanosphere structures.

### Electrolysis performance as a function of pore structure

We integrated the catalyst layers into a BPM-based system fed with carbonate solution. By optimizing the Ni precursor amount, calcination temperature, and adjusting the pore channel length within the 0.35–0.6 μm range, we achieved a peak FE_CO_ of 40% at 100 mA/cm2 (Supplementary Fig. 27) on DirectionalMeso. However, this FE_CO_ rapidly declined to below 30% at 200–300 mA/cm^2^ (Fig. 2f).

In the IsotropicMeso catalyst, i.e., having no directional preference and a pore diameter ~ 2–50 nm, we observed a higher FE_CO_ in the higher current density range of 200–300 mA/cm^2^, though FE_CO_ remained below 35% (Fig. 2f). It was only when we combined multidirectional diffusion with the addition of <2 nm nanopores in IsotropicNano that FE_CO_ rose to 50% ± 3%, accompanied by the lowest extent of the OH⁻–CO_2_ neutralization side reaction and the lowest CO_2_ gas evolution at the cathode outlet (Supplementary Table 4).

Inductively coupled plasma mass spectrometry (ICP-MS) analysis (Supplementary Table 5) indicates that the increased catalyst activity in IsotropicNano was not a result of higher Ni content, for IsotropicNano exhibited the lowest Ni mass loading (1.35 wt%) among all samples. The normalized Turnover Frequency (TOF) for CO generation in IsotropicNano was 1.9 and 3.3 times higher than that of DirectionalMeso and IsotropicMeso, respectively, at 300 mA/cm^2^. Further increasing the Ni content in IsotropicNano did not enhance performance: it leads to nanoparticle formation, favoring the hydrogen evolution reaction (HER) (Supplementary Figs. 28–29).

Control experiments showed that, unlike hydrophobic gas-fed CO_2_RR systems or non-porous Ag-based catalysts—where only the surface layer actively participates in the reaction—an optimal IsotropicNano loading of 1.5–2 mg/cm^2^ is essential in the fully-flooded carbonate system to maximize Ni single-site utilization throughout the entire catalyst layer (Supplementary Note 3 and Figs. 30–32).

We further employed in situ electrochemical mass spectrometry (EC-MS) to detect the unreacted *i*-CO_2_(g) at the cathode outlet (Fig. 2g). IsotropicNano showed minimized CO_2_(g) present in the tailgas, especially at high current densities. In contrast, the one-dimensional/directional diffusion in DirectionalMeso may provide too-efficient egress of *i*-CO_2_ from the entrance of the porous catalyst layer (facing the interposer and the CEM side of the BPM) to its plane of egress (atop the GDL), preventing selectivity in *i*-CO2RR from being retained at high currents. The 3D interconnected pore network in IsotropicNano—particularly with its higher fraction of nanopores—enhanced *i*-CO_2_ retention and promoted its efficient utilization within the catalyst layer.

These results highlight that the entire catalyst layer plays a crucial role in facilitating *i*-CO_2_ transport, retention, and penetration into the interconnected pores, thereby maximizing catalytic efficiency.

### Characterization of *i*-CO_2_ transport kinetics in pores

We employed electrochemical impedance spectroscopy (EIS) and calculated the electric double-layer capacitance (C_dl_), which represents the electrochemically-accessible surface area (electrolyte-wetted) for the reaction (Supplementary Fig. 33)^30–32^. The BET area provides the physical surface area of the catalyst. Reporting C_dl_ normalized to BET, C_dl_/BET (Fig. 2h), reflects the fraction of the catalyst’s physical surface area that actively participates in the electrochemical reaction, indicating active site utilization. In a 1.5 M K_2_CO_3_ electrolyte, the total capacitance of IsotropicNano (580 F g⁻¹) exceeds that of the other two catalysts (430 F g⁻¹ for DirectionalMeso and 490 F g⁻¹ for IsotropicMeso). IsotropicNano achieved the highest C_dl_/BET of 0.64.

We then examined CO_2_ diffusion and affinity under dry, wetted, and wetted with applied bias conditions. Under dry conditions, CO_2_ affinity was assessed using CO_2_ adsorption isotherm measurements. IsotropicNano showed increased CO_2_ uptake over a wide pressure range (Supplementary Fig. 34). The measured CO_2_ adsorption heat for IsotropicNano ranged from 24 to 36 kJ mol⁻¹, consistently higher than that of DirectionalMeso and IsotropicMeso.

Under wet conditions, we evaluated reactant retention within the pores, expressed by the relaxation time constant (τ_o_) (Fig. 2i) measured via EIS. τ_o_ reflects the rate of reactant transport in the pores, reporting thus on resistance to rapid penetration, and is sensitive to both the pore size and the pore tortuosity^32^. Figure 2i shows a trend in τ_o_: IsotropicNano (20 ms) > IsotropicMeso (16 ms) > DirectionalMeso (10 ms). In more dilute electrolytes, the τ₀ differences among the catalysts increased further (Fig. 2i and supplementary Fig. 35).

We employed gas-fed assessed CO_2_ electrolysis systems to study CO_2_ transport and retention further (Supplementary Fig. 36). In three-compartment flow cells, IsotropicNano maintained a high FE of > 90% even as the CO_2_ flow rate was turned down to 2.5 mL min^−1^ cm^−2^ and the CO_2_ partial pressure was reduced to 20%. In a membrane electrode assembly (MEA)-based CO_2_ electrolyzer with an anion exchange membrane (AEM), IsotropicNano achieved an impressive single-pass (SP) efficiency, the ratio of CO outlet flow to total CO_2_ input flow, of 45% at 300 mA/cm^2^ (Supplementary Fig. 37).

These experimental results indicate that the pore structure of IsotropicNano enhances the interaction of CO_2_ with catalyst active sites, extending *i*-CO_2_ retention within pores. This enables a greater accumulation of *i*-CO_2_ within nanoconfined spaces.

### Simulation of *i*-CO_2_ transport kinetics in pores

Our electrochemical reactive capture systems in confined pores involve tightly coupled processes—transport, adsorption, solvation, charge distribution, and reactions—spanning multiple scales^33^. These effects are strongly influenced by local pore environments and remain challenging to fully capture with current models, especially under nanoconfinement where continuum assumptions break down.

CO_2_ adsorption and mobility within nanoporous environments influence reactant availability at electrochemical interfaces^33^. The physisorption of CO_2_, in particular, plays a central role by establishing a local reservoir of molecules that are readily available to participate in subsequent, slower steps such as chemisorption and electron transfer^34,35^. Understanding how pore geometry and surface characteristics influence CO_2_ adsorption and diffusion is of use for optimizing catalyst design and performance. In this context, our simulation work focuses specifically on the physisorption and transport behavior of CO₂ in sub-3 nm pores by combining density function theory (DFT) and molecular dynamics (MD) simulations.

**Fig. 3:**
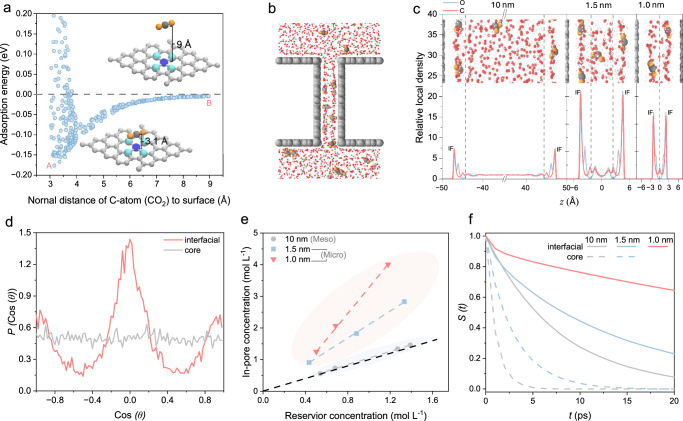
Simulations of *i*-CO_2_ transport kinetics in pores. **a** CO_2_ adsorption energy as a function of distance normal to the Ni single atom surface, calculated using density functional theory (DFT). A total of 400 cases was considered, accounting for CO_2_’ random rotations and distributions in the parallel plane at each specified normal distance. **b** Schematic showing the slit-based model employed in molecular dynamics (MD) simulations to approximate pore-size effects. **c** Local relative density distribution in the normal direction to the pore surface, comparing three pore sizes: 1.0 nm, 1.5 nm, and 10 nm. **d** The orientation analysis of CO_2_ within the 1.0 nm slit. The angle $\theta$ is defined as the direction of C–O bond relative to the normal plane of the catalyst surface. **e** Summary of CO_2_ in-pore concentration as a function of CO_2_ reservoir concentration, comparing different pore sizes: 10 nm, 1.5 nm, and 1.0 nm. **f** The survival time correlation function (S(τ)) as a function of time interval (τ) within the interfacial and center regions of a nanoslit. Source data for the results are provided as a Source Data file.

We then performed DFT calculations to evaluate the CO_2_ adsorption energy as a function of the distance normal to the catalyst surface (Fig. 3a). When the surface is uncharged, as the distance decreases from 9 Å to 3 Å, the CO_2_ adsorption energy changes from zero (non-adsorption) to negative values (adsorption) and eventually to positive values (repulsion). We observed a maximum adsorption energy of −0.175 eV at a distance of 3.1 Å, where the O-C-O angle remains at 180° and aligns above the Ni atom (inset of Fig. 3a), indicating a physisorption interaction between CO_2_ and uncharged Ni. Removing Ni atoms, i.e., changing to pure graphene, results in a lower CO_2_ adsorption energy (i.e., 0.160 eV, Supplementary Fig. 38), indicating that the atomically dispersed Ni enhances CO_2_ physisorption. When the Ni single atom surface becomes charged, recent work shows that the CO_2_ adsorption would be further enhanced, where CO_2_ is chemisorbed at the surface^36^. The H_2_O adsorption energy on Ni single atom surface is less than half that of CO, suggesting a means by which the rate of HER is diminished (Supplementary Fig. 39).

We employed MD to investigate the CO_2_ distribution in a nanoconfined pore environment filled with water molecules. The MD model employs a nanoslit in contact with bulk reservoirs on both sides (Fig. 3b). We considered three slit sizes, 1.0 nm, 1.5 nm, and 10 nm, and varied CO_2_ concentration in the bulk reservoir to represent dynamic CO_2_ concentration in reactive capture systems operating at different current densities. We refined the force field parameters in MD simulations using a genetic algorithm (see Methods for details), training these to match DFT results.

The results show that reducing the slit size to sub-2 nm enhances CO_2_ participation within the pore. When the slit surface is uncharged, and the slit size is 10 nm, the in-pore CO_2_ concentration (red line) is equal to the bulk reservoir concentration (black line). As the slit size decreases to below 2.0 nm, the in-pore concentration surpasses the bulk concentration by 4x (Fig. 3e). This enhanced participation is a result of enriched CO_2_ adsorption in the near-surface region, i.e., two sharp CO_2_ density peaks near the surface in Fig. 3c, consistent with the DFT results (Fig. 3a). As slit size decreases, the pore volume occupied by these CO_2_ layers increases (Supplementary Fig. 40), raising the overall CO_2_ in-pore concentration. Our results show that the nanoconfinement effect arises from short-range non-electrostatic interactions between the pore surface and CO_2_ molecules, leading to increased reorientation and rearrangement of CO_2_ within the pore (Fig. 3d and Supplementary Fig. 41). MD indicates that H_2_O does not interfere with CO_2_ adsorption at the catalyst surface (Supplementary Fig. 42), consistent with its weaker surface adsorption energy.

The mobility/transport kinetics of CO_2_ within confined pores were also investigated in MD simulations using the survival time correlation function, S(τ), which denotes the likelihood that CO_2_ remains within a specified region over a time interval τ (Methods). We analyzed S(τ) for CO_2_ in the interfacial (within 5.0 Å distance from the surface) and central regions of the nanoslit. In Fig. 3f, the interfacial region $S(\tau )$ decays much more slowly than the central region, indicating a significantly lower CO_2_ mobility at the interface. In addition, the smaller pores have a higher interfacial $S(\tau )$ value, indicating that confinement further suppresses the transport dynamics of CO_2_.

Our results offer insights into sub-nanometer variations in CO₂ adsorption density, interfacial residence times, and transport kinetics—key factors in determining reactant availability at active sites. They are complementary to conventional continuum models, such as Poisson–Nernst–Planck or volume-averaged frameworks^37^, which can couple ion transport and reaction kinetics but typically assume spatial uniformity and size-independent properties—assumptions that break down under nanoconfinement^38^. We hope these insights will inspire the development of future multiscale reaction–transport models—a task that remains both computationally demanding and methodologically underdeveloped.

### Nanoconfined environments for enhanced CO_2_ binding and activation

To investigate how *i*-CO_2_ transport within pores influences CO_2_ reduction kinetics in a reactive capture system, we tested the porous catalysts in a simulated low-concentration CO_2_ electrolysis setup using K_2_CO_3_ as the electrolyte. A Michaelis-Menten-based kinetic model^39,40^ was used to describe the CO_2_-to-CO pathway on Ni-SAC catalysts, where CO_2_ binds to Ni sites forming an intermediate (Ni– CO_2_∙−) that releases CO (Supplementary Note 5 and Table S8).

**Fig. 4:**
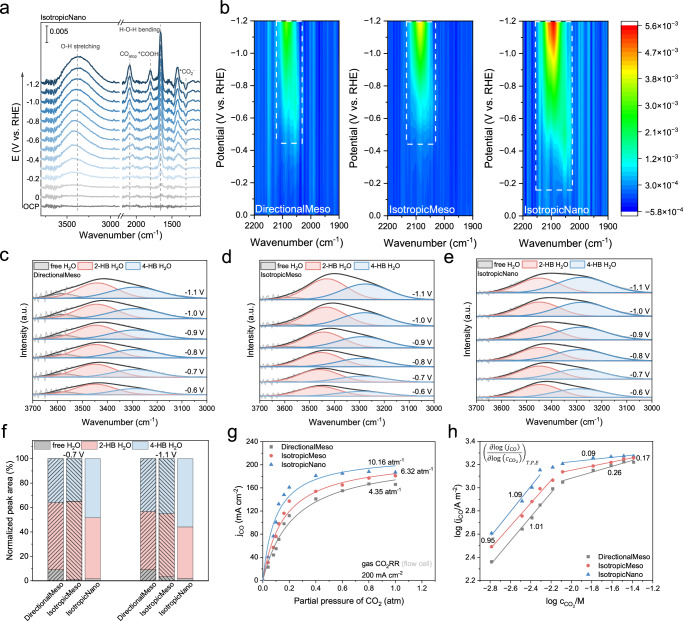
Mechanistic study of confinement effects in nanopores. **a** In situ ATR-SEIRS spectra recorded on IsotropicNano catalyst at OCP and under applied potentials ranging from 0 to −1.2 V versus RHE. 20% CO_2_ gas (balanced by Ar) was purged into the system to form a CO_2_-satured solution. **b** Contour map of in situ ATR-SEIRS spectra on three catalysts: DirectionalMeso, IsotropicMeso, and IsotropicNano, at the wavenumber range of 1900 to 2200 cm^−1^. **c**–**e** Potential (versus RHE)-dependent fitted bands of interfacial water (3000–3600 cm^−1^) on DirectionalMeso, IsotropicMeso, and IsotropicNano, respectively. **f** Variation in the proportion of interfacial water as a function of potential (versus RHE) for the three catalysts. **g** partial current density of CO (j_CO_) on three catalysts as a function of the CO_2_ partial pressure (*P*_CO2_) at 200 mA/cm^2^. The partial pressure of CO_2_ was adjusted by mixing CO_2_ with Ar gas. The binding affinity constants of the catalysts are described in the inset by fitting *j*_CO_ − *P*_CO2_ plots. **h** Reaction order fitting of log(*j*_CO_) versus log(*c*_CO2_), showing two distinct regimes: diffusion-limited and kinetically controlled. Source data for the results are provided as a Source Data file.

We derived a binding constant (Fig. 4g), $K=\frac{{k}_{{{\rm{a}}}}}{{k}_{{{\rm{d}}}}+{k}_{{{\rm{cat}}}}}$, to represent the catalyst’s CO_2_ binding affinity, where *k*_a_, *k*_d_, and *k*_cat_ are the rate constants for adsorption, desorption, and catalysis, respectively. By varying the CO_2_ concentration from 5% to 100% and performing electrolysis at 200 mA/cm^2^, we fitted the K values: IsotropicNano (10.2) > IsotropicMeso (6.3) > DirectionalMeso (4.4), indicating favorable CO_2_ binding on IsotropicNano.

Reaction order analysis revealed two regimes (Fig. 4h): a diffusion-limited region where all samples exhibited a reaction order ~1, and a kinetically controlled region. IsotropicNano showed a narrower diffusion-limited range (0–12% CO_2_) than the others (0–20%), indicating more efficient CO_2_ utilization under lean conditions. In the kinetically controlled regime, DirectionalMeso and IsotropicMeso displayed higher reaction orders (0.2–0.3), suggesting continued dependence on CO_2_ concentration due to mass transport limitations. Temperature-dependent studies further showed that IsotropicNano had the lowest activation energy, indicating superior charge transfer properties (Supplementary Fig. 44).

To gain molecular-level information on CO_2_-to-CO conversion, we conducted in situ attenuated total reflectance–surface-enhanced infrared absorption spectroscopy (ATR-SEIRAS) under simulated low-concentration CO_2_ conditions. Controlled potential experiments revealed characteristic peaks for CO_2_ reduction intermediates (*COOH, *CO) and interfacial water across all three samples (Fig. 4a and Supplementary Fig. 45).

The linearly adsorbed *CO (CO_atop_) peak (2000–2100 cm⁻¹)^41,42^ increased in intensity at more negative potentials (Fig. 4b). IsotropicNano showed ~200 mV lower overpotential for *CO appearance compared to DirectionalMeso and IsotropicMeso, indicating enhanced *COOH stabilization or earlier *CO formation. From −0.2 to −1.2 V (*vs*. RHE), IsotropicNano exhibited a blue shift in *CO stretching (2049 → 2100 cm⁻¹), suggesting weakened CO binding—likely due to reduced back-donation from metal d-orbitals to CO’s π orbital, which favors *CO desorption and reduces surface poisoning^43^. A Stark tuning rate of ~51 cm⁻¹/V, observed on IsotropicNano, highlights its sensitivity to interfacial electric fields and its facilitation of *CO desorption^41^. In contrast, *CO peaks on DirectionalMeso and IsotropicMeso exhibited minimal Stark tuning or remained unchanged, indicating stronger binding and limited desorption capability.

We also monitored the O–H stretching region (~3000–3600 cm⁻¹) to probe hydrogen bonding networks and interfacial water structure (Fig. 4a and Supplementary Fig. 45). All samples showed a red shift of peaks at more negative potentials, indicating increased hydrogen bonding and enhanced water structuring^44^. Such structured H-bond networks can stabilize key intermediates (*COOH or CO_2_^⁻^) through polar interactions, lower the activation barrier for CO_2_ reduction, and help sustain a local proton source without promoting excessive HER^45^.

Deconvolution revealed peaks at 3600, 3450, and 3270 cm⁻¹, corresponding to free water, weakly hydrogen-bonded (2-HB), and strongly hydrogen-bonded (4-HB) species, respectively (Fig. 4c–f and Supplementary Table 9)^46,47^. Free water decreased significantly at high overpotentials on all samples. Notably, IsotropicNano showed a higher 4-HB/2-HB ratio and stronger spectral shifts, suggesting more robust H-bond networks that facilitate proton-coupled electron transfer (PCET) and intermediate stabilization during CO_2_ reduction.

Taken together, the electrokinetic and in situ spectroscopic data indicate that IsotropicNano’s nanoconfined 3D pore network enhances CO_2_ binding affinity, stabilizes key intermediates, facilitates CO desorption, and promotes proton activation—collectively improving CO production. These tailored local reaction environments are resulted from the nanoconfined spaces that concentrated local reactants, which enhanced local electric field, tailored double layer structure, and the layout or binding angles of CO_2_ and H_2_O^35,48^.

### Optimizing reactive capture system to increase energy efficiency

In the carbonate-liquid-fed reactive capture system, we employed IsotropicNano as the cathode catalyst in a BPM electrolyzer (Supplementary Fig. 46). A hydrophilic interposer layer was introduced between the CEL and catalyst layer to establish a pH gradient, ensuring a low pH at the CEL for efficient *i*-CO_2_ generation and a higher pH at the catalyst layer to facilitate CO_2_RR^14^. We optimized the interposer thickness, pore size, and material type, identifying the hydrophilic mixed cellulose ester (MCE) with a 135 µm thickness as achieving the highest FE_CO_ (Supplementary Fig. 47).

**Fig. 5:**
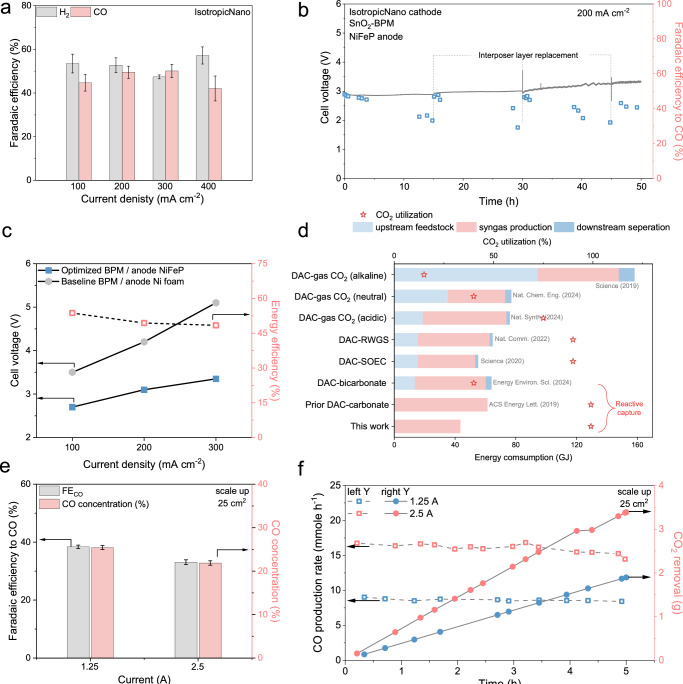
Optimizing the reactive capture system for enhanced energy efficiency and scalability. **a** Faradaic efficiency to CO and H_2_ for IsotropicNano catalysts at 100–400 mA/cm^2^. Where error bars are shown, values are means, and error bars indicate s.d. (*n* = 3 replicates). **b** Cell voltage (left Y-axis) and energy efficiency (right Y-axis) for carbonate electrolysis (1.5 M K_2_CO_3_) at different cell configurations. The energy efficiency is calculated from experimental data for a system that employed an optimized BPM and a NiFeP anode. **c** Cell voltage (left Y-axis) and Faradaic efficiency to CO (right Y-axis) for 50 h of electrolysis at 200 mA/cm^2^ with an optimum cell configuration: IsotropicNano cathode, SnO_2_-BPM, and NiFeP anode. **d** Comparison of energy consumption for the generation of 1 tonne syngas for different processes. In order, top to bottom, these are: direct air capture (DAC) coupled with gas CO_2_ electrolysis in alkaline^59^, neutral^60^, and acidic^61^ conditions; DAC-coupled with reverse water gas shift (RWGS)^62^; DAC coupled with a solid oxide electrolysis cell (SOEC)^63^; bicarbonate electrolysis best prior performance^21^; carbonate best prior performance^12^; and carbonate electrolysis reported in this work. **e** Faradaic efficiency to CO (left Y-axis) and CO concentration (right Y-axis) at the cathode outlet in the scaled electrolyzer. In the scaled flow plates, the channel depth is 1 mm, width is 1 mm, and the rid distance is 3.5 mm. The active area length is 50 mm, resulting in an electrode active area of 25 cm^2^. Where error bars are shown, values are means, and error bars indicate s.d. (*n* = 3 replicates). **f** CO production rate (mmol h-1, left Y-axis) and CO_2_ removal amount (right Y-axis) during 5 h of electrolysis at currents of 1.25 A and 2.5 A. Source data for the results are provided as a Source Data file.

In terms of DAC capture kinetics (using concentrated KOH) and subsequent reactive capture product selectivity, we selected 1.5 M K_2_CO_3_ as the feed solution (Supplementary Fig. 48). We observed that FE_CO_ is maintained >40% at 100–400 mA/cm^2^ (Fig. 5a), with a peak FE_CO_ of 50% ± 3% achieved at 300 mA/cm^2^. The FE_CO_ thus appreciably outperforms the best-prior FE_CO_ of 20% at 300 mA/cm^2^ in BPM-based carbonate electrolyzers^12^.

The energy efficiency (EE) depends also on the full cell voltage. The baseline case, with its FE_CO_ = 30% and V_cell_ = 3.4 V at 100 mA/cm^2^, corresponds to an energy cost of 64 GJ/tonne of 2:1 syngas. This projected energy consumption assumes the use of an 80%-efficient a solid oxide electrolyzer cell (SOEC) to balance CO to the final syngas composition of 2:1 H_2_: CO.

We aimed therefore to pursue higher EE by lowering voltage. To study the origins of high voltage, we employed a diagnostic electrolyzer, focusing on the 300 mA/cm^2^ case which required V_cell_ = 5.1 V (Fig. 5c and Supplementary Fig. 49). The BPM accounted for 53% of the total V_cell_ (Supplementary Fig. 49). When we replaced the BPM with one employing a nanoparticle SnO_2_ WD catalyst^49^ and a NiFeP anode, V_cell_ declined to 3.3 V at 300 mA/cm^2^. Uniting the best catalyst with the best BPM and using a 65%-efficient water electrolyzer to supply the missing H_2_ to syngas composition of 2:1 H_2_: CO, we obtained a syngas EE of 46% at 300 mA/cm^2^ (Fig. 5c), surpassing the previous best EE of 28% at the same current density^12^.

To examine the operating stability of the full electrolyzer with IsotropicNano cathode, SnO_2_-BPM, and NiFeP anode, we characterized the system at 200 mA/cm^2^ over the course of 50 h of electrolysis (Fig. 5b). The system retained FE > 40% and V_cell_ of ~3.0 V, but only if we replaced the hydrophilic MCE interposer layer after 15 h.

X-ray absorption near-edge structure (XANES) spectra showed similar Ni K edges before and after electrolysis (Supplementary Fig. 50a). X-ray absorption fine structure spectrometry (EXAFS) showed the local coordination environment of Ni atoms, with a dominant peak at around 1.4 Å, assignable to the Ni-N coordination (Supplementary Fig. 50b), indicating a stable Ni single-atom structure. This Ni-N coordination anchors Ni atoms within the carbon-nitrogen framework, preventing the aggregation and migration of Ni under biased conditions. Anchoring Ni within the porous matrix minimized the formation of Ni particles that might, if formed, block the nanopore entrance.

The combination of FE_CO_, V_cell_, EE, and stability is comparable to, or exceeds, those reported in previous reactive capture studies utilizing various capture media or targeting different products (Supplementary Table 10).

It is possible (and undesired) for *i*-CO_2_ to diffuse all the way through the catalyst layer and emerge as CO_2_ in the tailgas stream. To keep track of the efficiency with which CO_2_ is instead desirably converted or utilized, we define:

1 $${{\rm{Carbon\; utilization}}}=\left(1-\frac{{n}_{{CO}2\left(g\right)}}{{n}_{{CO}2}^{0}}\right)\times 100\,\%$$

where ${n}_{{CO}2}^{0}$ is the number of moles of CO_2_ generated by the action of the BPM; and ${n}_{{CO}2\left(g\right)}$ is the number of moles of gas CO_2_ detected at the outlet of the catholyte. We observed carbon utilization >99% at 300 mA/cm^2^, with CO_2_ concentration in the cathode outlet <1%. The outlet CO concentration reached ~50% also at 300 mA/cm^2^. This combination of carbon utilization and CO concentration is the highest—looking across both reactive capture and gas-fed CO_2_ reduction systems (acidic, neutral, and basic conditions) –reported to date (Supplementary Table 11).

We conducted an energy analysis of systems of interest (Fig. 5d and Supplementary Note 6): i) sequential capture-and-release followed by gas-fed electrochemical CO_2_ upgrade; ii) sequential capture-and-release followed by reverse water-gas shift (RWGS) using hydrogen from an efficient water electrolyzer; iii) the integrated reactive capture approach explored in this study and prior studies, with H_2_ is supplemented from a water electrolyzer to obtain H_2_:CO of 2:1 syngas. The integrated reactive capture process in this work offers the lowest projected energy expense in GJ/tonne of syngas (43 GJ/tonne), its advantage deriving the avoidance of the CO_2_ regeneration and circulation steps in sequential DAC-plus-electrolysis.

We further identified key differences between carbonate- and previously reported bicarbonate-based reactive capture systems^21,50^ in terms of electrolysis environment, energy consumption, and capital cost (Supplementary Note 7)^10^. Bicarbonate-liquid-fed electrolyzers typically show high FE_CO_ due to greater *i*-CO_2_ availability and a near-neutral buffered environment that suppresses *i*-CO_2_ consumption through local acid-base reactions; however, they exhibit lower *i*-CO_2_ utilization compared to carbonate systems (40–60% vs. >99%)^21,50^. While flue gas capture (4–15% CO_2_) via K_2_CO_3_ (aq) absorbent can produce a bicarbonate-rich solution, it is kinetically unfavorable for DAC due to the low CO_2_ concentration (~400 ppm)^10,11^. Converting post-captured carbonate to bicarbonate for continuous electrolysis adds extra processing steps, increasing both capital and operating costs^11^. Therefore, improving selectivity and lowering cell voltage in carbonate-based systems is still critical for efficient air-to-product CCU.

Finally, we carried out an initial scaling study, employing a 25 cm^2^ BPM at 25 cm^2^ catalyst-on-support (Supplementary Fig. 51). At currents of 1.25 A (50 mA/cm^2^) and 2.5 A (100 mA/cm^2^), FE_CO_ was 38% and 33% (Fig. 5e). The CO_2_ concentration in the cathode outlet remained <1% (Fig. 5e). We note that a low CO_2_ concentration in the outlet tailgas will minimize costs of CO_2_/CO separation.

Additionally, we recorded the time-averaged rate of CO production, observing 8.6 mmol h^−1^ at 50 mA/cm^2^ and 16 mmol h^−1^ at 100 mA/cm^2^, with each sustained over the course of 5 h of operation (Fig. 5f). This corresponds to the removal of 1.9 and 3.4 g of CO_2_ from the carbonate capture liquid (Fig. 5f) from an input post-capture liquid that contained 13.2 grams of CO_2_.

## Discussion

In this work, we employed a template synthesis strategy to develop nanoconfined Ni single-atom catalysts with isotropic, periodically aligned pore architectures, enabling efficient retention, binding, and activation of *i*-CO_2_ in carbonate-fed reactive capture systems. The nanoconfinement effect, driven by short-range, non-electrostatic interactions between CO_2_ and pore walls, enhances *i*-CO_2_ accumulation and binding, intermediate stabilization, and *CO desorption, thereby boosting CO production. Through combined catalyst and system optimization, we achieved a high FE_CO_ of 50% ± 3% at 300 mA/cm^2^, >99% carbon utilization, and <1% CO_2_ in the outlet, and a record EE of 46% for 2:1 syngas. These results highlight the role of interconnected nanopores and anchored single-atom sites in controlling *i*-CO_2_ transport and reactivity under hydrophilic, reactant-starved electrolysis conditions.

## Methods

### Chemicals and materials

Nickel(II) acetylacetonate (95%), carbon tetrachloride (99.9%), ethylenediamine (99.5%), Pluronic P123 (average Mn~5800), oleylamine (OAm, >70%), octyltrimethylammonium bromide (OTAB, >98%), ammonium fluoride (NH_4_F, >98%), potassium carbonate (ACS reagent, ≥99.0%), and potassium hydroxide (ACS reagent, ≥85%) were purchased from Sigma-Aldrich. Tetraethyl orthosilicate (TEOS, 98%) was purchased from Gelest. Heptane (HPLC grade), *n*-butanol (ACS grade), hydrofluoric acid (trace metal grade), hydrochloric acid (trace metal grade), and nitric acid (trace metal grade) were purchased from Fisher Scientific. The bipolar membrane (Fumatech FBM) and hydrophilic carbon paper (Freudenberg H23) were purchased from Fuel Cell Store. Ni foam (1.6 mm Thick) was purchased from MTI Corporation. All chemicals were used as received without further purification. Deionized (DI) water (18.2 MΩ cm, Barnstead™ E-Pure™) was used for all experiments in this work.

### Synthesis of templates

Synthesis of SBA-15. For the SBA-15 template synthesis^51^, Pluronic P123 (4.0 g) was dissolved in 120 mL of 2.0 M HCl solution at 37 ^o^C in a 500 mL polypropylene bottle. Once fully dissolved, TEOS (8.5 g) was added, and the solution was stirred for 20 h at 37 ^o^C. The mixture was then transferred to an oven and kept at 100 °C for 24 h under static conditions. The resulting white precipitate was recovered by filtration, washed twice with DI water/methanol (1:1, v/v), and air-dried in a hood for 3 days. The dried solid was calcined at 550 ^o^C for 5 h in air with a ramping rate of 2 ^o^C/min. For the synthesis short channel SBA-15^52^, in a 500 mL polypropylene bottle, Pluronic P123 (2.4 g), NH_4_F (6.9 mg), and 1.94 M HCl (80 mL) were added. Once fully dissolved, a pre-mixed solution of TEOS (5.5 mL) and heptane (0.663 g) was added to the bottle, and the mixture was stirred for 4 min before being left under static conditions for 20 h. The mixture was then transferred to an oven and kept at 100 °C for 24 h under static conditions. The resulting white precipitate was recovered by filtration, washed twice with a 1:1 (v/v) mixture of DI water and methanol, and air-dried in a hood for 3 days. The dried solid was calcined at 550 ^o^C for 5 h in air with a ramping rate of 2 ^o^C/min.

Synthesis of MCM-48. For the MCM-48 template synthesis^53^, in a 500 mL round-bottom flask, OTAB (0.678 g) and F-127 (2.538 g) were dissolved in a mixture of ethanol (43.0 g), water (129.8 g), and ammonia solution (12.24 g, 30–33% NH_3_ in H_2_O). The mixture was stirred vigorously at 850 rpm until fully dissolved. Subsequently, TEOS (1.8 g) was quickly added to the flask. Upon addition, the mixture was stirred continuously for ~1.5 min, during which it changed from colorless to light blue. The reaction mixture was then left undisturbed at room temperature for 24 h. The resulting solid was collected by centrifugation at 8000 rpm for 10 min, washed three times with ethanol (35 mL each), and dried under vacuum at room temperature overnight. The obtained powder was then calcinated at 550 ^o^C for 6 h under air at a ramping rate of 5 ^o^C/min.

Synthesis of KIT-6. For the KIT-6 template synthesis^54^, Pluronic P123 (4.0 g) was dissolved in a mixture solution of 144 mL of DI water and 7.9 mL of 35 wt. % hydrochloric acid. After which, 4.0 mL of *n*-butanol was added. This mixture was then stirred vigorously for 1 h at 35 °C. Following this, 8.6 mL of TEOS was added, and the solution was stirred continuously for another 24 h. The mixture was then subjected to hydrothermal synthesis at 100 °C for 24 h. Afterward, the resultant solid was filtered and washed with DI water. The obtained solid was then calcinated at 550 °C for 6 h.

### Synthesis of Ni-NACs within silica templates

To a 50 mL round bottom flask, 1.8 g of ethylenediamine and 25–100 mg of nickel (II) acetylacetonate were added and mixed for 5 min. 4.0 g of carbon tetrachloride was added and stirred for 5 min. Subsequently, 0.8 g of silica template (SBA-15, KIT-6, or MCM-48) was introduced, and the mixture was refluxed at 90 °C for 16 h before being heated at 130 °C for 2 h to remove any residual volatiles. The resultant residue was then heated under a flowing Argon atmosphere with a ramping rate of 3 °C/min to 800 °C and remained at 800 °C for 2 h. The resulting black powder was dispersed in a solution of 5 wt.% HF and 10 wt.% HCl (35 mL) and stirred for 24 h. The remaining solid materials were collected by centrifugation, which was then washed with deionized water until a neutral pH was achieved. Finally, the final carbon material was dried at 80 °C overnight and stored for future use. The carbon materials synthesized using SBA-15, KIT-6, and MCM-48 are used directly as porous catalysts, named DirectionalMeso, IsotropicMeso, and IsotropicNano, respectively.

Post-synthesis thermal treatment of Ni-NAC was carried out under a diluted NH_3_ gas atmosphere. Typically, the as-prepared Ni-NAC was annealed at 700 ^o^C for 1 h under a flow of 10% NH_3_ in He, with a heating rate of 5 ^o^C/min. After annealing, the sample was naturally cooled to room temperature under the same gas atmosphere. The resulting material was used directly as the catalyst.

### Electrochemical measurements

The flow electrolyzer contains two stainless steel flow-field plates with serpentine channels, PTFE and silicone gaskets, and the MEA, which contains two electrodes and a membrane, and was formed after assembling the cell hardware. The catholyte and anolyte were circulated by peristaltic pumps (INTLLAB) at 20 ml min^–1^. The applied current was controlled by a Bio-Logic VSP 300 potentiostat/galvanostat. All reported cell voltages and electrode potentials are presented without iR compensation. The membrane used to separate catholyte and anolyte was a commercial (Fuel Cell Store) or custom-designed BPM. The as-prepared catalyst was used as the cathode. A piece of filter membrane was inserted as the interposer layer between cathode and cation exchange layer (CEL). The interposer layer was a hydrophilic mixed cellulose ester (MCE) membrane with a controlled thickness of 135 µm and a pore size of 8 µm. The catholyte was 1.5 M K_2_CO_3_, and the anolyte was 1 M KOH. All experiments were performed at room temperature.

The catalyst ink was prepared by dispersing Ni-NAC catalysts in 2-propanol solvent with added Nafion ionomer by ultrasonication. The ink needs to be well-sonicated for a good dispersion of catalyst. The mass ratio of the catalyst and ionomer was 9:1. The ink was then airbrushed onto the substrate to the final loading of ~1.5 mg cm^–2^.

NiFeP electrode as anode was prepared from a modified method in the literature^55^. Ni foam was first cleaned by 6 M HCl and DI-water for 15 min under sonication. Then, a 40 mL solution with 4 mmol NH_4_F, 10 mmol urea, 2 mmol Ni(NO_3_)_2_·6H_2_O, and 2 mmol Fe(NO_3_)_3_·9H_2_O was prepared and transferred to a 50 mL Teflon-lined stainless steel autoclave. The hydrothermal growth of the hydroxides on Ni foam was performed at 120 °C for 6 h with a heating rate of 3 °C min^−1^, followed by sonication in DI-water and drying in the oven at 80 °C to obtain NiFeOx catalyst. To prepare NiFeP, NiFeOx and 1.0 g of NaH_2_PO_2_·H_2_O were placed in a ceramic boat inside a tube furnace, with NaH_2_PO_2_·H_2_O positioned upstream relative to the gas flow. After purging with argon (Ar), the center of the furnace was elevated to 300 °C at a ramping rate of 1 °C min^−1^ and kept at this temperature for 2 h in a static Ar atmosphere, and then naturally cooled down to ambient temperature.

The gas-phase CO_2_ reduction reaction in a three-compartment flow cell was carried out using an Ag/AgCl (4 M KCl) reference electrode and a Ni foam counter electrode. The cathode catalysts were applied onto hydrophobic carbon paper (Freudenberg H23C3) via airbrushing. A 40-micron anion-exchange membrane (PiperION) was used to separate the cathode and anode chambers. Both the catholyte and anolyte consisted of 1.5 M K_2_CO_3_ solution.

For experiments involving varying CO_2_ flow rates, a mass flow controller (Alicat Scientific) was used to regulate the gas flow. In studies with different CO_2_ concentrations, N_2_ was used as a balance gas to adjust the partial pressure of CO_2_. The gases were mixed using a tee-type connector before being introduced into the flow cell. The total gas flow rates were measured with a gas flow meter. Product concentrations at the catholyte outlet were quantified using gas chromatography (GC).

For gas-phase CO_2_ reduction in a membrane electrode assembly (MEA) setup, the device was constructed by sequentially assembling a cathode gas diffusion electrode (GDE, 4 cm² geometric area), a PiperION anion-exchange membrane, and an anode (IrO_2_-GDE, 4 cm², Dioxide Materials). These components were secured within PTFE gaskets, each with a 4 cm^2^ window, and the assembly was compressed between electrolyzer plates to ensure proper sealing, electron conductivity, and ionic transport.

Humidified CO_2_ gas, controlled by a mass flow controller (Alicat Scientific), was introduced into the cathode flow field for the reaction. The product stream was mixed with a 2.5 mL/min N_2_ stream to facilitate analysis by GC. On the anode side, a 0.1 M KHCO_3_ solution was circulated at a flow rate of 20 mL/min. In MEA configurations operating at high current densities, significant CO_2_ loss due to (bi)carbonate formation was mitigated by using the mixed N_2_ stream as an internal standard for accuracy. We define the single-pass (SP) efficiency as follows:

2 $${{\rm{Single}}}\; {{\rm{Pass}}}\; {{\rm{Efficiency}}}({{\rm{SP}}}):{{\rm{SP}}}=\frac{{\dot{V}}_{{CO}}}{{\dot{V}}_{{CO}2,{in}}}\times 100\%$$

Where ${\dot{V}}_{{CO}}$ and ${\dot{V}}_{{CO}2,{in}}$ represent the volume flow rates of the produced CO and the total CO_2_ inlet, respectively.

### Product analysis

The gas products (H_2_ and CO) were quantified by GC (Shimadzu2014, PerkinElmer Clarus 580) equipped with a thermal conductivity detector (TCD) and a flame ionization detector (FID) equipped with a Methanizer. The calibration curve was established by analyzing the standard calibration gases with different concentrations (10–10,000 ppm). Argon (50 mL min^-1^) was purged as the carrier gas to carry the gas products out of the system for quantification.

The rate of H_2_/CO generation (*r*, mol s^−1^) for each cycle was calculated by the following equation:

3 $$r=c \times {10}^{-6} \times [{P} {\dot v} \times {10}^{-6} / (RT)]$$

Where *c* is the H_2_/CO concentration (ppm); *V̇* is the volumetric flow rate of the inlet gas (100 ml min^−1^); *p* is the ambient pressure (*p* = 1.013 × 10^5 ^Pa); *R* is the gas constant (*R* = 8.314 J mol^−1^ K^−1^); *T* is the room temperature (293.15 K). The total amount of gas (mol) was calculated by integrating the plot of H_2_/CO production rate (mol s^−1^) *vs*. reaction time (s).

Faradaic efficiency (FE_*i*_) can be calculated by equations as follows:

4 $${{{\rm{FE}}}}_{i}=\frac{{n}_{i}{z}_{i}F}{Q}\times 100\,\%$$

Where *n*_0_ is initial moles of reactant; *n* is the moles of reactant after electrolysis; *n*_*i*_ is the moles of product *i*; *z*_*i*_ is the number of electrons transferred for one product molecule; *F* is the Faraday constant (96,485 C mol^−1^); *Q* is the total charge passed through the electrolytic cell; t is the electrolysis time (s).

### Materials characterization

Field-Emission Scanning Electron Microscope (FE-SEM) was recorded on the FEI Teneo LoVac. Aberration-corrected HAADF STEM imaging was performed using a probe-corrected Thermo Fisher (FEI) Titan Themis. Powder X-ray diffraction (XRD) patterns of samples were recorded on a Bruker X-ray diffractometer using Cu Kα radiation (40 kV, 40 mA) over the range of 5–80 of 2θ°. X-ray photoelectron spectrometry (XPS) was recorded on a PerkinElmer PHI ESCA system with Physical Electronics (PHI). Nitrogen sorption isotherms were measured by the Micromeritics 3Flex analyzer at −196 °C. Before recording the N_2_ sorption isotherms, the samples were pretreated at 200 °C under vacuum for 12 h. The total surface area was calculated using the Brunauer–Emmett–Teller (BET) equation. The micropore and mesopore surface area, and micropore volume were evaluated using t-plot method. The nanopore size was determined using the Horvath-Kawazoe method, with measurements conducted on a Micromeritics Tristar instrument. The mesopore size was determined using the Horvath-Kawazoe method, with measurements taken on a Micromeritics 3Flex instrument. Inductively coupled plasma optical emission spectroscopy (ICP-OES) for nickel loadings was performed using an Agilent 5800 spectrometer. Elemental analysis of carbon and nitrogen was measured on a Thermo FlashSmart CHNS/O Elemental Analyzer. SAXS measurements were collected at beamline 12-ID-B of the Advanced Photon Source (APS) at Argonne National Laboratory. In situ ATR-SEIRAS spectroscopy was performed using a Thermo Scientific Nicolet iS50 spectrometer equipped with a custom-designed ATR electrochemical cell. The spectral resolution was set to better than 2 cm⁻¹, with 64 scans accumulated per spectrum. All other experimental parameters were configured according to the standard test protocol provided by the manufacturer or experimental requirements. The electrolysis was performed in a three-electrode system using an Ag/AgCl reference electrode, with CO_2_-saturated 0.5 M KHCO_3_ as the electrolyte. The conversion of potentials from Ag/AgCl to the RHE scale was performed using the following equation:

5 $${E}_{{{\rm{RHE}}}}={E}_{{{\rm{Ag}}}/{{\rm{AgCl}}}}+0.197+0.059\times {{\rm{pH}}}$$

Electrochemical mass spectrometry (EC-MS) was conducted using our carbonate-fed gas-tight electrochemical cell coupled to a quadrupole mass spectrometer (Thermo Scientific™). The evolved gases were continuously directed from the headspace of the cathode chamber into the mass spectrometer via a heated capillary inlet maintained at 120 °C to prevent condensation. Signals corresponding to CO_2_ (m/z = 44) were recorded under steady-state conditions at applied current densities ranging from 50 to 300 mA/cm^2^. The background signal was subtracted.

### Computational methods

#### The calculation of CO_2_ adsorption energy to the Ni-SAC surface

All density functional theory (DFT) calculations were performed using the Vienna ab initio simulation package (VASP). The Perdew-Burke-Ernzerh (PBE) exchange-correlation functional and the projector augmented wave (PAW) methods were adopted. The spin polarization was turned on for all simulations. The dispersion interactions were included using Grimme’s D3 correction. The kinetic cutoff energy for the plane-wave basis set was set as 520 eV. We used a *k*-point sampling of 5 × 5 × 1 with the Monkhorst-pack scheme for integration over the Brillouin zone in reciprocal space. The convergence criteria for the electronic and ionic loops were set at 10^−5^ eV and 0.02 eV Å^−1^, respectively.

A set of supercells was constructed with a 20 Å-thick vacuum slab to separate the graphene layer from its periodic images in the *z*-direction. The graphene unit cell dimensions were optimized to be *a* = *b* = 2.46 Å and *γ* = 120°. The 4 × 4 supercell of graphene was used to construct the 4N-Gr support. A C-divacancy was created first. Then, four C atoms on the vacancy edge were replaced by four N atoms. After that, one Ni atom was embedded into the central coordination site to generate the Ni@4N-Gr structure. After the structural relaxation in DFT calculations, the Ni metal atom remains in the same plane as the C atoms. This configuration is consistent with the reported results^36,56^.

The adsorption energy *E*_ads_ of an adsorbed species (i.e., CO_2_ or H_2_O) on the Ni@4N-Gr is calculated as:

6 $${E}_{{{\rm{ads}}}}={E}_{{{\rm{mol}}}+{{\rm{Ni}}}-{{\rm{SAC}}}}-({E}_{{{\rm{mol}}}}+{E}_{{{\rm{Ni}}}-{{\rm{SAC}}}})$$

where ${E}_{{{\rm{mol}}} {+} {{\rm{Ni}}}-{{\rm{SAC}}}}$, ${E}_{{{\rm{Ni}}}-{{\rm{SAC}}}}$, and ${E}_{{{\rm{mol}}}}$ are the energies of the catalysis with the molecule adsorbed, of the bare catalyst, and of the molecule, respectively.

#### The calculation of CO_2_ in-pore concentration and local number distribution in nanopores

Figure 3b shows the representative modeling systems in our MD simulations. where a nanoslit formed by two parallel catalyst sheets was assumed to be rigid and in contact with aqueous electrolyte solution reservoirs at two sides. The slit walls have a dimension of 3.34 × 2.99 nm laterally, and the width of the reservoir is around 12 nm. Periodic boundary conditions were applied in all three directions. Different slit sizes were studied (defined as the distance between the atomic centres of neighbored graphene sheets), including 10.0 nm, 1.5 nm, and 1.0 nm, respectively. The aqueous electrolyte solution was composed of CO_2_ and water. The ionic charge values were determined using the DDEC6 analysis^57^ based on the DFT calculation results (summarized in Supplementary Table 6). To model the CO_2_ adsorption affinity towards catalyst surfaces, we optimized the force field parameters using an in-house generic algorithm code (GA)^58^. The specific parameter values are summarized in the Supplementary Table 7.

## Supplementary information

Supplementary information

Transparent Peer Review file

## Source data

Source data

## Data Availability

All the data that support the findings of this study are available in the main text and the Supplementary Information, or from the corresponding authors upon reasonable request. Source Data file for DFT and MD structures has been deposited in Figshare under accession code DOI link 10.6084/m9.figshare.29323862.v1. Source data are provided with this paper.

## Code availability

All data and information for the simulations are available in the supplementary materials. Any additional information is available from the corresponding authors upon a reasonable request.
