# Supplementary material for: Interconnected nanoconfining pore networks enhance catalyst CO2 interaction in electrified reactive capture
Source: Nat Commun. 2025 Jul 4;16:6185. doi: 10.1038/s41467-025-61407-8 (PMC12227553; doi:10.1038/s41467-025-61407-8)
Supplement: Supplementary file 1 — Supplementary information [file 41467_2025_61407_MOESM1_ESM.pdf]

Interconnected Nanoconfining Pore Networks Enhance Catalyst CO_2 Interaction in Electrified Reactive Capture

Hengzhou Liu[1†], Lun An[2†], Peiyao Wang[3†], Christine Yu[1†], Jie Zhang[2], Heejong Shin[1], Bosi Peng[1], Jiantao Li,[1] Matthew Li,[4] Hongmin An,[1] Jiaqi Yu,[1] Yuanjun Chen[1], Peiying Wang[1], Kug-Seung Lee[5], Kanika Lalit[6], Zeyan Liu[1], Omar K. Farha,[1] Wenyu Huang[2, 6], Jefferson Zhe Liu[3,*], Long Qi[2,*], Ke Xie[1,*], Edward H. Sargent[1,7,*]

[1]Department of Chemistry, Northwestern University, 2145 Sheridan Road, Evanston, IL 60208, United States

[2]U.S. DOE Ames National Laboratory, Iowa State University, Ames, IA 50011, United States

[3]Department of Chemical Engineering, The University of Melbourne, Parkville, Melbourne, VIC 3052, Australia

[4]Chemical Sciences and Engineering Division, Argonne National Laboratory, Lemont, IL 60439, USA

[5]Pohang Accelerator Laboratory (PAL), Pohang University of Science and Technology (POSTECH), Pohang 37673, Republic of Korea

[6]Department of Chemistry, Iowa State University, Ames, IA, 50011, United States

[7]Department of Electrical and Computer Engineering, Northwestern University, 2145 Sheridan Rd, Evanston, IL 60208, United States

[†]These authors contributed equally.

*Corresponding email: ted.sargent@northwestern.edu
ke-xie@northwestern.edu
lqi@iastate.edu
zhe.liu@unimelb.edu.au

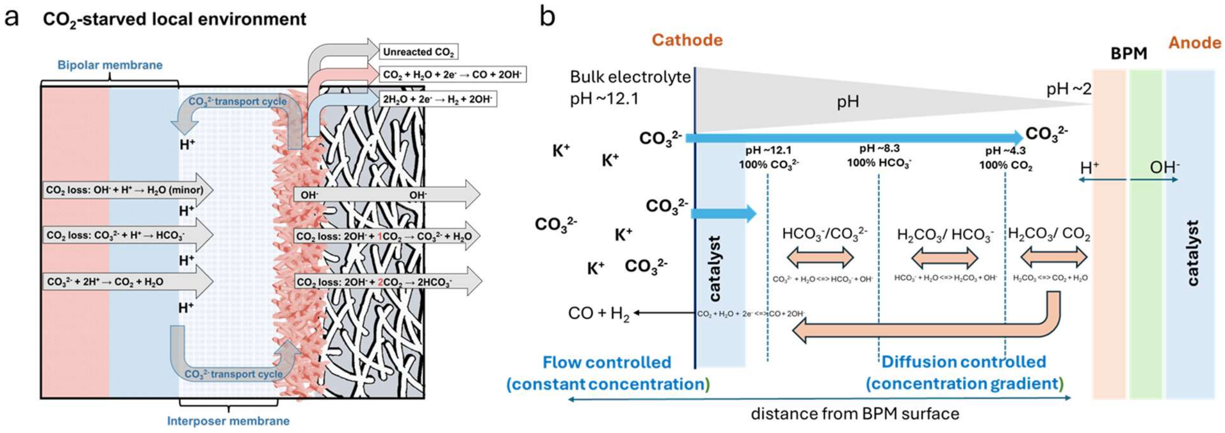

Supplementary Figure 1. Schematic illustration of the local environments at the catalyst and BPM interface, including (a) the chemical environment and (b) the pH distribution.

At a specific current density, the supply of *i*-CO_2 from the membrane interface to the cathode surface in the carbonate electrolyzer becomes increasingly constrained by the following two reactions:

$$CO_3^{2-}(aq) + H^+ \rightleftharpoons HCO_3^-(aq)$$

$$OH^- + H^+ \rightarrow H_2O$$

These two side reactions may consume H^+, with each H^+ generation requiring the passage of one electron through the electrolyzer. These reactions compete with the desired *i*-CO_2 generation process:

$$CO_3^{2-} + 2H^+ \rightarrow CO_2 + H_2O$$

The *i*-CO_2 loss is also constrained by the locally high alkalinity, particularly under electrolysis conditions, where a pronounced pH gradient exists—from approximately pH 2 at the BPM interface to greater than 12 at the catalyst surface.[1] The side reactions occurred are as follows:

$$CO_2(g) + OH^-(aq) \rightleftharpoons HCO_3^-(aq)$$

$$CO_2(g) + CO_3^{2-}(aq) + H_2O(l) \rightleftharpoons 2HCO_3^-(aq)$$

$$CO_2(g) + 2OH^-(aq) \rightleftharpoons CO_3^{2-}(aq) + H_2O(l)$$

These reactions severely deplete the local *i*-CO_2, creating challenges for its further conversion into the desired products.

Supplementary Note 1. Theoretical *i*-CO_2 supply rate in the carbonate system

Local generation of i-CO_2 in carbonate system

In process of using KOH as the DAC mediate, carbonate species predominate in the post-capture solution: [CO_3^{2-} (aq)] >> [HCO_3^- (aq)].[2] For carbonate electrolysis, the liquid form of CO_3^{2-} should be first converted into *in situ* CO_2 (g) and CO_2 (aq) for their continuous reduction into carbon products.

In the BPM-based carbonate electrolyzer, the *i*-CO_2 generation is based on the following equation:

$$\mathrm{CO_3^{2-} + 2H^+ \rightarrow CO_2 + H_2O}$$

At 100 mA, the flow rate of CO_2 in carbonate system is calculated as follows:

$$\text{Flow rate}_{\mathrm{CO2}} = \frac{0.1\ \mathrm{A\ cm^{-2}} * 1\ \mathrm{cm^2} * 60s * 22.4\ \mathrm{L\ mol^{-1}}}{96485 * 2} * 1000 = 0.69\ \mathrm{mL \cdot min^{-1}}$$

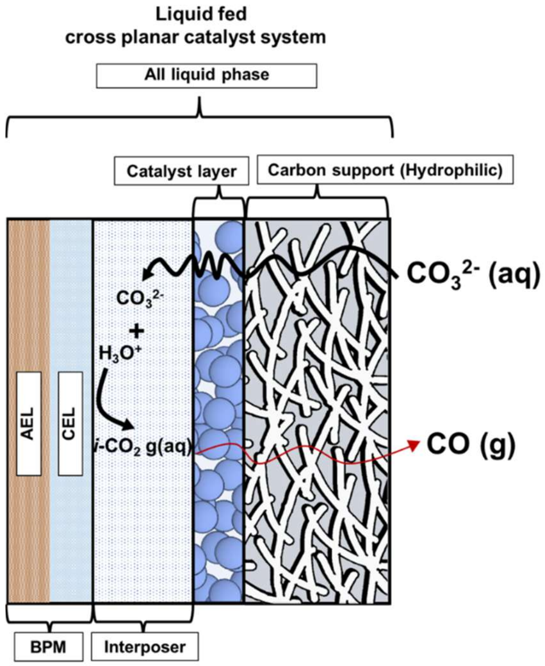

Supplementary Figure 2. Schematic illustration of liquid-fed reactive capture systems in BPM electrolyzers with a substantially planar catalyst layer supported on carbon. The *i*-CO_2 is not fully utilized and can either be removed out of the system or react with the locally alkaline environment, resulting in low *i*-CO_2 utilization and reduced CO selectivity.

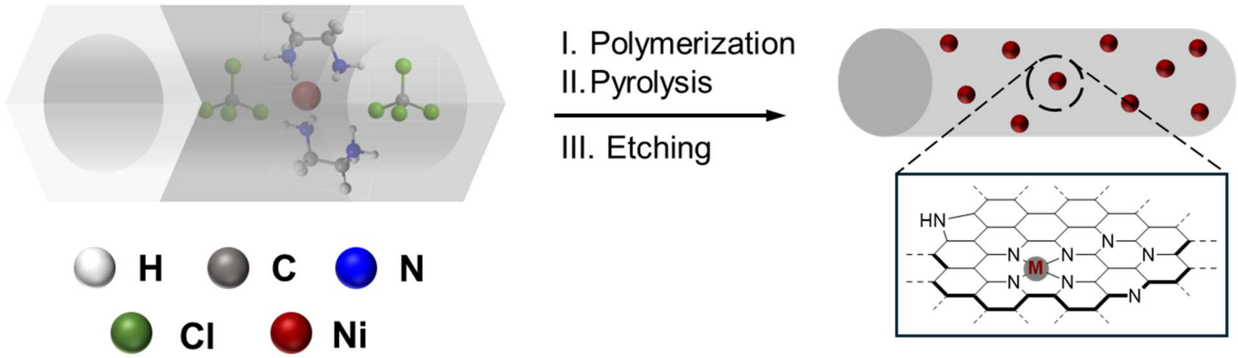

Supplementary Figure 3. Schematic illustration of the templated synthesis process.

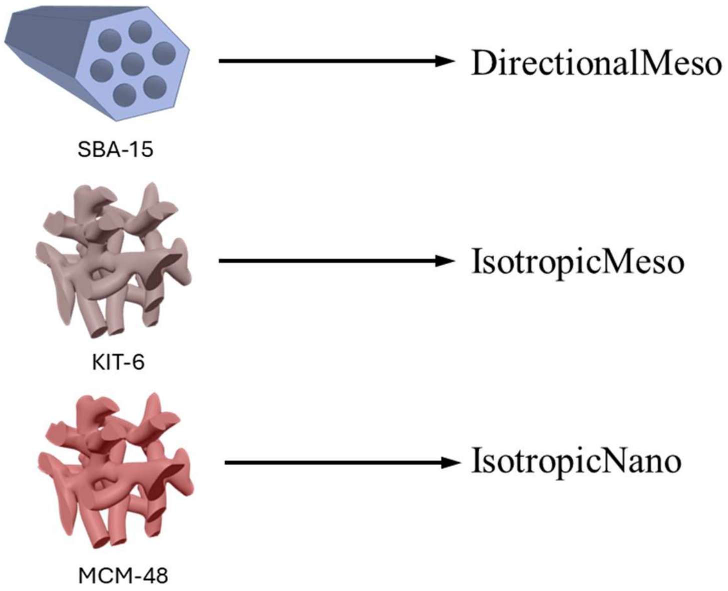

Supplementary Figure 4. Schematic illustration of the synthesis of three catalysts, DirectionalMeso, IsotropicMeso, and IsotropicNano through the use of templates of SBA-15, KIT-6, and MCM-48, respectively.

Supplementary Table 1. Summarize the properties of SBA-15, KIT 6, and MCM 48 templates.

**Property**	**SBA-15**	**KIT-6**	**MCM-48**
Type	Mesoporous silica	Mesoporous silica	Mesoporous silica
Pore Structure	Hexagonal (2D, P6mm symmetry)[3]	Cubic (3D, la3d symmetry)[4]	Cubic (3D, la3d symmetry)[5]
Pore size	4-30 nm	4-12 nm	2-10 nm

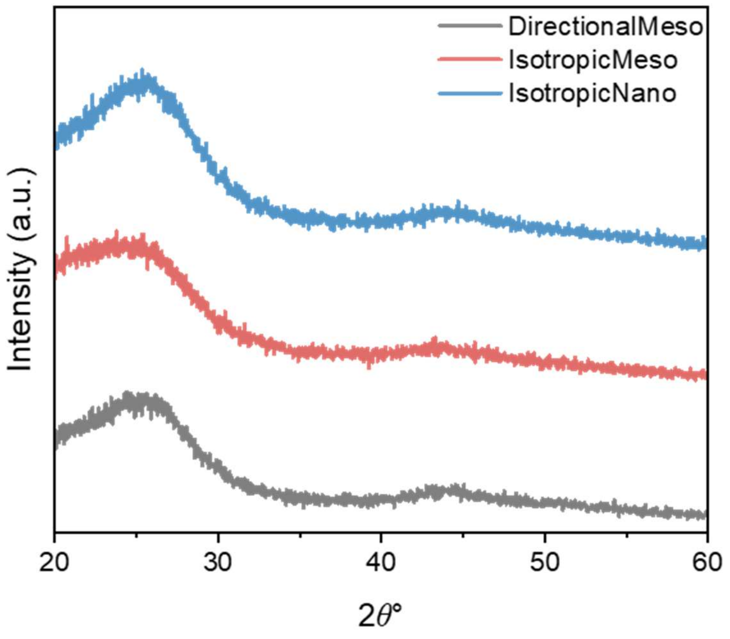

Supplementary Figure 5. X-ray diffraction patterns. The X-ray diffraction patterns of the catalysts exhibited a broad peak centered at 25.6°, corresponding to the (002) plane of graphitic carbon.[6] Notably, no peaks associated with Ni particles were detected in any of the three catalysts.

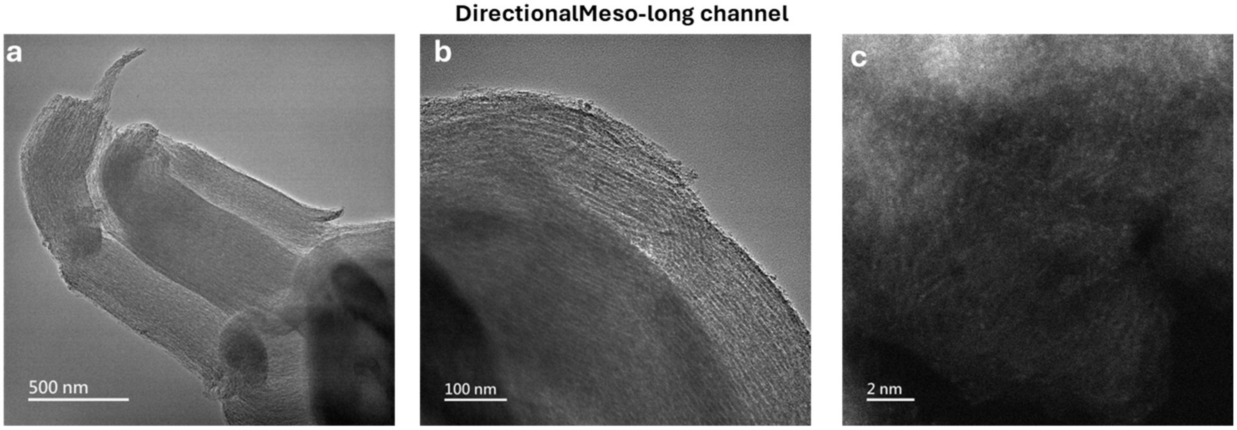

Supplementary Figure 6. (a)-(b) Low-magnification STEM image, and (c) Atomic-resolution HAADF-STEM images of DirectionalMeso with long channels.

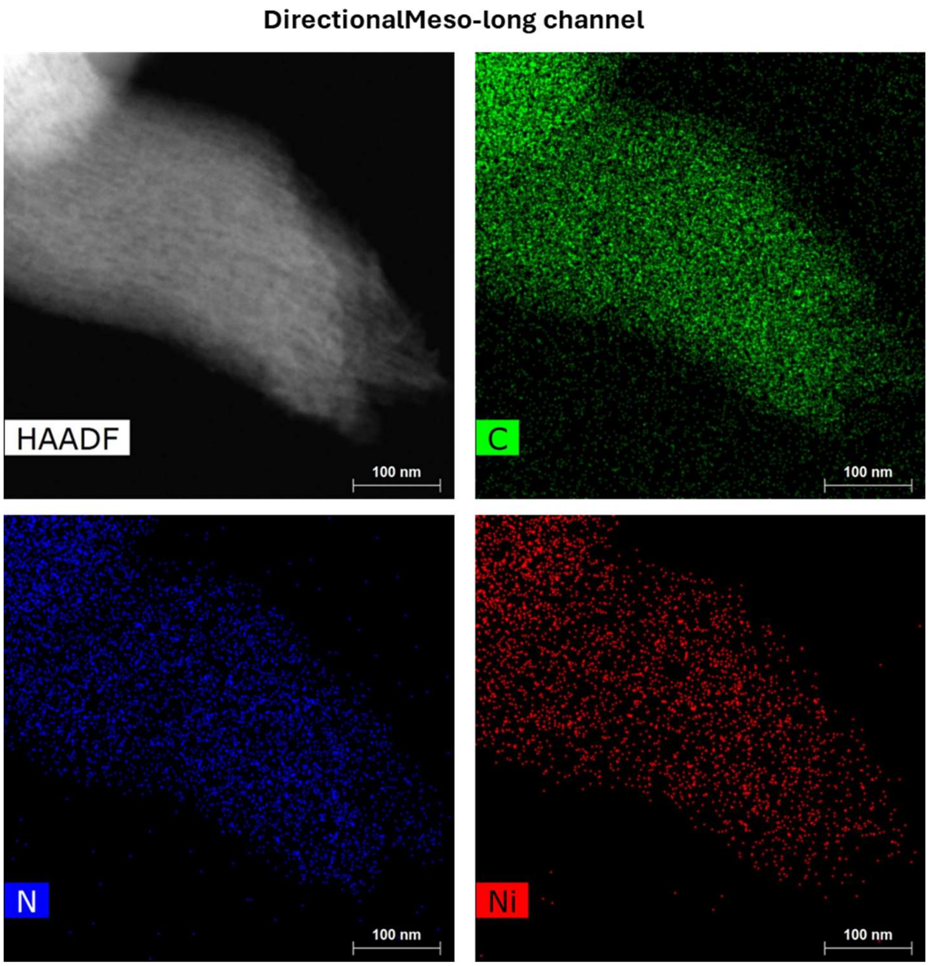

Supplementary Figure 7. HAADF-STEM-EDS mapping of DirectionalMeso with long channels.

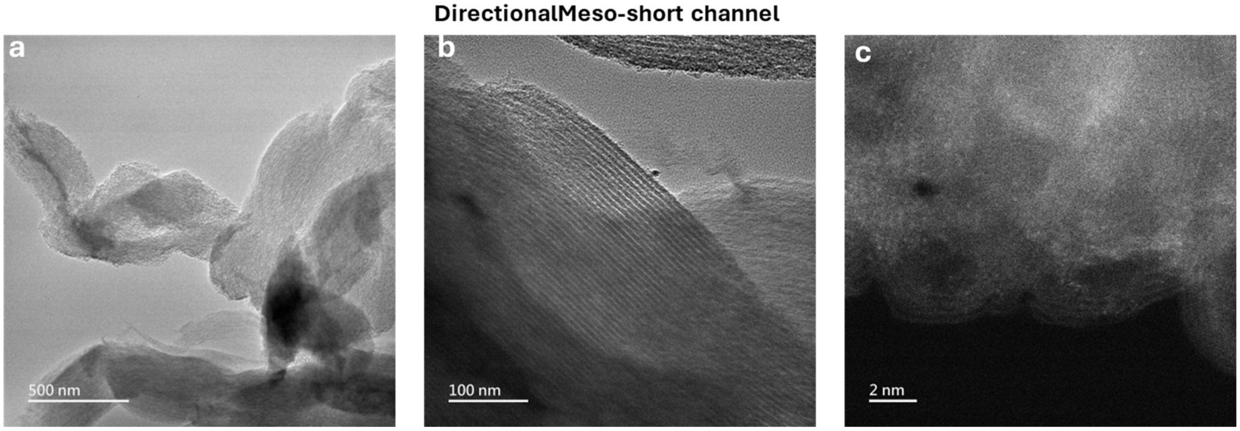

Supplementary Figure 8. (a)-(b) Low-magnification STEM image, and (c) Atomic-resolution HAADF-STEM images of DirectionalMeso with short channels.

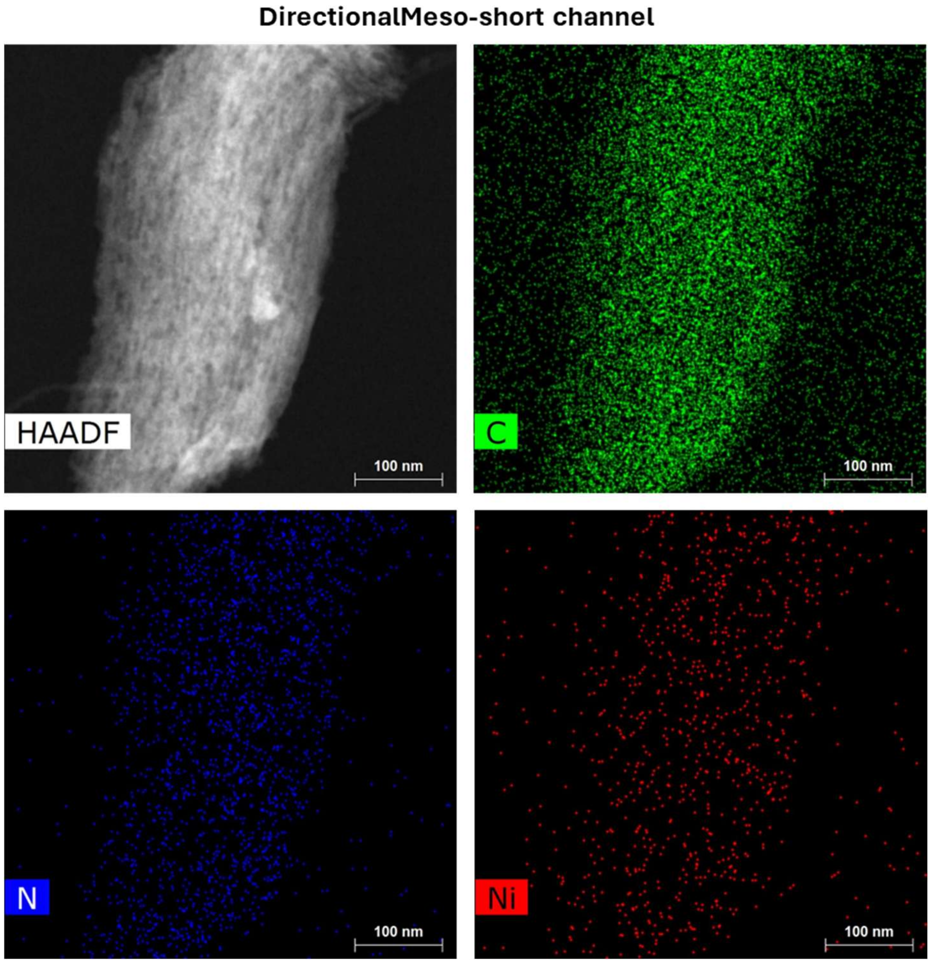

Supplementary Figure 9. HAADF-STEM-EDS mapping of DirectionalMeso with long channels.

IsotropicMeso

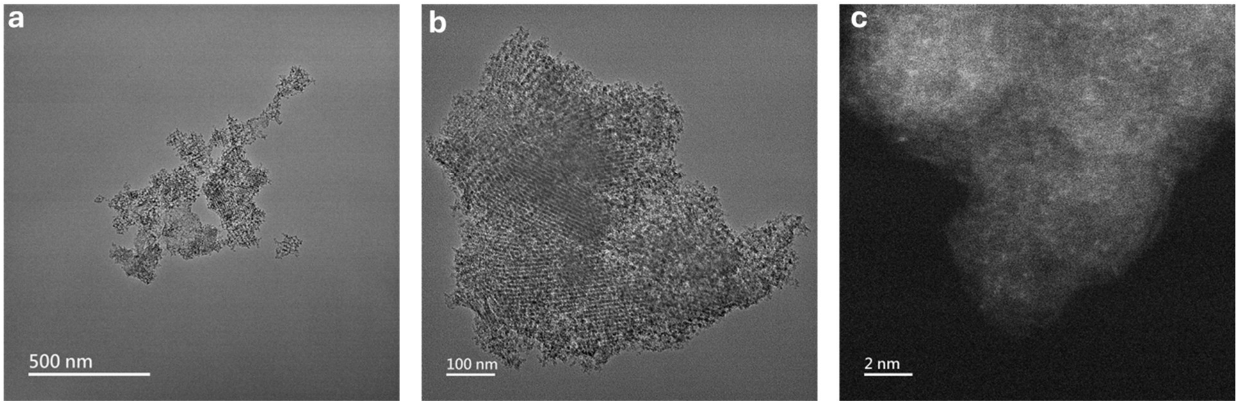

Supplementary Figure 10. (a)-(b) Low-magnification STEM image, and (c) Atomic-resolution HAADF-STEM images of IsotropicMeso.

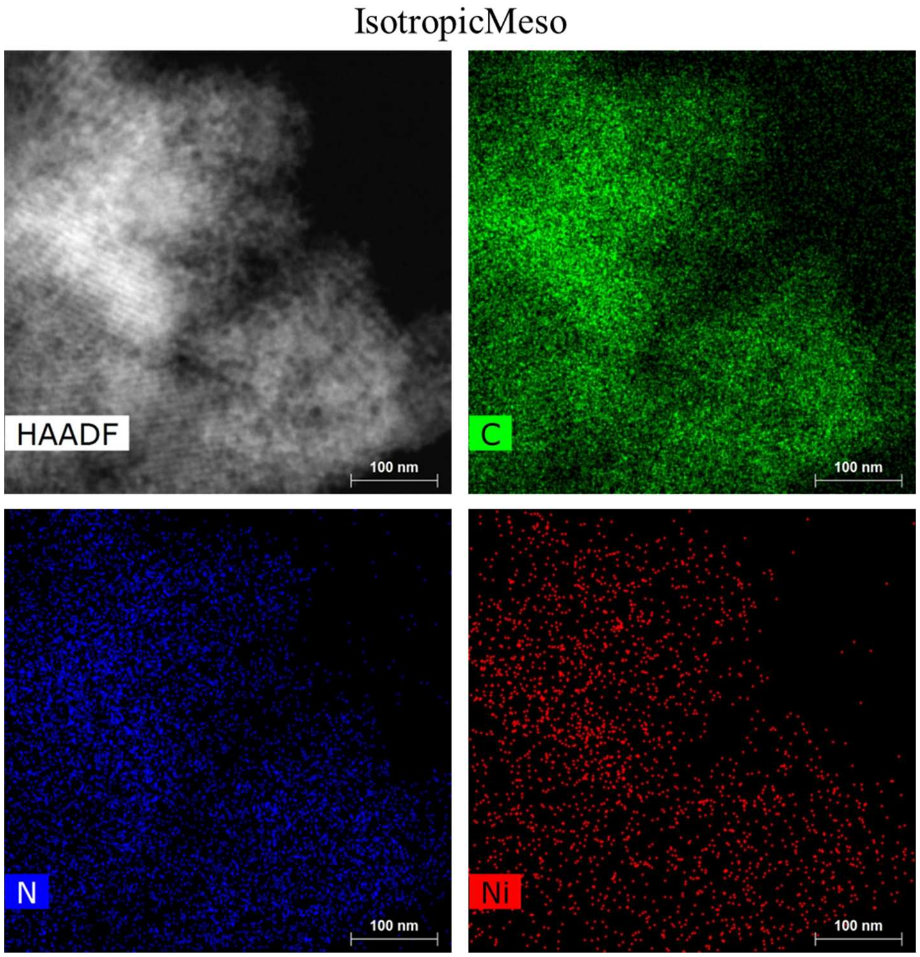

Supplementary Figure 11. HAADF-STEM-EDS mapping of IsotropicMeso.

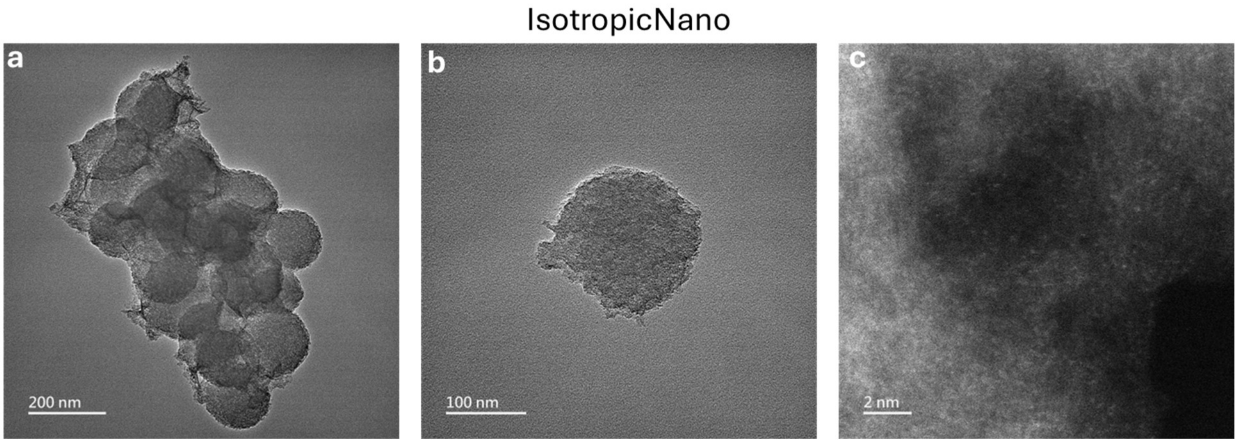

Supplementary Figure 12. (a)-(b) Low-magnification STEM image, and (c) Atomic-resolution HAADF-STEM images of IsotropicNano.

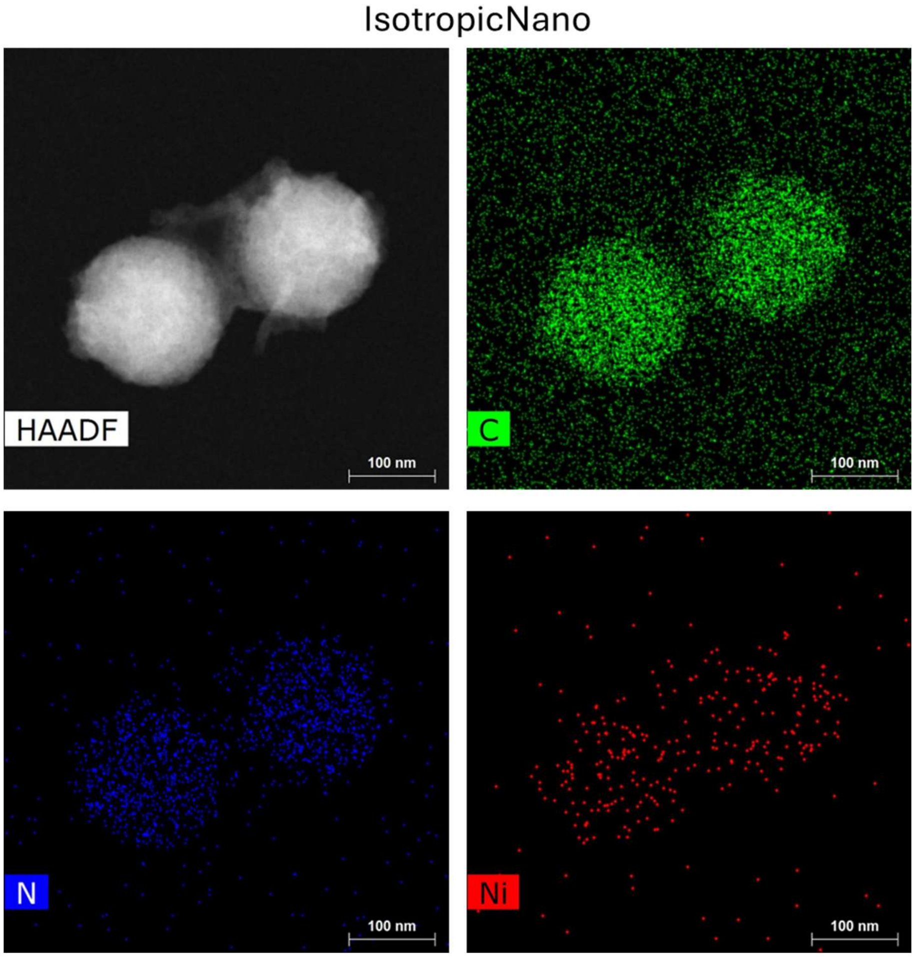

Supplementary Figure 13. HAADF-STEM-EDS mapping of IsotropicNano.

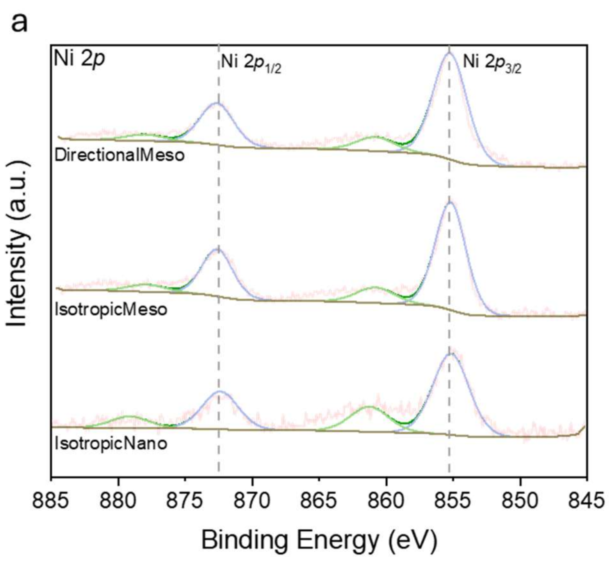

Supplementary Figure 14. High-resolution XPS (a) Ni 2p and (b) N 1s spectra.

The N 1s spectra indicated the presence of four distinct types of nitrogen, which can be attributed to pyridinic N (~398.5 eV), pyrrolic N (~399.8 eV), graphitic N (~400.9 eV), and oxidized N (~402.5 eV).[7]

The Ni 2p regions of all samples exhibited comparable binding energies at ~855.2 eV, which is higher than that of metallic Ni (852.6 eV), suggesting similar oxidation states for the Ni single atoms.[8]

Supplementary Table 2. Summarize the N content in different catalysts.

**Catalyst**	**N content from elemental analysis (wt%)**
DirectionalMeso Short channel	9.2
DirectionalMeso Long channel	9.5
IsotropicMeso	9.9
IsotropicNano	10.2

Supplementary Note 2. Synchrotron-based Small-Angle X-ray Scattering (SAXS) analysis

Synchrotron-based Small-Angle X-ray Scattering (SAXS) is a highly effective technique for probing the nanostructure of porous materials.[9-11] It enables the characterization of pore size distribution within the mesoporous (2–50 nm) and microporous (<2 nm) ranges. By applying models such as Porod analysis,[9] SAXS allows for the analysis of pore distribution. When combined with BET analysis, it provides a more comprehensive understanding of pore structure and surface area.

Beyond pore size distribution, SAXS is instrumental in assessing structural correlation and ordering within the material.[11] The presence of correlation peaks in the SAXS profile indicates periodicity and symmetry in the arrangement of pores, revealing whether the network exhibits an ordered or disordered structure.

The Porod region in SAXS data further provides insights into surface roughness and fractal characteristics of the pore network.[11] Mass fractal behavior is often associated with hierarchical or interconnected porous architecture.[5]

(1) Fractal Analysis

Fractal analysis in SAXS is used to describe the structural complexity, roughness, and hierarchical organization of porous materials.[10] It provides insight into whether the scattering objects exhibit self-similarity over different length scales.

Mass fractal structures refer to geometries where the mass of a porous network scales non-linearly with size, typically following a power law. This implies the material exhibits self-similarity across different length scales—i.e., its structure looks "similar" when zoomed in or out. The fractal dimension (D) between 1 and 3 reflects how densely the material fills space:

- D ~ 1 indicates a more linear or fibrous structure,
- D ~ 2 implies a surface-like, rough structure,
- D ~ 3 approaches a space-filling, dense structure.

Fractal materials exhibit a power-law dependence in SAXS intensity.

$$I(q) \propto q^{-D}$$

We used mid-to-high q range (q=0.01 to 0.1 $Å^{-1}$) to capture the scaling behavior of intensity.

Taking the logarithm:

$$\ln\, I(q) = -D \ln\, q + C$$

Here, D is the fractal dimension (surface or mass fractal), and C is a constant.

$$\ln(q) = \ln(\text{ Scattering Vector })$$

$$\ln(I) = \ln(\text{ Intensity })$$

We plotted the linear fitting in log-log scale (**Supplementary Figure 15**):

The analysis revealed that all samples exhibited D within the range of 1 to 3, indicating a mass fractal structure. This suggests the presence of a porous network, where the material's internal structure demonstrates self-similarity, validating our template synthesis approach and the periodic replication of the pore morphology. Additionally, the relatively high D values point to increased surface roughness and a more disordered pore arrangement.

(2) Analysis of periodicity in the arrangement of pores

For ordered porous materials, the peaks in SAXS patterns (**Figure 2d**) correspond to characteristic pore-to-pore distances, which can be used to estimate the periodic pores.

The interplanar spacing (d) associated with the scattering peak is calculated using Bragg's Law:

$$d_{hkl} = \frac{2\pi}{q_{hkl}}$$

d_{hkl} is the interplanar spacing corresponding to the first SAXS peak (Å); $q_{hkl}$is the Scattering vector of the first diffraction peak (Å^{-1}); hkl refers to the Miller indices of the peak.

For different structures, the first peak corresponds to different Miller indices:

- SBA-15 (2D hexagonal, used in sample DirectionalMeso) is d_{10}
- KIT-6 and MCM-48 (3D cubic, used in samples IsotropicMeso and IsotropicNano, respectively) are d_{211}

The pore diameter is estimated differently for hexagonal (SBA-15) and cubic (KIT-6, MCM-48) structures.

For SBA-15, the pore diameter D is derived from the hexagonal unit cell:

$$D = \frac{2}{\sqrt{3}} d_{10} - t$$

where: $\frac{2}{\sqrt{3}} d_{10}$is the approximate pore center-to-center distance; t is the wall thickness (~8 Å, assumed based on literature[12].

For cubic mesoporous structures (KIT-6 & MCM-48), the pore diameter is:

$$D = d_{211} - t$$

$d_{211}$is the Interplanar spacing for the (211) reflection in cubic phases; t is the wall thickness (~8 Å).

For SBA-15 (DirectionalMeso), the first peak corresponding to q = 0.046 Å;

$$d_{10} = \frac{2\pi}{q_{10}} = \frac{2\pi}{0.046} = 136.59\text{Å}$$
$$D = \frac{2}{\sqrt{3}} \times 136.59 - 8$$
$$D = 149.64\text{Å}(14.96\text{ nm})$$

For KIT-6 (IsotropicMeso), the first peak corresponding to q = 0.075 Å.

$$d_{211} = \frac{2\pi}{q_{211}} = \frac{2\pi}{0.075} = 83.78\text{ Å}$$
$$D = d_{211} - 8$$
$$D = 75.78\text{ Å}(7.58\text{ nm})$$

For MCM 48 (IsotropicNano), the first peak corresponding to q = 0.15 Å.

$$d_{211} = \frac{2\pi}{q_{211}} = \frac{2\pi}{0.15} = 41.89\text{Å}$$
$$D = d_{211} - 8$$
$$D = 33.89\text{Å}(3.39\text{nm})$$

The smallest reproduced pore diameter of 3.4 nm observed in IsotropicNano aligns with its intrinsically interconnected cubic pore structure. This highly ordered 3D bicontinuous network facilitates efficient molecular transport.

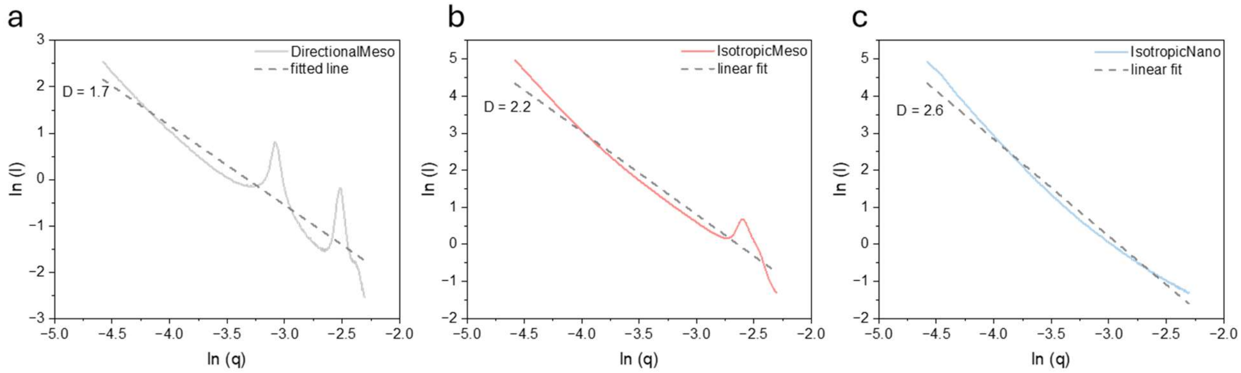

Supplementary Figure 15. Fractal analysis in SAXS on (a) DirectionalMeso, (b) IsotropicMeso, and (c) IsotropicNano.

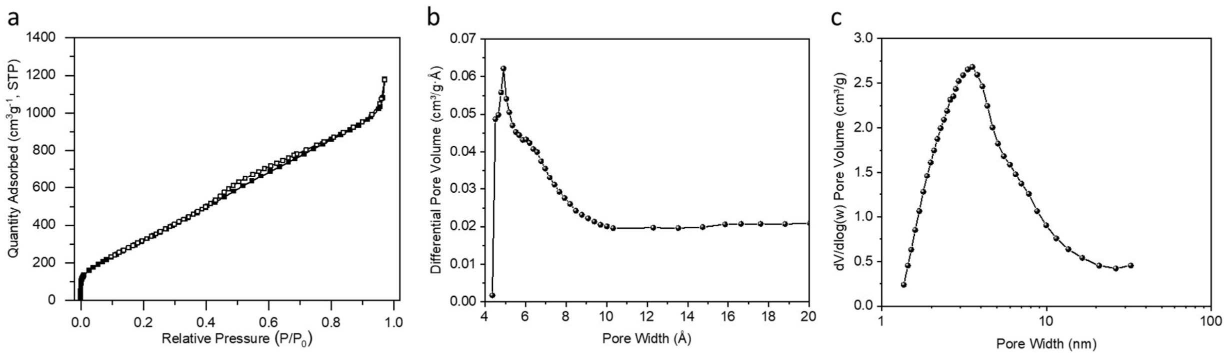

Supplementary Figure 16. (a) BET isothermal plot and (b)-(c) pore size distribution on DirectionalMeso with long channels. The nanopore size was determined using the Horvath-Kawazoe method, with measurements conducted on a Micromeritics Tristar instrument. The mesopore size was determined using the Horvath-Kawazoe method, with measurements taken on a Micromeritics 3Flex instrument.

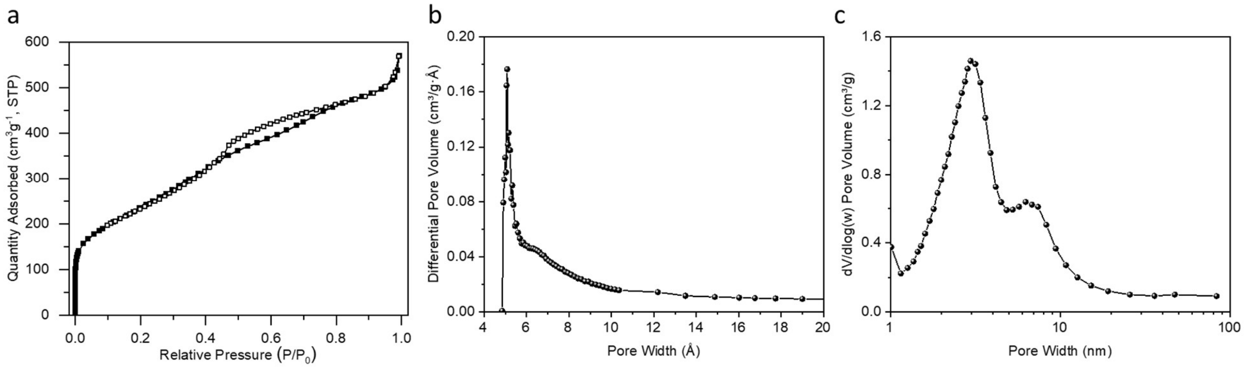

Supplementary Figure 17. (a) BET isothermal plot and (b)-(c) pore size distribution on DirectionalMeso with short channels. The nanopore size was determined using the Horvath-Kawazoe method, with measurements conducted on a Micromeritics Tristar instrument. The mesopore size was determined using the Horvath-Kawazoe method, with measurements taken on a Micromeritics 3Flex instrument.

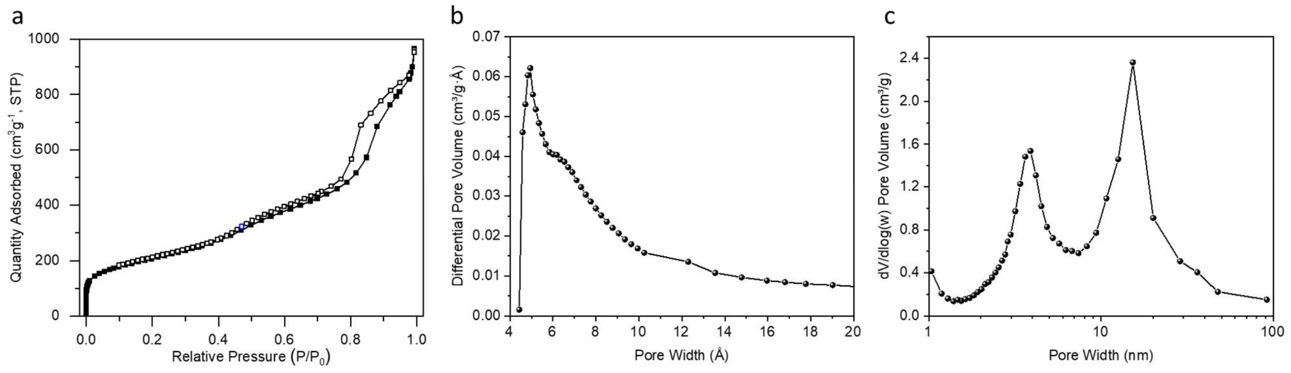

Supplementary Figure 18. (a) BET isothermal plot and (b)-(c) pore size distribution on IsotropicMeso. The nanopore size was determined using the Horvath-Kawazoe method, with measurements conducted on a Micromeritics Tristar instrument. The mesopore size was determined using the Horvath-Kawazoe method, with measurements taken on a Micromeritics 3Flex instrument.

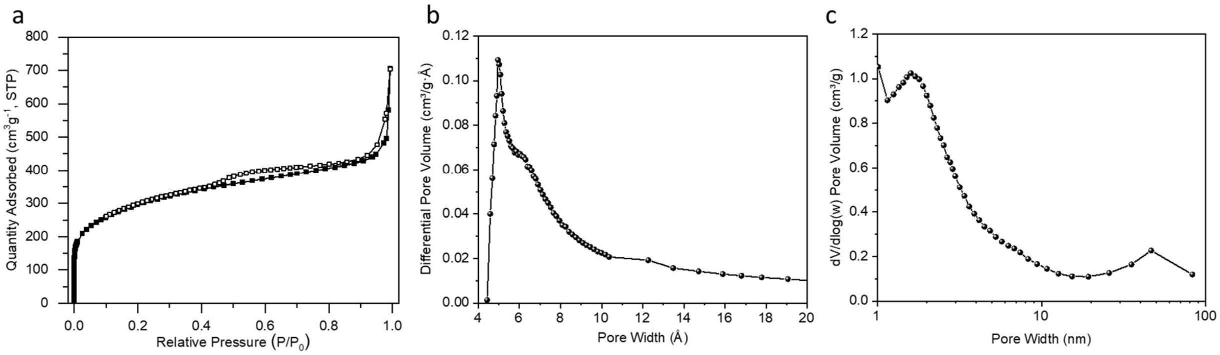

Supplementary Figure 19. (a) BET isothermal plot and (b)-(c) pore size distribution on IsotropicNano. The nanopore size was determined using the Horvath-Kawazoe method, with measurements conducted on a Micromeritics Tristar instrument. The mesopore size was determined using the Horvath-Kawazoe method, with measurements taken on a Micromeritics 3Flex instrument.

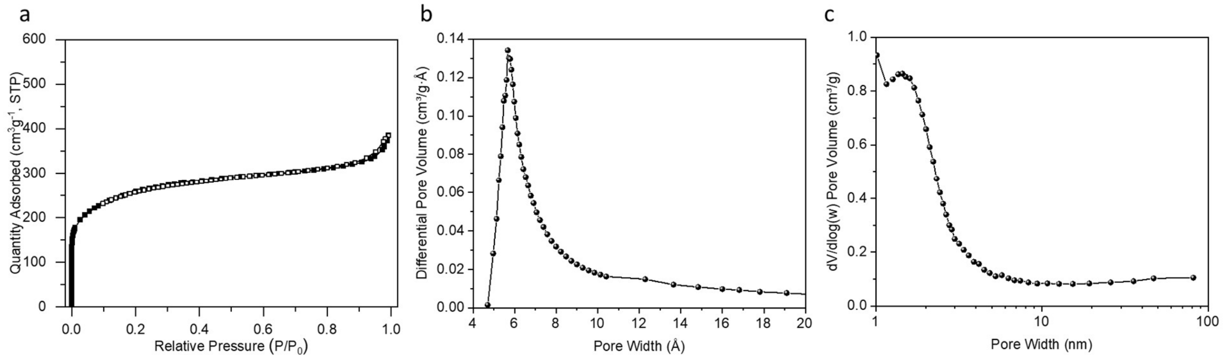

Supplementary Figure 20. (a) BET isothermal plot and (b)-(c) pore size distribution on IsotropicNano after NH_3 treatment. The nanopore size was determined using the Horvath-Kawazoe method, with measurements conducted on a Micromeritics Tristar instrument. The mesopore size was determined using the Horvath-Kawazoe method, with measurements taken on a Micromeritics 3Flex instrument.

Supplementary Table 3. Textural properties of the catalysts[c]

Catalyst	Surface area (m^2g^{-1})			Average pore width (nm) (4V/A by BET)
	S_{BET}	S_{micro}[a]	S_{meso}[a]	
DirectionalMeso-long channel	729.5	54.7	674.8	8.2
DirectionalMeso-short channel	818.7	-[b]	818.7	4.3
IsotropicMeso	729.5	54.7	674.8	8.2
IsotropicNano	1044.0	114.0	930	4.2
IsotropicNano-NH_3 treatment	920.9	253.7	667.2	2.6

a. The micropore and mesopore surface area was determined by the t-plot method.

b. Non-detectable.

c. All the results were measured on the instrument of Micromeritics 3Flex.

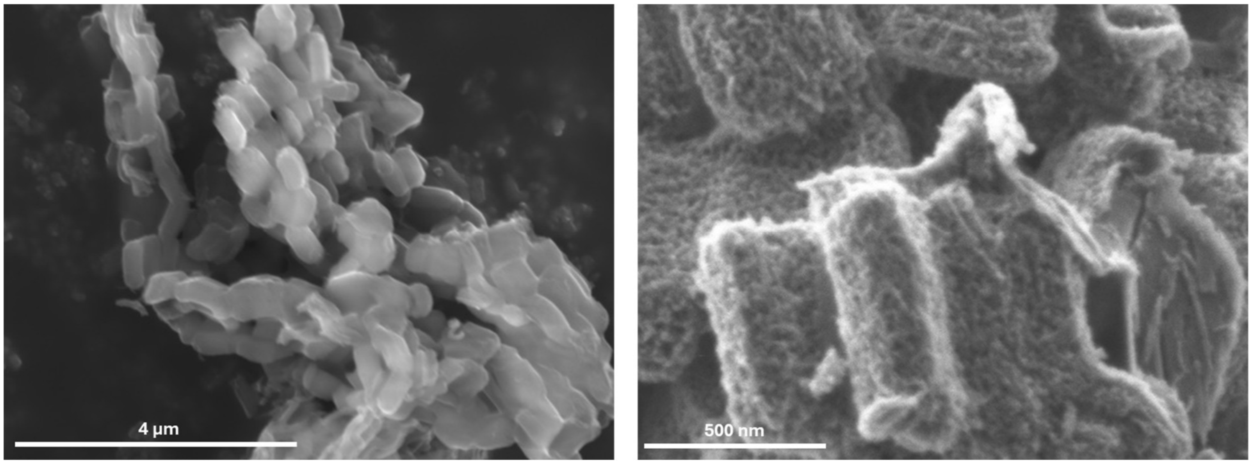

Supplementary Figure 21. SEM images of DirectionalMeso.

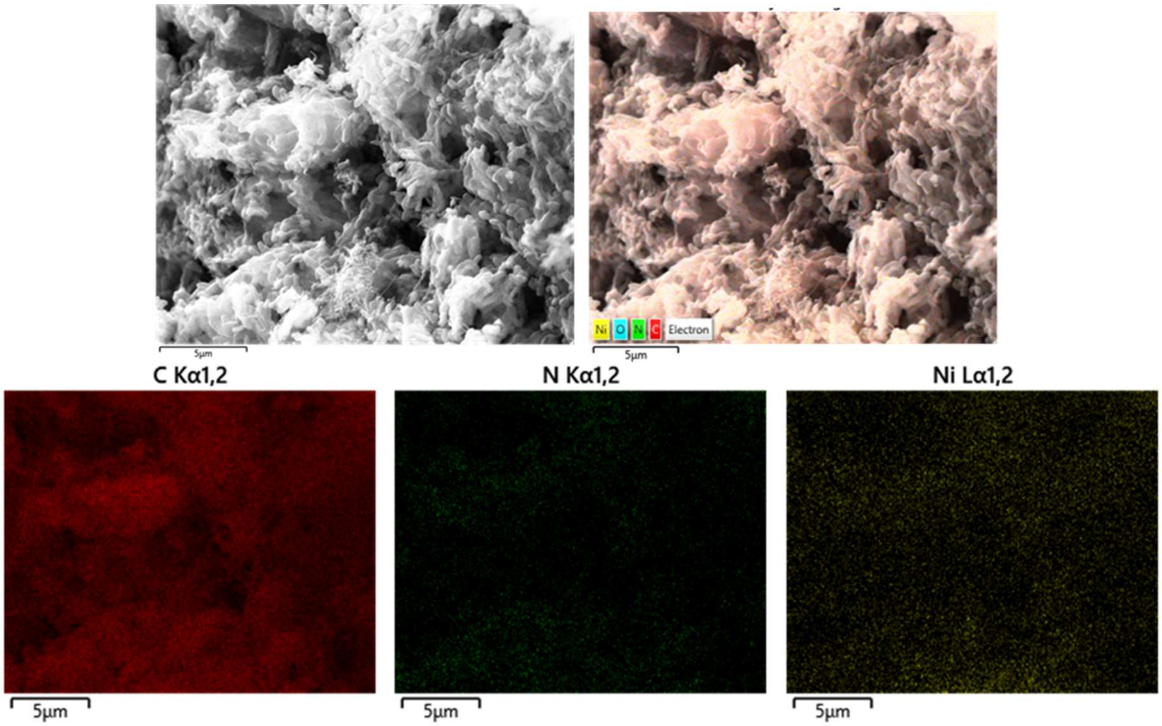

Supplementary Figure 22. SEM-EDS images of DirectionalMeso.

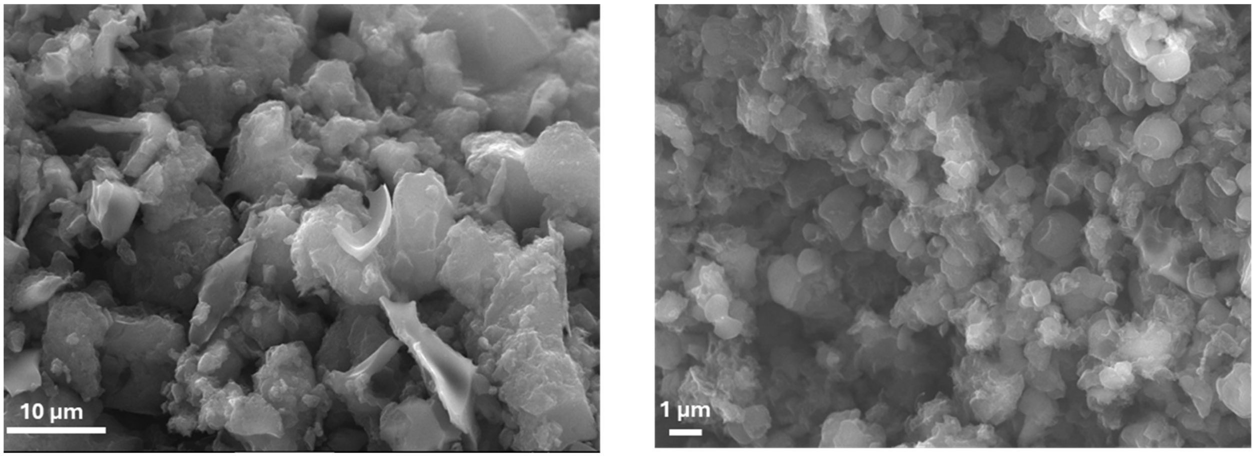

Supplementary Figure 23. SEM images of IsotropicMeso.

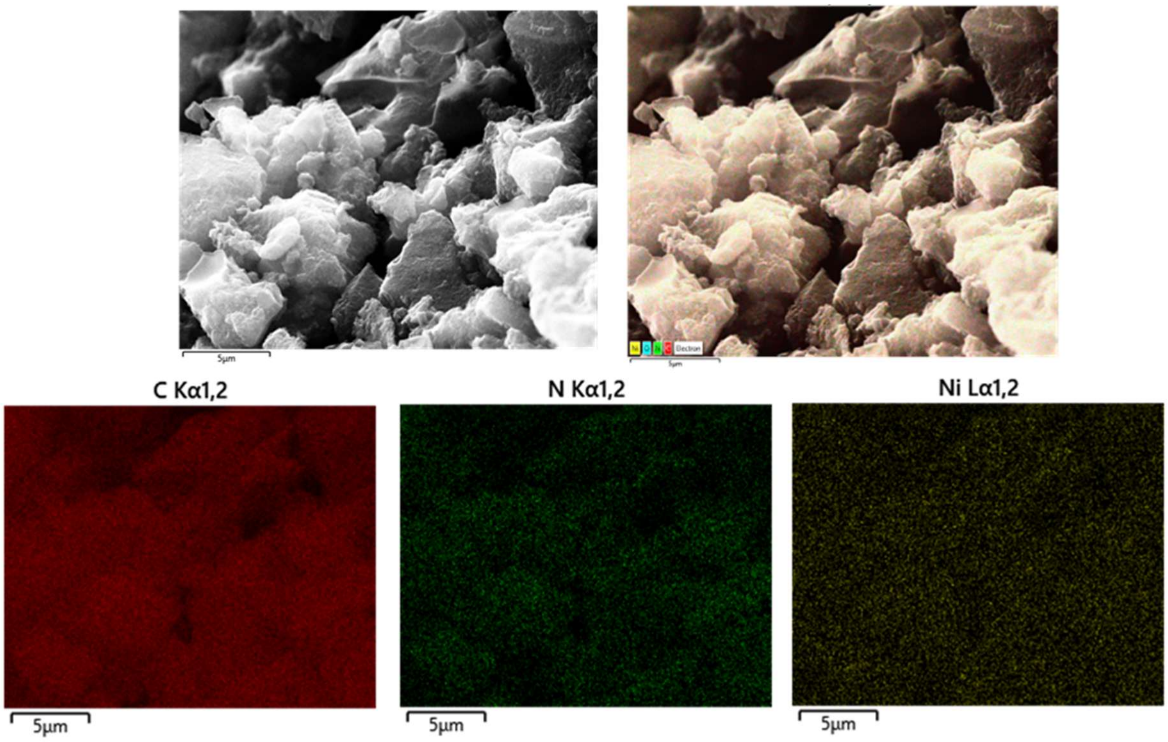

Supplementary Figure 24. SEM-EDS images of IsotropicMeso.

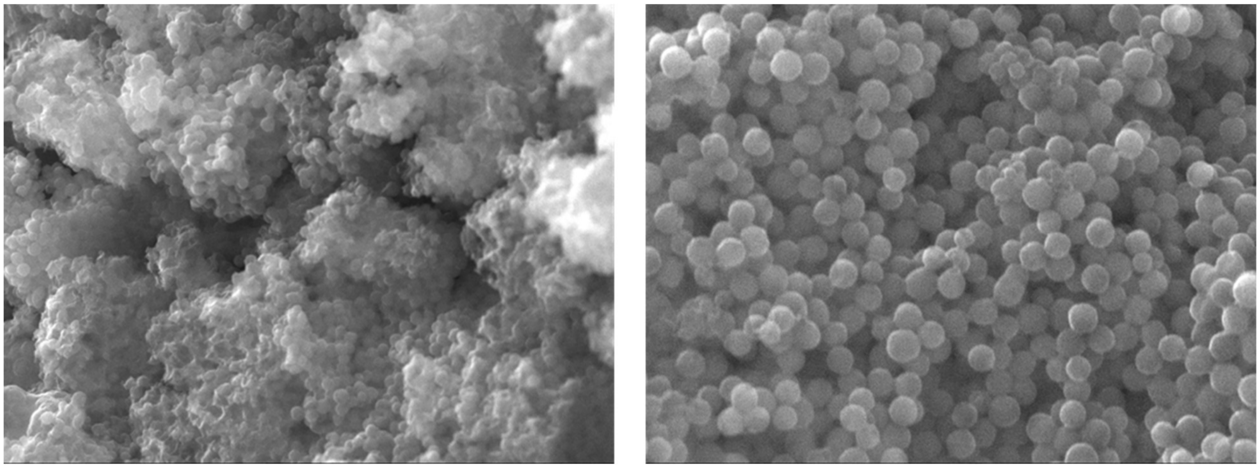

Supplementary Figure 25. SEM images of IsotropicNano.

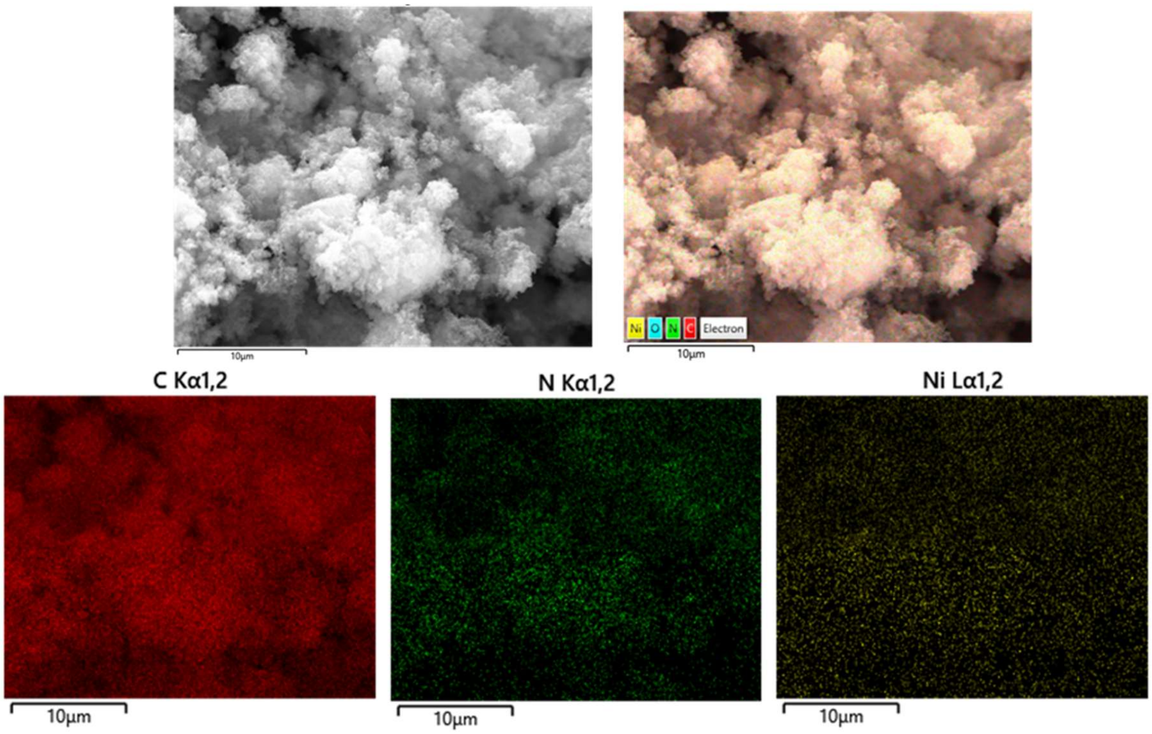

Supplementary Figure 26. SEM-EDS images of IsotropicNano.

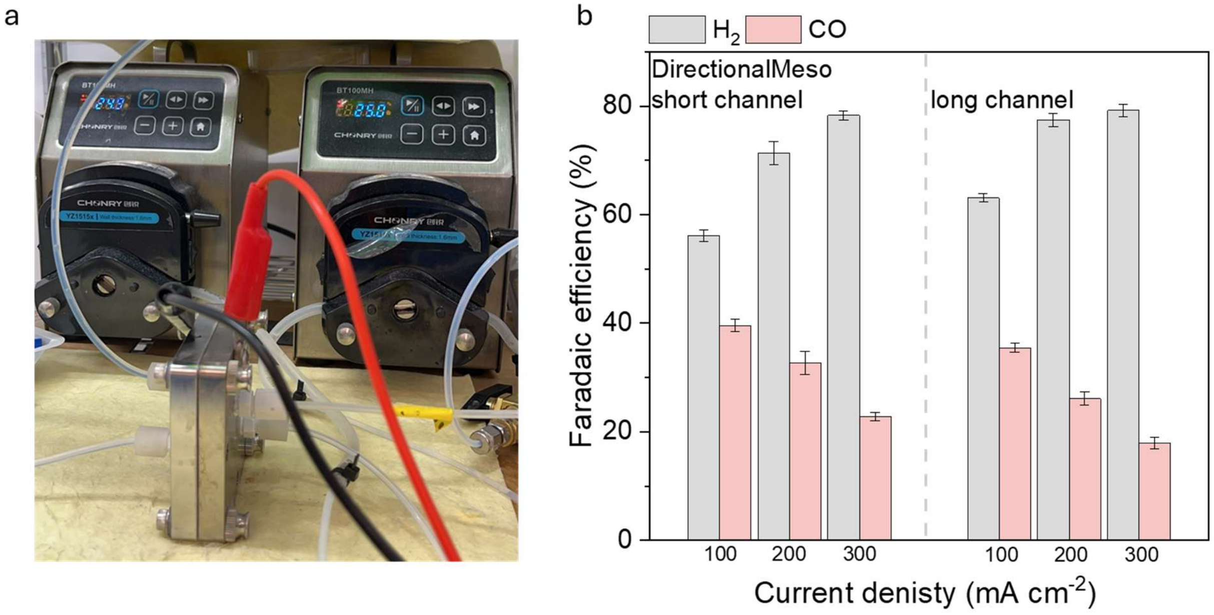

Supplementary Figure 27. (a) Photograph of the BPM-based carbonate electrolyzer. (b) Faradaic efficiency of CO and H_2 on DirectionalMeso with short and long channels.

Supplementary Table 4. Distribution and utilization of *i*-CO_2 in carbonate-fed electrolysis on various catalysts.

Catalyst	Current desnity (mA cm^{-2})	total *i*-CO_2 production rate (mmole cm^{-1} s^{-1})	CO production rate (mmole cm^{-1} s^{-1})	gas CO_2 at cathode outlet (mmole cm^{-1} s^{-1})	*i*-CO_2-OH^- coupling rate (mmole cm^{-1} s^{-1})	CO FE (%)
DirectionalMeso	200	3.73	1.22	0.09	2.42	32.7
	300	5.60	1.28	0.12	4.20	22.8
IsotropicMeso	200	3.73	1.26	0.07	2.40	33.9
	300	5.60	1.61	0.11	3.88	28.8
IsotropicNano	200	3.73	1.75	0.03	1.96	46.8
	300	5.60	2.80	0.05	2.75	50.1

Supplementary Table 5. Summary of ICP-MS results.

Catalyst	Ni content (wt.%)
DirectionalMeso-long channel	1.67
DirectionalMeso-short channel	1.80
IsotropicMeso	1.50
IsotropicNano	1.35

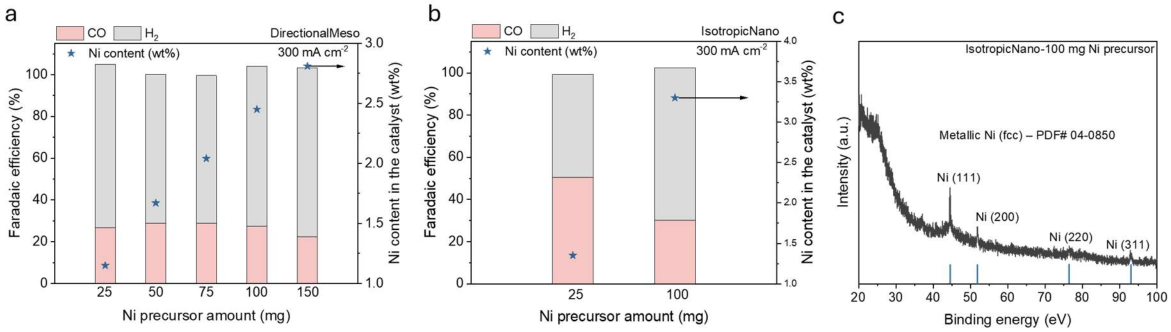

Supplementary Figure 28. Ni loading study in carbonate-fed-electrolyzer. The FE to product on (a) DirectionalMeso and (b) IsotropicNano with various Ni precursor amount at 300 mA/cm^2. (c) XRD on IsotropicNano with 100 mg of Ni precursor. The diffraction peaks at ~44.5°, 51.8°, and 76.4° (corresponding to the (111), (200), and (220) planes of fcc Ni, PDF# 04-0850) corresponding to metallic Ni nanoparticles

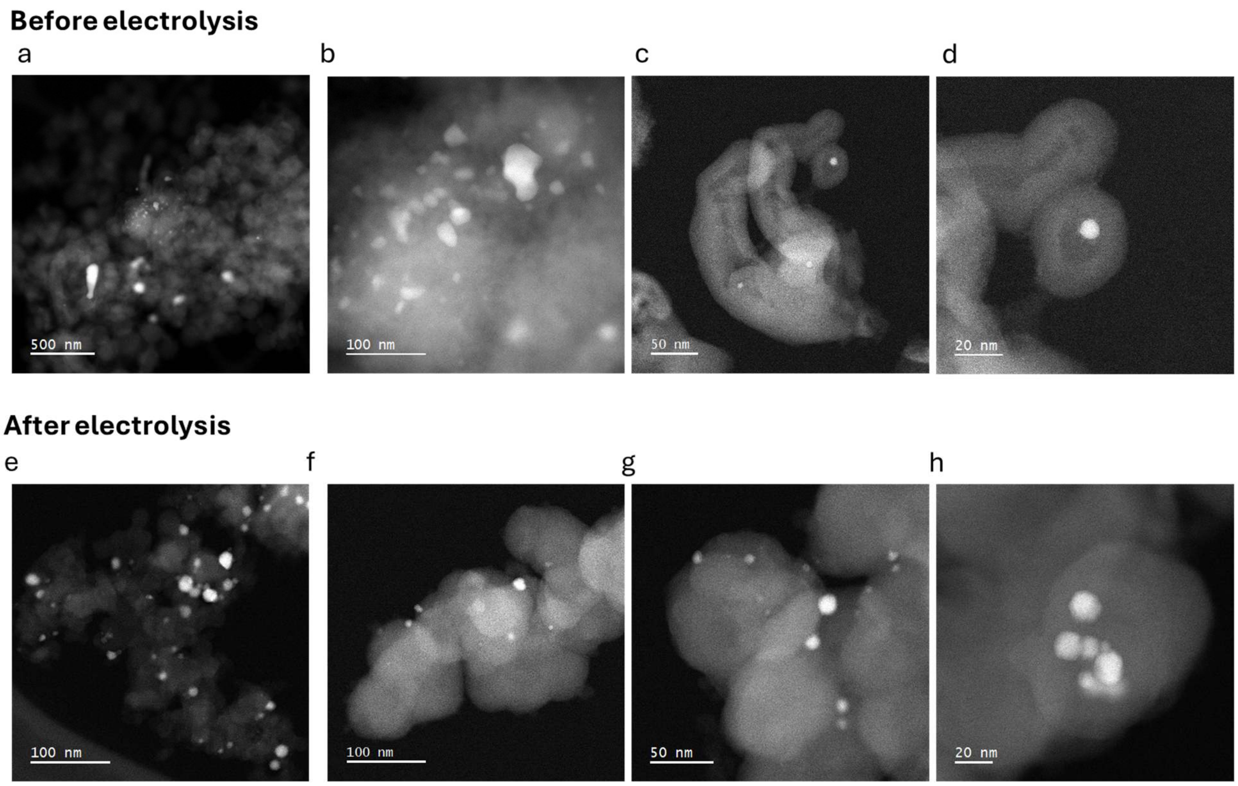

Supplementary Figure 29. HRTEM images of high-Ni-loading samples (3.3 wt% Ni): (a–d) before electrolysis and (e–f) after electrolysis. The electrolysis was performed at 300 mA cm^{-2} for 4 hours. The high loading IsotropicNano was synthesized with 100 mg Ni precursor.

Supplementary Note 3. Comparing gas-fed and liquid-fed CO_2RR with porous single-atom vs. non-porous nanoparticle catalysts

We have conducted additional control experiments to investigate the differences in gas-fed CO_2RR vs. liquid-fed carbonate systems, as well as porous single-atom catalysts (Ni-SAC) vs. non-porous nanoparticle catalysts (Ag).

(1) Gas-fed CO_2RR vs. liquid-fed carbonate system

The fundamental difference between gas-fed CO_2RR and liquid-fed carbonate electrolysis lies in reactant availability and catalyst environment. Gas-fed systems benefit from a hydrophobic gas-diffusion layer that ensures a sufficient CO_2 supply, whereas liquid-fed carbonate systems operate in a hydrophilic, fully flooded environment where *i*-CO_2 (g) (aq) (in both gas and liquid form) is limited and must be efficiently utilized.

To investigate transport effects, we conducted control experiments in a MEA-based gas-fed CO_2RR system using IsotropicNano (**Supplementary Fig. 30a-b**).

When pure CO_2 gas was supplied, the FE_{CO} remained above 95% at 200 mA/cm^2 across a catalyst loading range of 0.5–2 mg/cm^2 (**Supplementary Fig. 30a**). When CO_2 partial pressure was reduced to 20% (**Supplementary Fig. 30b**), the catalyst loading effect became evident, and loading below 1 mg/cm^2 led to a 15–20% decrease in FE_{CO}.

In contrast, when switching to the carbonate system at the same current density (200 mA/cm^2), the catalyst loading effect became much more pronounced (**Supplementary Fig. 30c**). Reducing the catalyst loading from the optimal 2 mg/cm^2 to 0.5 mg/cm^2 resulted in a 50% drop in FE_{CO}.

This suggests that in a fully flooded environment, not only the top catalyst layer participates in the reaction. Instead, *i*-CO_2 (g) (aq) produced at the BPM surface must penetrate through the full catalyst layer for conversion, making pore structure and transport properties crucial for managing the utilization of the limited *i*-CO_2 supply.

(2) Porous single-atom catalysts vs. non-porous nanoparticles

IsotropicNano (porous) and Ag nanoparticles (non-porous) in the carbonate-fed system exhibited distinct behavior as a function of catalyst loading (**Supplementary Fig. 30c-d**).

IsotropicNano exhibited a stronger loading dependence, as it consists of only ~1.3 wt% Ni distributed within a porous carbon-nitrogen matrix. The lower active site density amplifies the impact of catalyst loading. In contrast, Ag nanoparticles, which are densely packed with abundant active sites, showed a less significant loading effect (**Supplementary Fig. 30d**).

These results indicate that porous catalysts with highly dispersed active sites require optimized loading to ensure effective CO_2 utilization within the electrode structure.

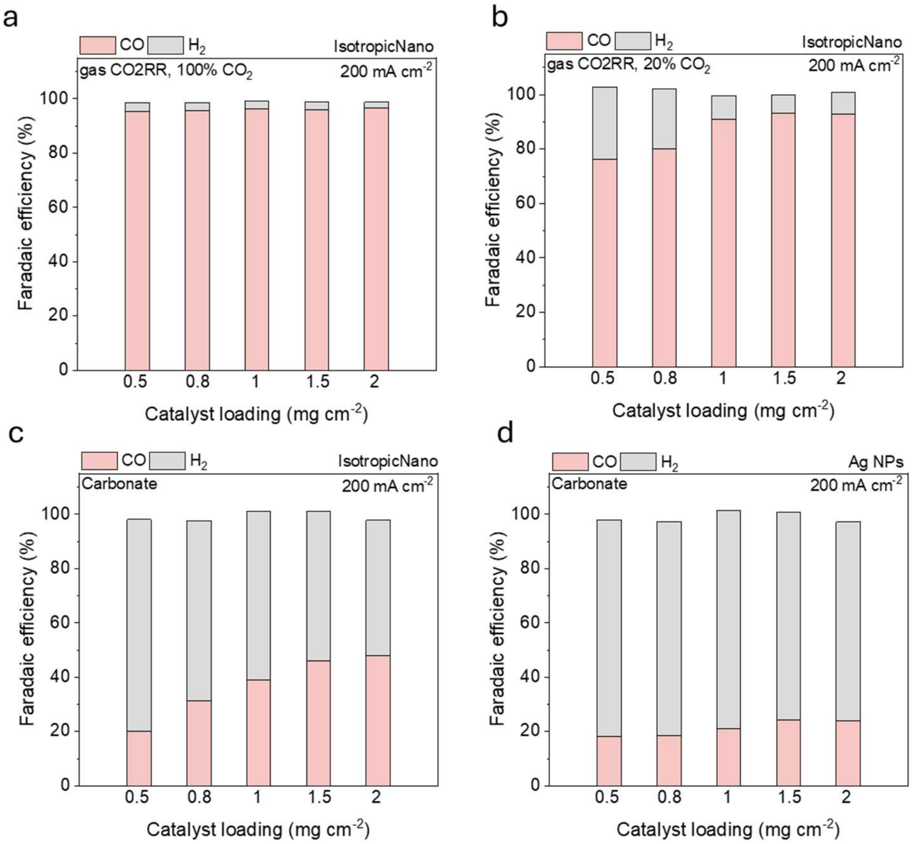

Supplementary Figure 30. Catalyst loading studies in gas-fed and liquid-fed CO_2 systems using IsotropicNano and Ag NP catalysts. (a, b) Faradaic efficiency to products in a MEA-based electrolyzer with (a) pure CO_2 gas feed and (b) 20% CO_2 gas feed on IsotropicNano at various catalyst loadings. Faradaic efficiency to products in a carbonate-fed electrolyzer using (c) IsotropicNano and (d) Ag NPs at varying catalyst loadings. All electrolysis was performed at 200 mA/cm^2.

(3) Catalyst thickness effect

To eliminate catalyst thickness as a variable, we coated a constant total catalyst + carbon loading onto the electrode while varying the Ni-SAC: carbon ratio (from 3:1 to 1:3) for carbonate electrolysis (**Supplementary Figure 30**).

Even with similar overall electrode thicknesses, we found that the Ni-SAC layer thickness and content remained critical in determining FE_{CO}. This indicates that pore structure and active site accessibility throughout the entire optimized catalyst layer play a dominant role, rather than CO_2 reduction occurring only at the top surface in the carbonate system with limited CO_2 (g) (aq) supply.

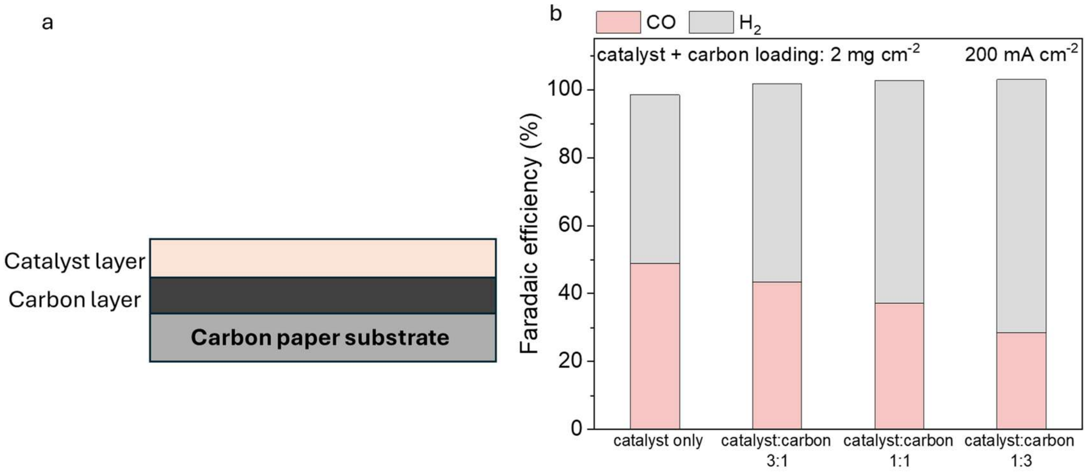

Supplementary Figure 31. Effect of IsotropicNano layer thickness on carbonate-fed-electrolyzer performance. (a) Schematic illustration of the catalyst and carbon layers on a carbon paper substrate. (b) Faradaic efficiency to products with a constant total loading of 2 mg/cm^2 (IsotropicNano + carbon), varying the catalyst-to-carbon ratio.

(4) Effect of high flow rate on local OH^- buffering

Another key distinction between gas-fed and liquid-fed systems is the local buffering effect due to high electrolyte flow rates. In our carbonate system, we used a high liquid flow rate (50 mL/min) to maintain a relatively uniform local pH during electrolysis.

Whether using pure K_2CO_3 or a $K_2CO_3/KHCO_3$ buffer system, a high circulation rate helped stabilize the local pH by mitigating OH^- accumulation and enhancing CO_2 availability. This partially suppressed OH^-–CO_2 coupling reactions that would otherwise deplete *i*-CO_2.

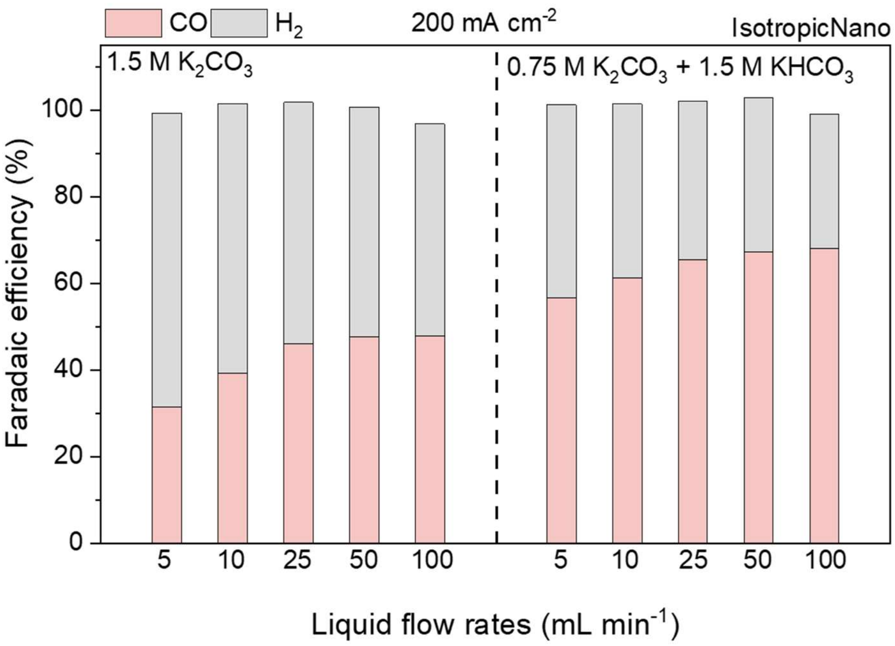

Supplementary Figure 32. Effect of liquid flow rate in carbonate-fed-electrolyzer. Faradaic efficiency to products with various liquid flow rates in 1. 5 M K_2CO_3 and 0.75 M K_2CO_3 + 1.5 M $KHCO_3$ electrolytes on IsotropicNano catalyst at 200 mA/cm^2.

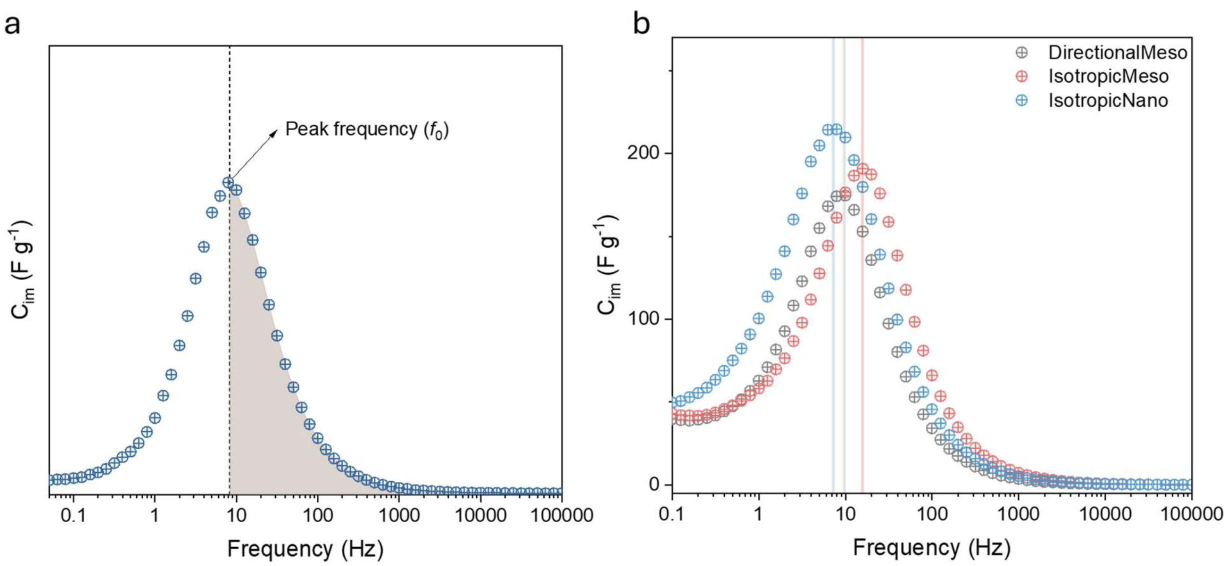

Supplementary Figure 33. Imaginary part of complex capacitance (C_{im}) obtained by complex capacitance analysis through EIS method. (a) Illustration of the peak area (A) calculation by doubling the integrated area of the right-hand side of the curve (shaded region). (b) C_{im} as a function of frequency for the three catalysts.

Electrochemical impedance spectroscopy (EIS) was utilized to investigate the dynamic behavior of porous catalysts under non-Faradaic conditions. Key parameters, including the total capacitance (C_{dl}) and the relaxation time constant (τ_o), were derived from the EIS response using complex capacitance analysis. The relationship of $Z(\omega)$ and complex capacitance C(ω) are expressed as follows:

$$Z(\omega) = \frac{1}{j\,\omega\,C(\omega)}$$

$$C(\omega) = C'(\omega) - j\,C''(\omega)$$

$$C' = \frac{-Z''(\omega)}{\omega|Z(\omega)|^2}, \qquad C'' = \frac{Z'(\omega)}{\omega|Z(\omega)|^2}$$

Where $Z(\omega)$ is impedance and ω is frequency. $C'(\omega)$ is the real part of the complex capacitance reflecting frequency-dependent capacitance, and $C''(\omega)$ is the imaginary part related to energy dissipation from processes like IR drop and irreversible Faradaic charge transfer.

From the peak frequency of $C''(\omega)$, the relaxation time constant (τ_o) is calculated as below.

$$\tau_o = (2\pi f_o)^{-1}$$

where is the frequency corresponding to the peak in $C''(\omega)$. The relaxation time constant provides insight into the electrode's pore structure and electrochemical behavior.

For capacitance estimation, the total static capacitance (C_{dl}) was assessed by analyzing the integrated area under the $C''(\omega)$ curves. Based on the proportional relationship dictated by Kramers-Kronig relations:

$$A = -\int_{-\infty}^{\infty} C''(\omega) d \log f = 0.682\, C_{dl}$$

where A is the peak area under $C''(\omega)$ curves. Given challenges in measuring at very low frequencies, A was approximated by doubling the integrated area of the right-hand side of the curve (the shadowed part in **Supplementary Figure 33**):

$$C_{dl} = -\frac{2}{0.682} A_{right-half}, \qquad A_{right-half} = \int_{f_o}^{10^5} C''(\omega)\, d \log f$$

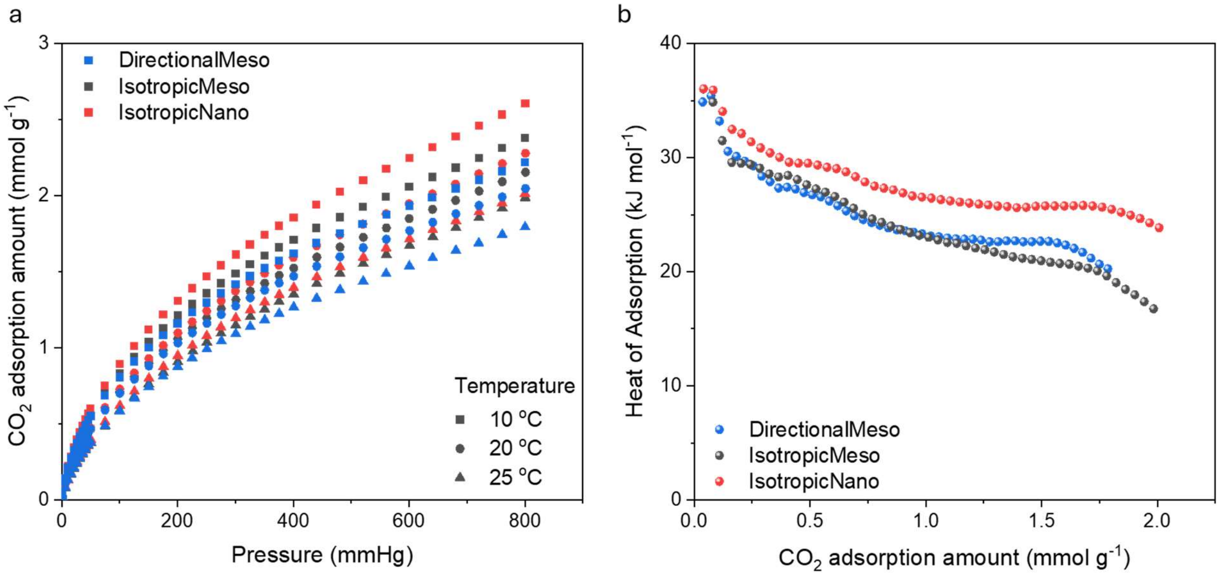

Supplementary Figure 34. CO_2 adsorption isothermal analysis. (a) CO_2 adsorption isotherms of three catalysts at 10, 20, and 25 ºC. (b) Isosteric heat of adsorption for CO_2 on three catalysts.

\

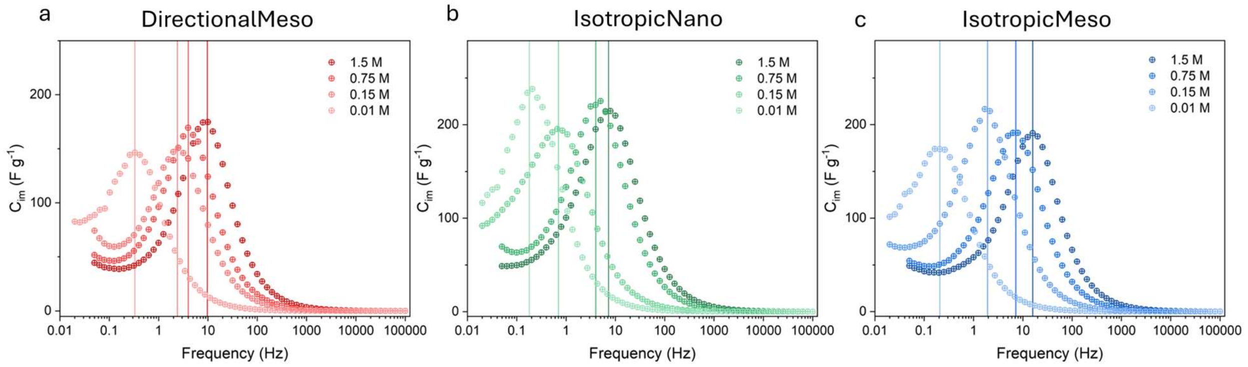

Supplementary Figure 35. (a) – (c) Imaginary capacitance plots of three catalysts in different concentration of K_2CO_3 solution.

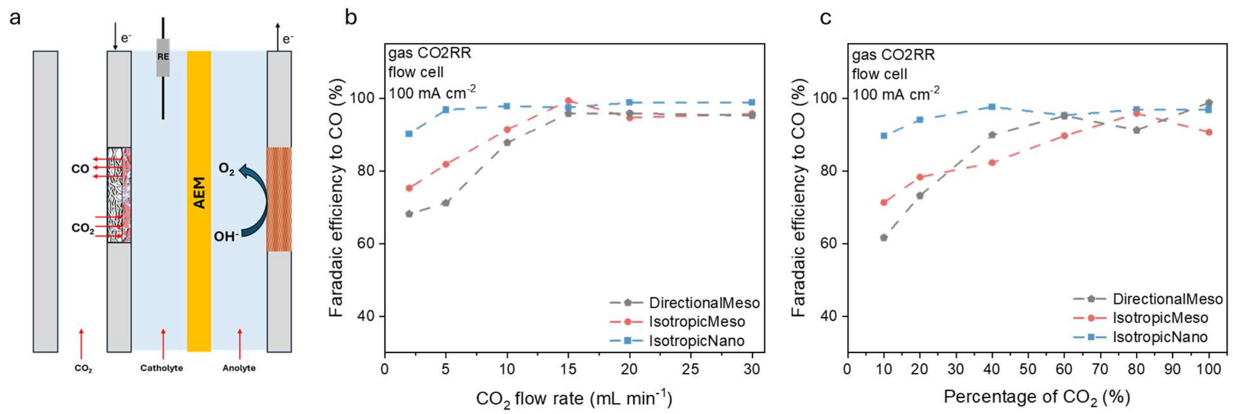

Supplementary Figure 36. Gas CO_2 electrolysis in flow cell. (a) Schematic illustration of flow cell set-up for gas CO_2 reduction. (b) – (c) Faradaic efficiency to CO as a function of CO_2 flow rate and CO_2 partial pressure at 100 mA/cm^2.

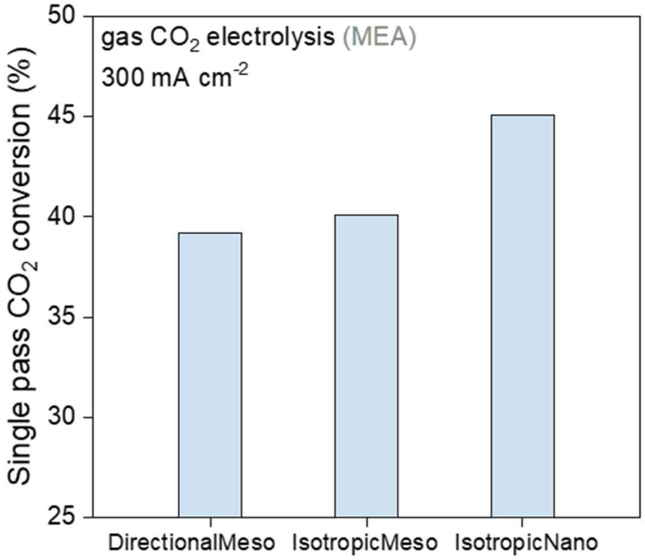

Supplementary Figure 37. Gas CO_2 electrolysis in MEA electrolyzer. Single-pass conversion of CO_2 in gas-CO_2-fed, MEA-based electrolyzer. An AEM was used as the ion exchange membrane in the gas-CO_2-fed studies.

In MEA-based electrolyzers for gas-fed CO_2 reduction, single-pass (SP) efficiency serves as a diagnostic tool to analyze mass transport limitations and catalyst performance.[13] This metric provides insights into the catalyst layer's efficiency in utilizing locally supplied CO_2, particularly under electrolysis conditions where CO_2 is catalytically consumed (e.g., CO_2-to-CO conversion). This behavior closely parallels that of carbonate electrolysis systems with ultra-low CO_2 supply rates.

Catalytic Single Pass Efficiency (SP): $\mathrm{SP} = \frac{\dot{V}_{CO}}{\dot{V}_{CO2,in}} \times 100\%$

Where $\dot{V}_{CO}$ and $\dot{V}_{CO2,in}$ represent the volume flow rates of the produced CO and the total CO_2 inlet, respectively. SP represents the ratio of CO outlet flow to total CO_2 input flow. A high SP indicates efficient CO_2 transfer and catalytic conversion.

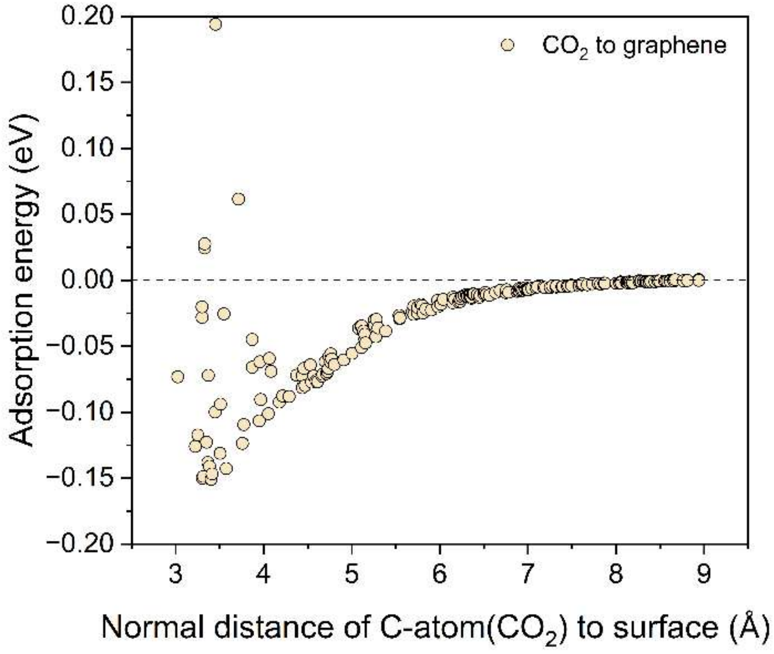

Supplementary Figure 38. The CO_2 adsorption energy as a function of the distance normal to the graphene surface. They were calculated using density functional theory (DFT) computational approach. A total of 200 cases were considered, accounting for random rotations and distributions of CO_2 in the parallel plane at each specified normal distance.

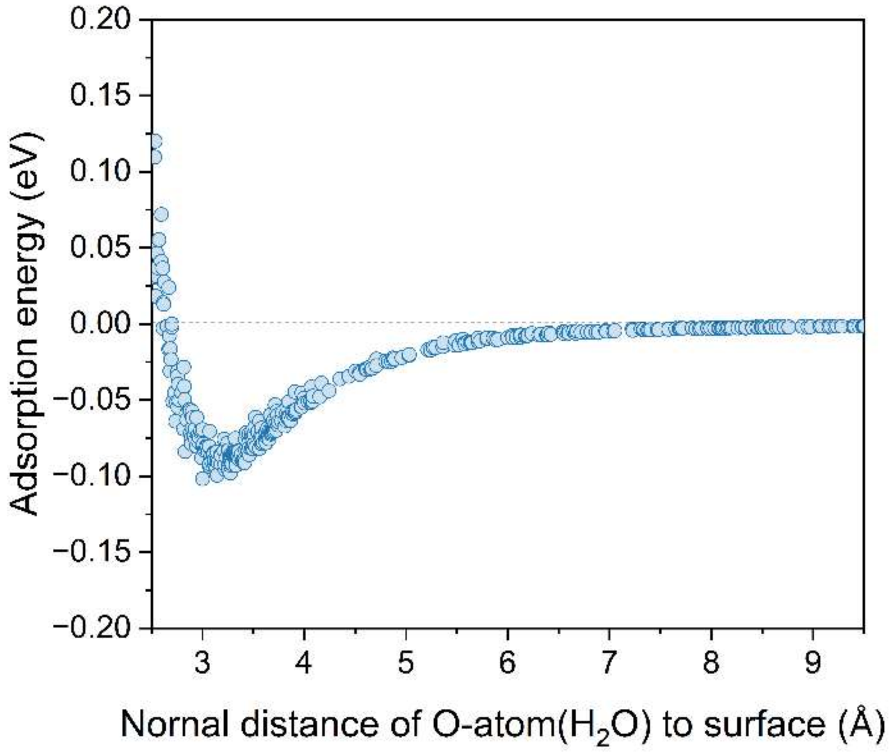

Supplementary Figure 39. The H_2O adsorption energy as a function of the distance normal to the Ni-SAC surface. They were calculated using density functional theory (DFT) computational approach. A total of 400 DFT cases were considered, accounting for H_2O's random rotations and distributions in the parallel plane at each specified normal distance.

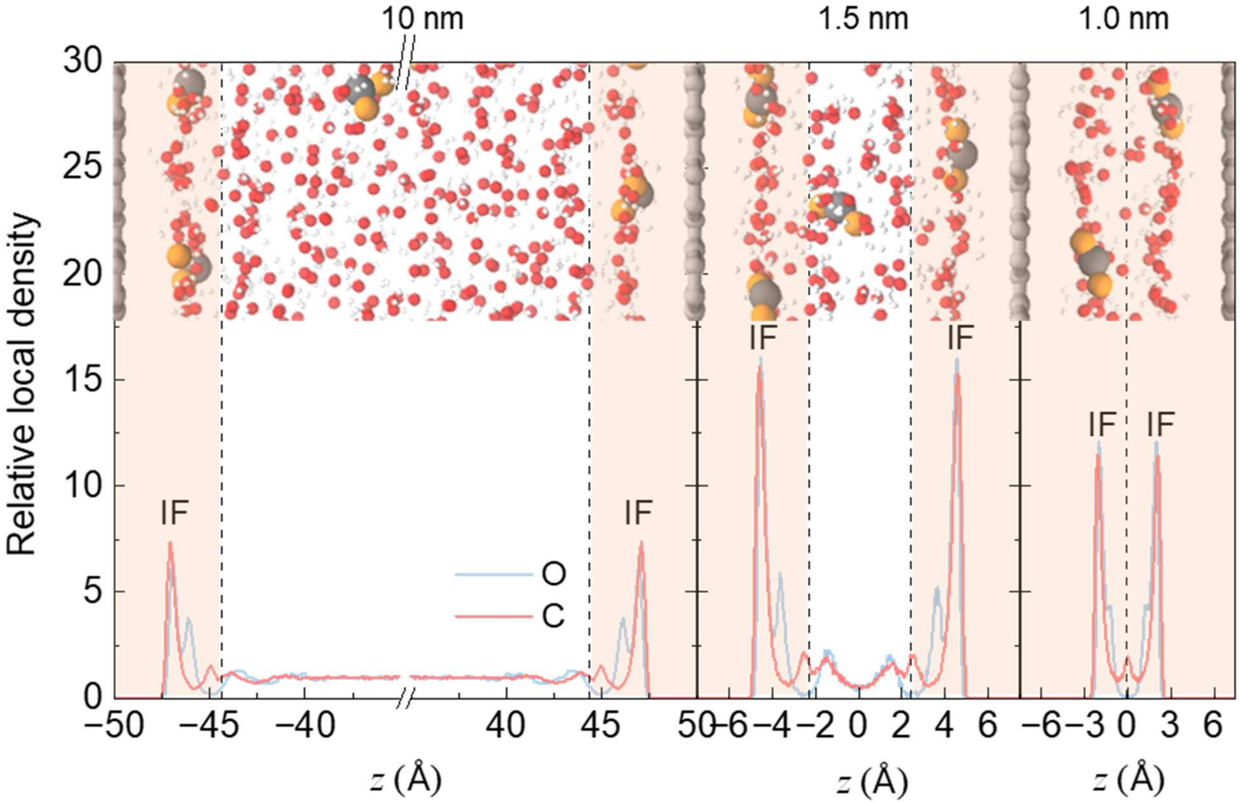

Supplementary Figure 40. The re-plot of Figure 3c. It shows the local relative density distribution of CO_2 in the normal direction to the pore surface, comparing three slit sizes: 1.0 nm, 1.5 nm, and 10 nm. To better illustrate the changes in the occupying volume ratio of the interfacial region as a function of pore size, the interfacial region has been specifically highlighted in the plots for clarity.

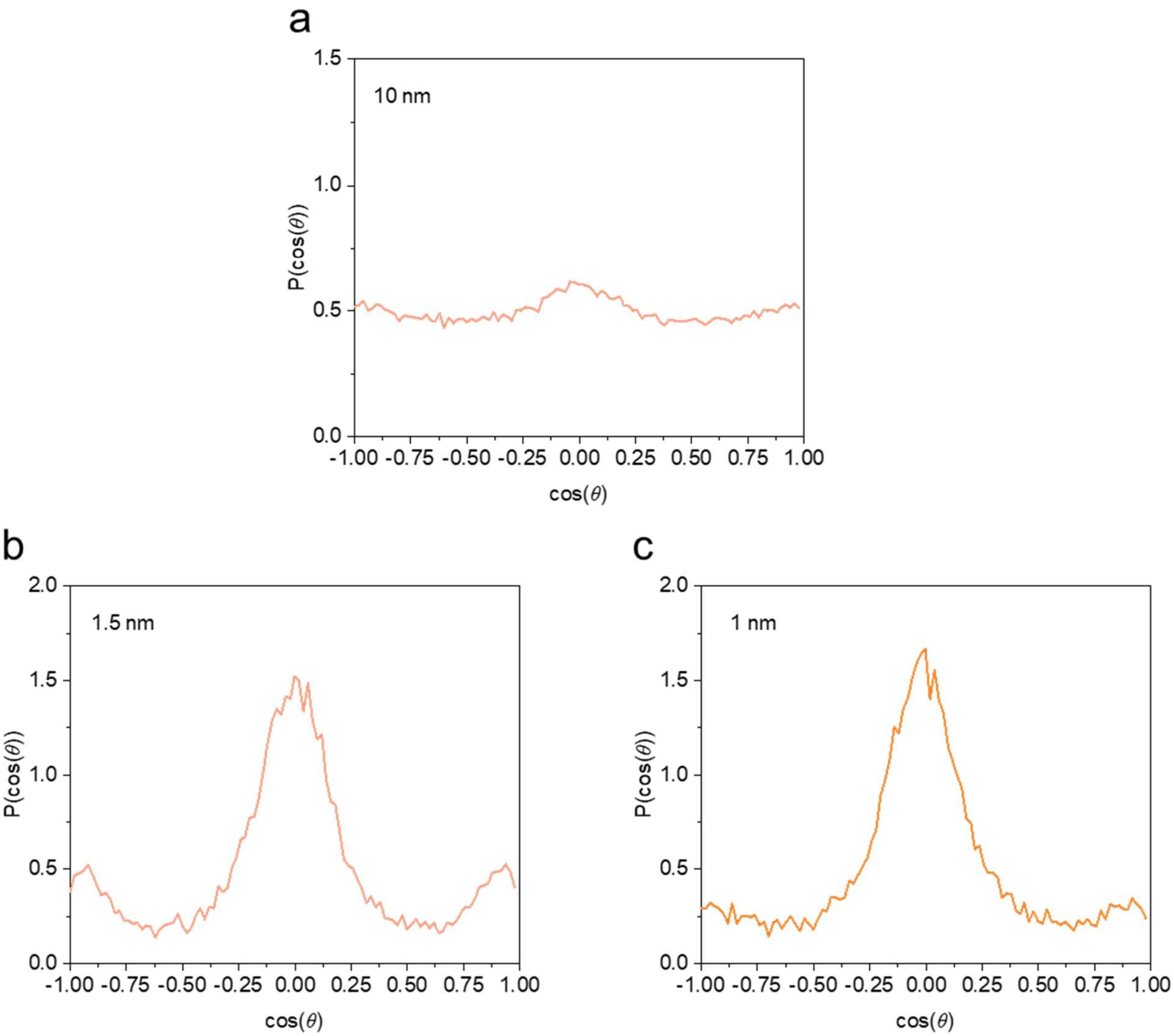

Supplementary Figure 41. The orientation probability distribution of CO_2 within the slit. Three slit sizes are considered, including 10 nm (a), 1.5 nm (b), and 1.0 nm (c). The angle θ is defined as the direction of C-O bond relative to the normal plane of the catalyst surface. Note that as slit size decreases, there is increased peak at $\cos\theta = 0$, corresponding more aligned distribution of CO_2 in slits (e.g., more CO_2 molecules are parallel to surface).

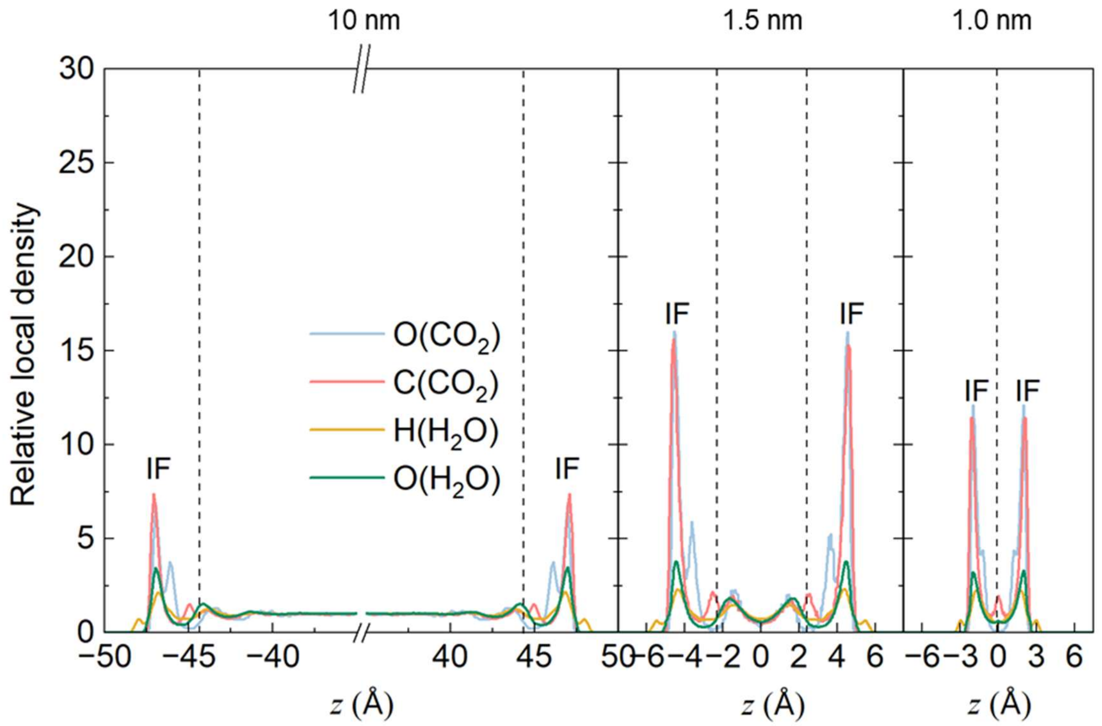

Supplementary Figure 42. The local relative density distribution of CO_2 and H_2O molecules in the normal direction to the pore surface. Three pore sizes are considered, including the 1.0 nm, 1.5 nm, and 10 nm.

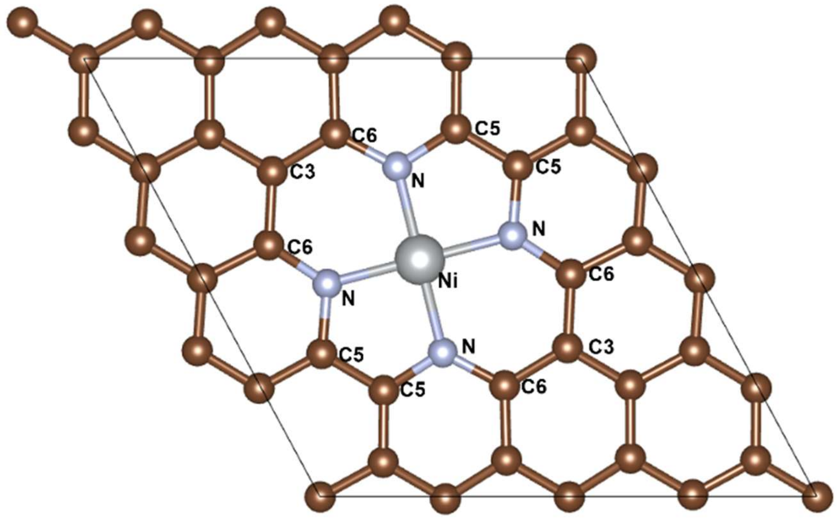

Supplementary Figure 43. Schematics of atom type in Ni-SAC used in our MD simulations.

Supplementary Table 6. Partial charges of each atom type considered in MD simulations.

Atoms	Valence (*e*)
C3 (Ni-SAC)	–0.15
C5 (Ni-SAC)	0.0925
C6 (Ni-SAC)	0.18
CP (Ni-SAC)	0
N (Ni-SAC)	–0.28
Ni (Ni-SAC)	0.33
O (H_2O)	–0.76
H (H2O)	0.38
C (CO_2)	0.70
O (CO_2)	–0.35

Note: For detailed schematics illustrating the location of each atom type on the materials, please refer to **Supplementary Figure 43.**

Supplementary Table 7. Lennard-Jones parameters of each atom type considered in MD simulations.

Atoms	ε (Kcal mol^{-1})	σ (Å)
C3 (Ni-SAC)	3.55	0.070104
C5 (Ni-SAC)	3.55	0.070104
C6 (Ni-SAC)	3.55	0.070104
CP (Ni-SAC)	3.55	0.070104
N (Ni-SAC)	3.26	0.068999
Ni (Ni-SAC)	2.52	0.014999
O (H_2O)	3.1506	0.152103
H (H2O)	0.00	0.00000
C (CO_2)	2.757	0.055898
O (CO_2)	3.033	0.159994
C(CO_2)-C3/C5/C6/CP	2.8153	0.088890
C(CO_2)-N	2.3070	0.159485
C(CO_2)-Ni	2.9576	0.092796
O(CO_2)-C3/C5/C6/CP	3.0418	0.152474
O(CO_2)-N	2.542567	0.103318
O(CO_2)-Ni	3.30302	0.109139
O(H_2O)-C3/C5/C6/CP	3.76280	0.018940
O(H_2O)-N	2.69311	2.165238
O(H_2O)-Ni	2.6975	1.467798
H(H_2O)-C3/C5/C6/CP	1.9502	0.321363
H(H_2O)-N	2.1561	0.079234
H(H_2O)-Ni	3.3493	0.194864

Supplementary Note 5. Electrokinetic study

(1) CO_2 binding affinity

Electrokinetic analysis provides insights into whether the reaction is kinetically controlled (limited by charge transfer) or diffusion-controlled (limited by reactant transport).

To simulate the reactive capture system, we conducted electrolysis using varying concentrations of gaseous CO_2 in K_2CO_3 solutions. The experiments were performed in three-electrode flow cells, with porous Ni single atom catalysts as the working electrode, Ni foam as the counter electrode, and Ag/AgCl as the reference electrode. CO_2 was diluted with Ar to achieve different concentrations, while maintaining a total gas flow rate of 20 mL min^{-1}.

The well-defined Ni single atoms interact directly with i-CO_2 in a stoichiometric manner, establishing a (quasi-)equilibrium with a distinct intermediate. To describe this process, we developed a kinetic model based on Michaelis-Menten kinetics, a framework commonly used for enzymatic catalysis.[14,15] We simplified the reaction pathway into the following two steps:

$$\mathrm{Ni} + \mathrm{CO_2} + \mathrm{e^-} \underset{k_\mathrm{d}}{\overset{k_\mathrm{a}}{\rightleftharpoons}} \mathrm{Ni} - \mathrm{CO_2^{\cdot-}}$$

$$\text{Ni-}\mathrm{CO_2^{\cdot-}} \overset{k_\mathrm{cat}}{\rightarrow} \mathrm{Ni} + \mathrm{CO}$$

where k_a, k_d, and k_cat represent the rate constants for CO_2 adsorption, desorption, and the catalytic reaction, respectively. The model considers Ni single sites binding to CO_2 to form an intermediate complex, Ni–$CO_2^{\cdot-}$, which subsequently releases CO as the product, regenerating the Ni single sites. Under conditions where the concentration of Ni single sites is significantly lower than that of CO_2, a steady state is rapidly established, ensuring that the concentration of the Ni–$CO_2^{\cdot-}$complex remains nearly constant over the timescale of product formation. Consequently, the rate of CO production (ν_CO) is then proportional to the concentration of the Ni–$CO_2^{\cdot-}$complex:

$$\text{Rate equation: } \nu_\mathrm{CO} = k_\mathrm{cat}\ \text{Ni-}\mathrm{CO_2^{\cdot-}}$$

The total catalyst concentration ($[\mathrm{Ni}]_0$) is the sum of the free catalyst concentration ([Ni]) and the [Ni-$CO_2^{\cdot-}$]complex:

$$[\mathrm{Ni}]_0 = [\mathrm{Ni}] + [\text{Ni-}\mathrm{CO_2^{\cdot-}}]$$

Under steady state, where $\frac{\mathrm{d}[\mathrm{Ni-CO_2^{\cdot-}}]}{\mathrm{dt}} = 0$

$$k_\mathrm{a}[\mathrm{CO_2}][\mathrm{Ni}] = k_\mathrm{a}[\mathrm{CO_2}]([\mathrm{Ni}]_0 - [\mathrm{Ni} - \mathrm{CO_2^{\cdot-}}]) = (k_\mathrm{d} + k_\mathrm{cat})[\mathrm{Ni} - \mathrm{CO_2^{\cdot-}}]$$

Rearranging the above equation yields,

$$[\mathrm{Ni}-\mathrm{CO}_2^{\cdot -}] = \frac{K[\mathrm{CO}_2][\mathrm{Ni}]_0}{1+K[\mathrm{CO}_2]}$$

Where $K = \frac{k_\mathrm{a}}{k_\mathrm{d}+k_\mathrm{cat}}$ is a measure of the CO_2 binding affinity of a catalyst.

Substituting [Ni-$CO_2^{\cdot -}$] in the rate equation,

$$v_\mathrm{CO} = \frac{k_\mathrm{cat}K[\mathrm{CO}_2][\mathrm{Ni}]_0}{1+K[\mathrm{CO}_2]}$$

The rate-determining step involves the second electron transfer step, and thus, the j_CO is dependent on the applied potential:

$$j_\mathrm{CO} = \frac{nFk^\mathrm{o}_\mathrm{cat}K[\mathrm{Ni}]_0P_{\mathrm{CO}_2}}{1+KP_{\mathrm{CO}_2}}\exp\left(\beta\eta F/RT\right)$$

where $k^\mathrm{o}_\mathrm{cat}$ is the standard rate constant for k_cat; β is the symmetry factor for the reaction, and the other symbols are as commonly known. The data in **Figure 4g** is fitted with the following equation:

$$j_\mathrm{CO} = \frac{\mathrm{a}P_{\mathrm{CO}_2}}{1+bP_{\mathrm{CO}_2}}$$

where a = $nFk_\mathrm{cat}K[\mathrm{Ni}]_0$; b $= K$.

(2) Reaction order

The reaction order with respect to CO_2 is determined by measuring the current response across varying CO_2 concentrations. The relationship between the partial current density of CO (j_{CO}) and CO_2 concentration can be categorized into two distinct regimes: a diffusion-limited region and a kinetically controlled region.

For the IsotropicNano catalyst, the reaction order in the diffusion-limited region is 1.09, while in the kinetically controlled region, it approaches 0. Notably, the diffusion-limited region for IsotropicNano (0–12% CO_2) is narrower compared to the other two samples (0–20% CO_2), suggesting that IsotropicNano exhibits reduced diffusion limitations.

In the kinetically controlled region, the reaction order remains higher (0.2–0.3) for DireactionalMeso and IsotropicMeso, indicating that the reaction still depends on reactant concentration due to insufficient mass transport.

Supplementary Table 8. Summary of reaction order results.

Condition	DirectionalMeso	IsotropicMeso	IsotropicNano
Diffusion-controlled regime	1.01	0.95	1.09
Kinetically controlled regime	0.26	0.17	0.09

(3) Activation energy

In electrocatalysis, activation energy (E_a) is the minimum energy needed for an electrochemical reaction to occur. At a constant potential, a lower activation energy signifies a more efficient reaction with reduced energy input, reflecting enhanced charge transfer properties.

$$k = Ae^{-E_a/RT}$$

Taking the natural logarithm:

$$\ln\ k = \ln\ A - \frac{E_a}{RT}$$

We varied the temperature from 273 to 333 K in an MEA-based CO_2 electrolyzer while maintaining a constant cell voltage of 2.7 V.

The partial current density of CO (j_{CO}) was measured and calculated. In electrochemistry, the reaction rate constant (k) is proportional to the current density (k ≈ j), allowing the equation to be rewritten as:

$$\ln\ j = \ln\ A - \frac{E_a}{RT}$$

After plotting the ln*j* vs. 1/T (Arrhenius plot), extract E_a from the plot,

$$E_a = -R \times (\text{ slope })$$

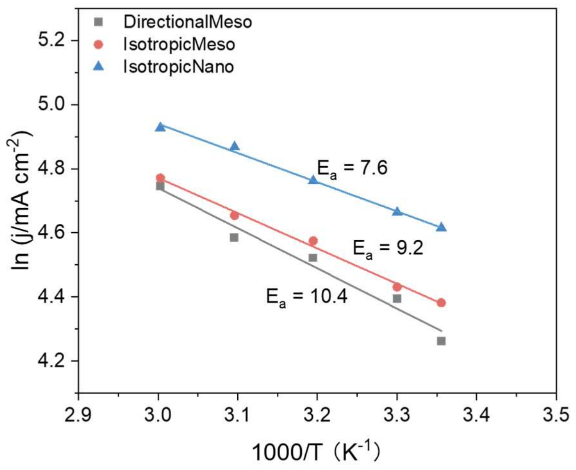

Supplementary Figure 44. Activation energy (E_a) for CO_2 to CO on three catalysts. The E_a value (slope) was calculated by Arrhenius plots of CO partial current density versus an inverse of the operating temperature.

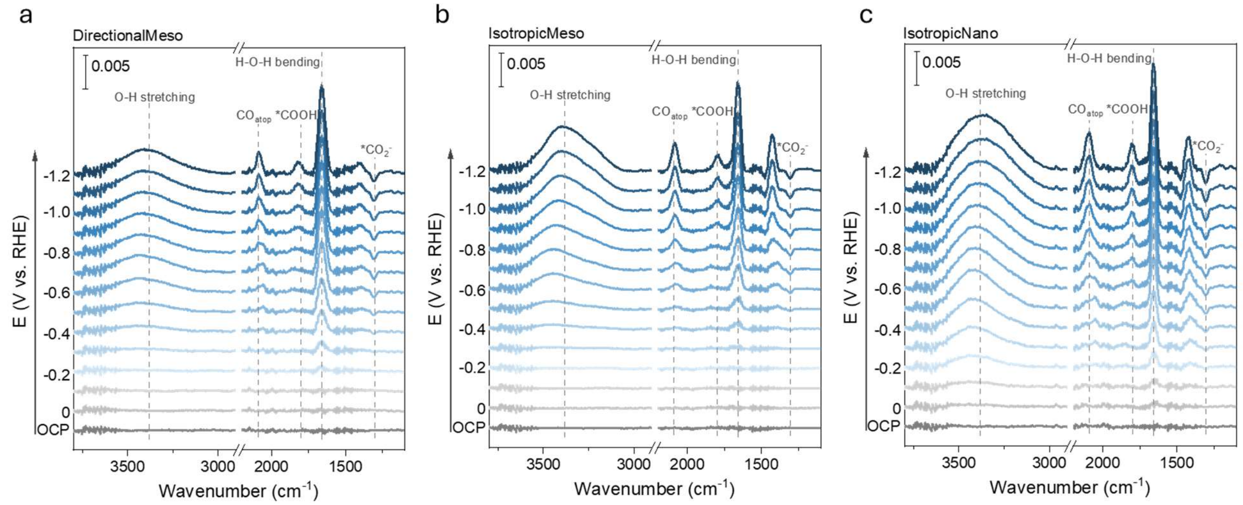

Supplementary Figure 45. In situ ATR-SEIRS spectra recorded at OCP and from 0 to −1.2V versus RHE on (a) DirectionalMeso, (b) IsotropicMeso, and (c) IsotropicNano.

Supplementary Table 9. Summary of deconvolution of O–H stretching peaks.

Catalyst	Potential (V vs. Ag/AgCl)	Free water (%)	2-HB H_2O (%)	4-HB H_2O (%)
DirectionalMeso	-1.8	9.1	48.2	42.7
	-1.7	9.0	47.8	43.3
	-1.6	9.0	48.5	42.5
	-1.5	9.0	50.4	40.5
	-1.4	9.0	53.0	38.1
	-1.3	9.0	54.9	36.2
	-1.2	9.4	55.6	34.9
	-1.1	10.6	57.2	32.3
	-1.0	12.0	58.1	29.9
IsotropicMeso	-1.8	2.7	49.0	48.3
	-1.7	3.2	51.6	45.2
	-1.6	3.3	53.1	43.6
	-1.5	2.7	52.3	45.0
	-1.4	1.7	57.8	40.5
	-1.3	1.0	64.1	34.9
	-1.2	0.9	62.7	36.4
	-1.1	0.6	63.2	36.2
	-1.0	0.4	62.1	37.5
IsotropicNano	-1.8	1.8	41.9	56.2
	-1.7	1.8	42.4	55.9
	-1.6	1.7	44.1	54.2
	-1.5	1.7	45.8	52.5
	-1.4	1.6	48.0	50.4
	-1.3	1.6	50.0	48.5
	-1.2	1.8	53.3	44.8
	-1.1	2.0	59.9	38.2
	-1.0	1.8	63.3	34.8

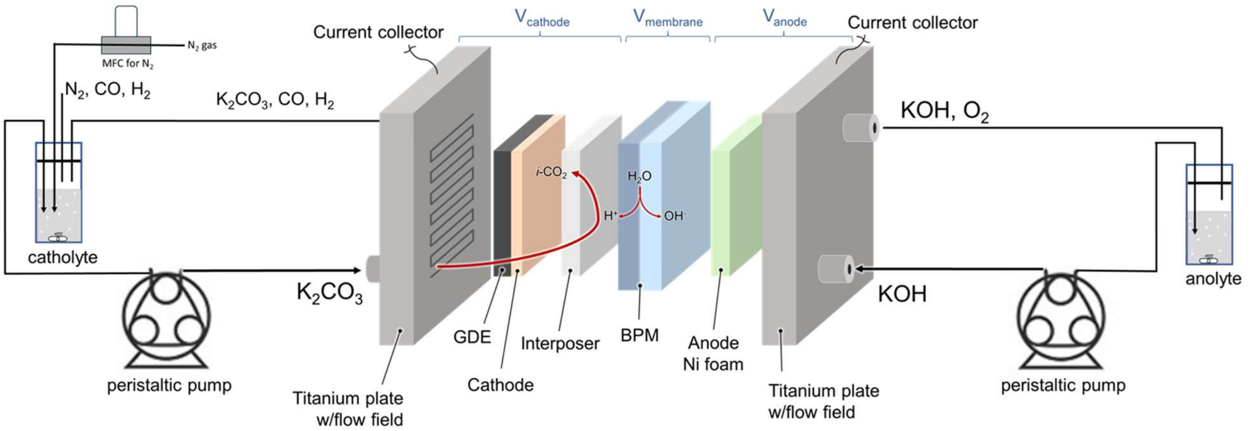

Supplementary Figure 46. Schematic illustration of the experimental setup for carbonate system in a MEA-based configuration.

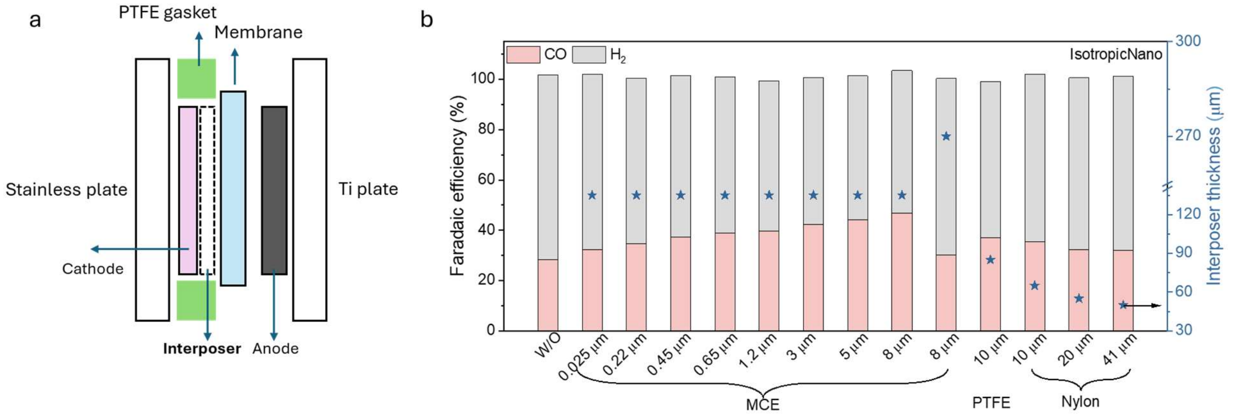

Supplementary Figure 47. Interposer layer optimization. (a) Schematic illustration of the MEA-based carbonate electrolysis cell configuration incorporating an interposer layer. (b) Faradaic efficiency to products (left Y-axis) as a function of interposer pore size (x-axis) and corresponding interposer thicknesses (right Y-axis) for different interposer types.

The interposer serves two key functions: regulating local pH and controlling *i*-CO_2 availability. As detailed in our previous work,[1] the pH profile and *i*-CO_2 concentration as a function of distance indicate that the spacing between the cation exchange layer (CEL) of the BPM and the electrocatalyst significantly influences species distribution in the reactive capture system.

Without an interposer (closely spaced CEL and catalyst), the locally low pH favors HER. Additionally, CO_3^{2-} and OH^- ions readily diffuse toward the CEL, neutralizing its acidic surface and thereby suppressing *i*-CO_2 generation.

By introducing a hydrophilic interposer layer (spacer) between the CEL and catalyst layer (CL), a pH gradient is established, creating optimal conditions:

- Low pH (<4) at the CEL to promote efficient *i*-CO_2 generation
- Higher pH and [CO_2] > 4 vol% at the CL to enhance CO_2RR performance

This strategic design effectively balances CO_2 availability and local reaction conditions, improving the overall efficiency of the system.

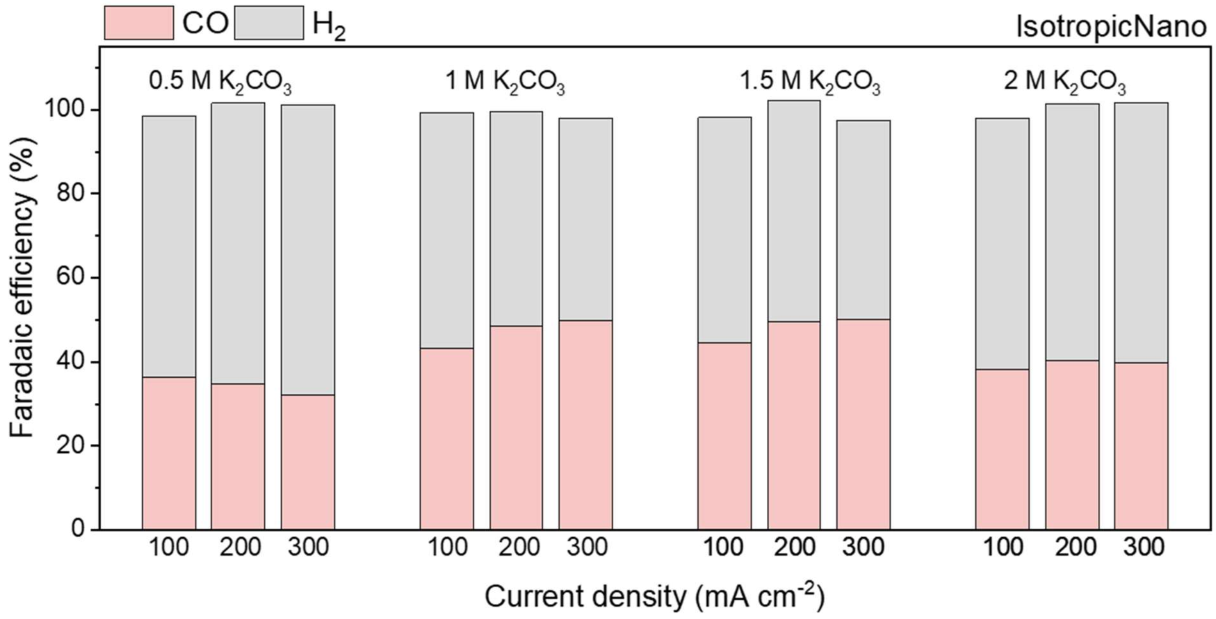

Supplementary Figure 48. Faradaic efficiency to products as a function of K_2CO_3 concentrations.

A K_2CO_3 concentration of 1–1.5 M maintains a high FE_{CO} (>45%). Lower K_2CO_3 concentrations reduce the likelihood of CO_3^{2-} reacting with H^+ on the BPM surface, leading to insufficient *i*-CO_2 generation and limiting *i*-CO_2 supply to the cathode.

$$CO_3^{2-} + 2H^+ \rightarrow CO_2 + H_2O$$

A higher K_2CO_3 concentration may increase *i*-CO_2 loss during its generation on the CEL surface and its transport to the cathode layer.

Side reaction at the CEL/catholyte interface:

$$CO_3^{2-}(aq) + H^+ \rightleftharpoons HCO_3^-(aq)$$

Side reaction during *i*-CO_2 transport:

$$CO_2(g) + CO_3^{2-}(aq) + H_2O(l) \rightleftharpoons 2HCO_3^-(aq)$$

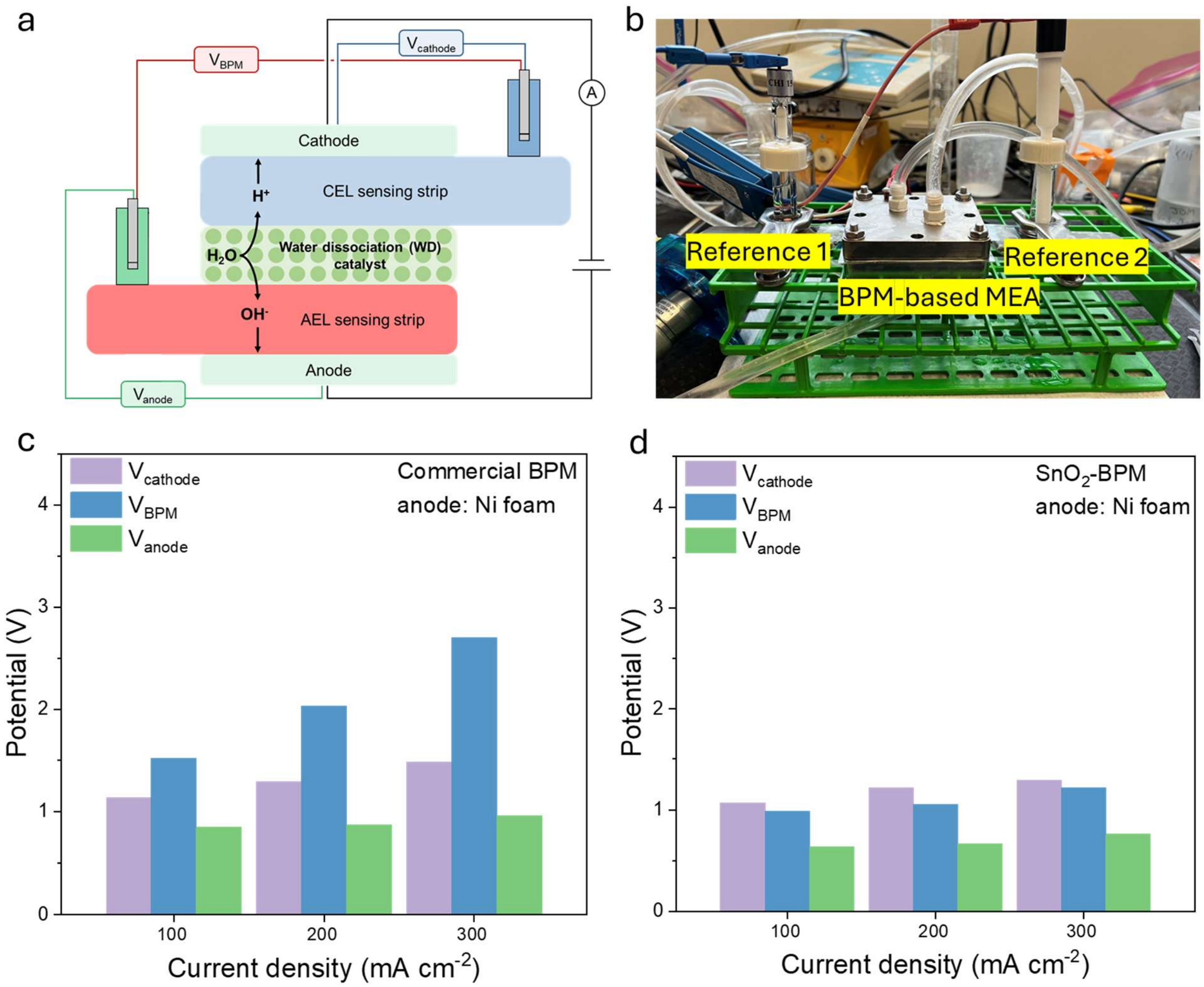

Supplementary Figure 49. Voltage breakdown measurements in MEA-based carbonate electrolyzer. (a) (a) A conceptual depiction illustrates the setup used for monitoring membrane potential in a BPM electrolyzer. The arrangement includes two reference electrodes and two sensing strips to track voltage distribution across the system. (b) Photograph of the experimental set-up. (c) Voltage breakdown characteristics of a commercial BPM-based electrolyzer and (d) a custom-designed BPM-based electrolyzer are observed under varying current densities. The total cell voltage is derived from the combined potential contributions of all components and is measured after achieving steady-state electrolysis conditions over a 5-minute period.

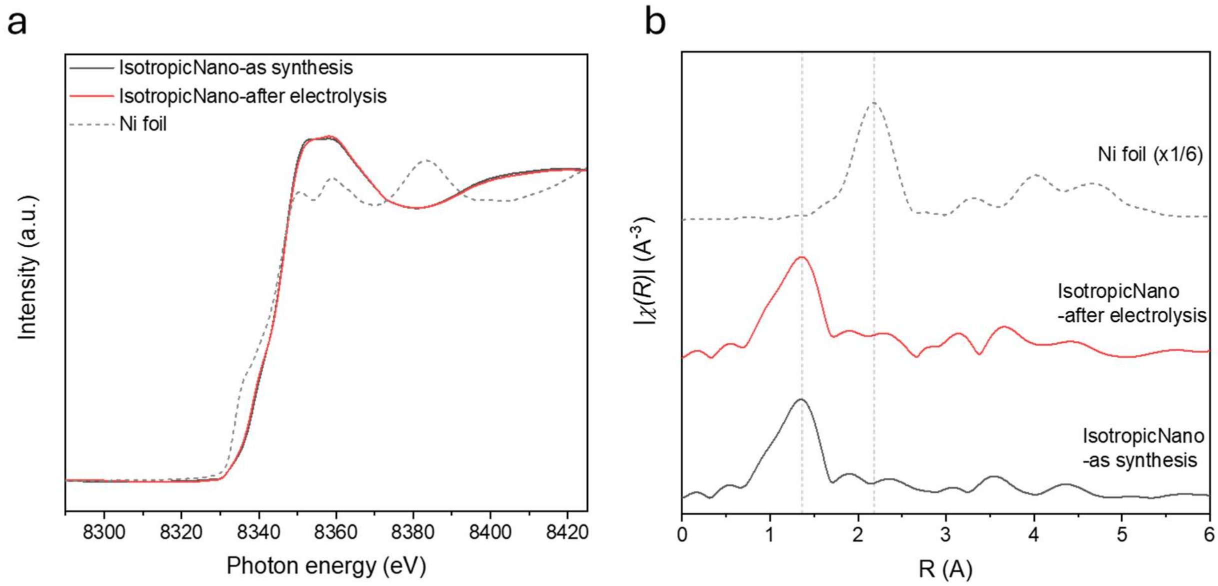

Supplementary Figure 50. (a) XANES spectra of IsotropicNano before and after carbonate electrolysis. (b) EXAFS Ni K-edge spectra indicating a Ni single-atom structure, demonstrating that this structure is preserved after the electrochemical reaction.

Supplementary Table 10. Comparison of electrochemical conversion of carbon captured liquids.

Electrolyzer feed solution	Membrane	Desired product	Cathode	Anode	Current density ($mA\ cm^{-2}$)	Cell voltage (V)	FE of product (%)	Stability (h)	Ref.
K_2CO_3	SnO_2-BPM	CO	IsotropicNano	NiFeP	300	3.3	50	50	This work
	BPM	CO	Ag NPs	Ni foam	300	4.3	12	145	[16]
	CEM	CO	Ag NPs	IrO_x/Ti	300	3.6	32	23	[17]
	BPM	C_2H_4	CuAg	Ni foam	200	4.4	10	24	[18]
	CEM	C_2H_4	Cu/CoPc-CNTs	IrO_x/Ti	300	4.2	34	24	[1]
$KHCO_3$	BPM	CO	Ag NPs	Ni foam	100	N/A	30	30	[19]
	BPM	CO	Ni-SAC	IrO_2-Ti mesh	300	4.5	20	5	[20]
	BPM	CO	Ag NPs	Ni foam	200	N/A	62	N/A	[21]
	BPM	CO	Ag-coated carbon cloth	Platinum mesh	100	3.5	37	N/A	[22]
	BPM	CO	Ni-SAC	Ni foam	300	3.7	50	18	[23]
	BPM	CH_4	Porous Cu	Ni foam	400	7.0	27	N/A	[24]
	BPM	$HCOO^-$	Bi/C	Ni foam	100	4.1	65	N/A	[25]
	BPM	$HCOO^-$	Hydrophilic Bi	Ni foam	300	4.0	85	30	[26]
	BPM	C_{2+}	CuSus/AgNaf	Ni foam	300	N/A	9	N/A	[27]
NH_4HCO_3	AEM	$HCOO^-$	ED-Bi	Ni foam	300	N/A	48	5	[28]
$KHCO_3$ + K_2CO_3	BPM	CO	Ag/C	Ni foam	50	3.5	13	N/A	[29]
Amine-CO_2	CEM	CO	Ag	Ni foam	100	N/A	19	10	[30]
	CEM	CO	Ni-N/C	TiO_2	100	N/A	30	N/A	[31]

Supplementary Table 11. Performance comparison and energy analysis of systems from air to syngas[a]: carbonate electrolysis, bicarbonate electrolysis, DAC-coupled with alkaline CO_2RR, neutral CO_2RR, and acidic CO_2RR.

System	Carbon utilization (%) [c]	CO concentration at the outlet (wt %)	Energy consumption (GJ/tonne of syngas)	References
**DAC-carbonate electrolysis**	**99**	**47**	**48**	**This work**
DAC-carbonate electrolysis	99	25	75	ACS Energy Letters, 2019, 4(6): 1427-1431.
DAC-bicarbonate eletrolysis[b]	40	46	60	Energy & Environmental Science 17.10 (2024): 3570-3579.
Acidic CO_2RR	84	13	62	Nature Sustainability (2024): 1-7.
Acidic CO_2RR	75	N/A	76	Nature Synthesis (2024): 1-12.
Acidic CO_2RR	14	N/A	85	Nature communications 12.1 (2021): 4943.
Neutral CO_2RR	40	70	77	Nature Chemical Engineering 1.3 (2024): 229-239.
Alkaline CO_2RR	15	5	158	Science 365.6451 (2019): 367-369.

a. Syngas: molar ratio of H_2: CO = 2:1
If FE_{CO} < 33%, additional CO was supplied from DAC-SOEC process. When an SOEC is assumed, the EE to CO is set to 80%. The analysis includes the needed CO_2 regeneration, circulation, and an air contactor. The resultant effective EE for CO is 25% in this case of CO-infill.
If FE_{CO} > 33%, additional H_2 from a water electrolyzer having EE for H_2 of 65% was assumed.

b. $CO_{2(g)}$ regeneration from carbonate post-capture liquid is powered using natural gas.
For bicarbonate electrolysis, additional pH downshifter energy is required to convert carbonate post capture liquid to bicarbonate. The energy consumption for pH downshifter is obtained from *Industrial & Engineering Chemistry Research* 61.34 (2022): 12668-12679.

c. Carbon utilization is defined as:

$$\text{Carbon utilization} = (1 - \frac{n_{CO2\,(g)}}{n^0_{CO2}}) \times 100\,\%$$

where n^0_{CO2} is the number of moles of supplied CO_2; $n_{CO2\,(g)}$ is the number of moles of gas CO_2 detected at the outlet of the catholyte; F is the Faraday constant (96,485 C mol^{-1}); Q (C) is the total charge passed through the electrolytic cell.

Supplementary Note 6. Energy consumption estimation

(1) Energy consumption for direct air capture

The energy consumption for direct air capture (DAC) is modeled on a Carbon Engineering process utilizing KOH as the CO_2 capture absorbent.[32] The air contactor requires approximately 0.3 GJ of energy per tonne of CO_2 captured. For a syngas mixture with a 2:1 molar ratio of H_2 to CO and a total mass of 1 tonne, the CO component is estimated to weigh 0.875 tonnes. Assuming a 99% efficiency in the conversion of CO_2 to CO, the energy consumption is determined as follows:

$$\left(\frac{0.875}{28} \times \frac{44}{0.99}\right) \times 0.3 = 0.42\ \text{GJ/tonneSyngas}$$

(2) Concentrated gas CO_2 regeneration, separation, and circulation

CO_2 from the air was captured using an alkali hydroxide solution and subsequently released at high purity through a high-temperature process (approximately 900 °C). The energy cost was estimated based on data from a prior study.[32] The energy consumption is 10 GJ per tonne of CO_2.

(3) Gas CO_2 separation and circulation

The energy requirements for CO_2 separation and circulation vary depending on the technology employed. When utilizing the Pressure Swing Adsorption (PSA) method, the energy consumption is approximately 1.1 GJ per tonne of CO_2.[33]

Sequential DAC capture and electrified conversion

(i) DAC coupled with a high-temperature solid oxide electrolysis cell (SOEC)

DAC is followed by feeding the purified CO_2 into a high-temperature solid oxide electrolysis cell (SOEC) for CO_2-to-CO conversion typically at 700−900 °C. The optimum energy efficiency for SOEC can attain 80%.[34-36] Unreacted CO_2 is separated from CO stream and circulated in this device to achieve 90% CO_2-to-CO conversion. Green H_2 is produced from water electrolysis with an EE of 65%, for matching the desirable H_2:CO ratio of 2:1.

(ii) DAC coupled with a reverse water-gas shift (RWGS) process

This approach uses RWGS process to convert high purity CO_2 (from DAC) and green H_2 (from water electrolyzer) to yield 2:1 syngas. We assume that the RWGS operates at 70% energy efficiency for CO_2-to-CO reaction.[37] Unreacted CO_2 is separated and circulated to achieve 90% CO_2-to-CO conversion.

Typically, this process costs 16 GJ/tonneCO for electrolysis and heat.[38] Green H_2 is produced from water electrolysis with an EE of 65%, for matching the desirable H_2:CO ratio of 2:1.

CO_2 from the air was captured using an alkali hydroxide solution and subsequently released at high purity through a high-temperature process (approximately 900 °C). The energy cost is 10 GJ per tonne of CO_2.

DAC coupled with electrochemical CO_2 reduction at ambient conditions

After DAC and the release of CO_2 gas from high-temperature processes, the concentrated pure CO_2 can be delivered to CO_2 electrolyzers under ambient conditions for further conversion. Depending on the electrolyte and reaction parameters, CO_2 electrolysis is typically conducted under basic, neutral, or acidic conditions. The CO_2 conversion, utilization, and energy consumption are summarized in **Table S11**.

Integrated approach reactive capture

The integrated approach avoids the energy consumption for CO_2 regeneration, separation, circulations. After DAC using an air contactor, the carbonate capture solution is directly feed into the electrolyzer for its further conversion. The output of the carbonate electrolyzer is complemented by H_2 production from a water electrolyzer (65% EE) to obtain a H_2/CO molar ratio of 2.

For KOH-based direct air capture (DAC) of CO_2 from air at low concentrations (~400 ppm), the process typically yields a K_2CO_3-rich solution for electrolysis. Integrating KOH with CO_2 down to K_2CO_3, pH ~12, is realistic; whereas contacting all the way down to $KHCO_3$ (pH ~8) is not.[39] Therefore, an additional step to lower the pH is necessary before proceeding with $KHCO_3$ electrolysis.

Bipolar membrane electrodialysis (BPMED) is the most commonly employed method for this pH adjustment. Studies indicate that the minimum energy consumption for BPMED is approximately 23 GJ per tonne of CO_2,[40] corresponding to 23/44*28 GJ per tonne of CO. This equates to about 12.8 GJ for producing one tonne of syngas, given a 2:1 H_2:CO molar ratio and considering syngas composition.

Supplementary Note 7. Comparison between carbonate and bicarbonate systems for reactive capture

We would like to point out the differences between two systems:

(1) CO_2 capture scenarios (thermodynamics):

- KOH-based direct air capture (DAC): Requires concentrated KOH as capture liquid due to low CO_2 levels (~400 ppm), producing a K_2CO_3-rich post capture solution (*Joule* 2.8 (2018): 1573-1594).[32]
- K_2CO_3-based flue gas capture: Can use K_2CO_3 as the absorbent since CO_2 concentrations are higher (4–15%),[41] resulting in a $KHCO_3$-rich post capture solution.

(2) Kinetics and practical considerations

For efficient DAC kinetics and cost-effective contactor design, capturing CO_2 with KOH down to K_2CO_3 (pH ~12) is feasible, whereas further conversion to $KHCO_3$ (pH ~8) poses significant challenges. The lower CO_2 concentration (~400 ppm) slows capture kinetics considerably as pH decreases from >14 (KOH) to ~12 (K_2CO_3).

In practical applications, transitioning K_2CO_3 to $KHCO_3$ demands substantially more contactors—up to 14 times higher than the KOH-to-K_2CO_3 process—to maintain the same CO_2 capture rate.[2] Given that air contactors contribute significantly to DAC capital costs, a $KHCO_3$-based approach remains impractical unless substantial cost reductions occur.

An alternative strategy involves integrating a pH adjustment system, such as bipolar membrane electrodialysis (BPMED), between the DAC post-capture liquid and the carbon conversion electrolyzer. However, this increases both energy demand and capital costs, making it less attractive. Consequently, enhancing product selectivity and energy efficiency in K_2CO_3-rich post-capture liquid-fed electrolyzer remains the more practical pathway.

(3) Differences in local environments for carbonate and bicarbonate electrolysis.

**Reactive capture system**	H^+ needed for one *i*-CO_2	bulk pH	local pH during electrolysis at the catalyst layer	*i*-CO_2 utilization (%)
Bicarbonate	1	8.5	9-11	40-60
Carbonate	2	~12	13-14	>99

In an electrolyzer, CO_3^{2-} or HCO_3^- ions must first convert into in situ CO_2 (g) and CO_2 (aq) to enable continuous reduction. In carbonate systems, *i*-CO_2 generation demands twice the H^+ amount, resulting in lower *i*-CO_2 evolution than in bicarbonate systems. The absence of buffering capability in carbonate

electrolyzers destabilizes local pH, leading to considerable *i*-CO_2 loss through acid-base neutralization. Therefore, catalyst development should focus on ensuring stability in alkaline conditions while optimizing *i*-CO_2 utilization.

The carbonate system demonstrates superior *i*-CO_2 utilization efficiency compared to bicarbonate systems, which suffer from losses due to spontaneous HCO_3^- decomposition and increased *i*-CO_2 supply and loss rates. Enhanced *i*-CO_2 utilization in carbonate systems facilitates the direct production of pure syngas, eliminating the need for additional separation steps and thereby reducing overall process costs.

Building on the above analysis, improving product selectivity in the electrolysis of post-captured carbonate solutions is crucial for efficient direct air capture-to-product conversion. Given the challenging conditions for *i*-CO_2 generation, transport, and utilization in the carbonate system, developing highly effective catalysts is essential to maximize performance.

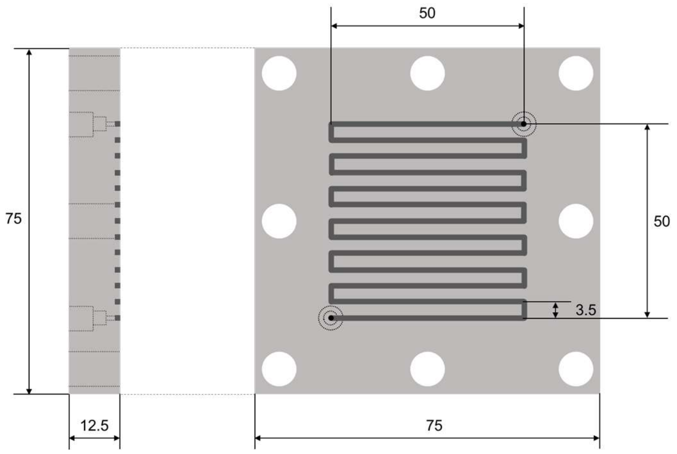

Supplementary Figure 51. Scaled flow plates. The channel depth is 1 mm, width is 1 mm, and the rid distance is 3.5 mm. The active area length is 50 mm, resulting in an electrode active area of 25 cm^2.

Reference

1 Lee, G. *et al.* CO2 electroreduction to multicarbon products from carbonate capture liquid. *Joule* **7**, 1277-1288 (2023).

2 Almajed, H. M. *et al.* Closing the Loop: Unexamined Performance Trade-Offs of Integrating Direct Air Capture with (Bi) carbonate Electrolysis. *ACS Energy Letters* **9**, 2472-2483 (2024).

3 Zhao, D. *et al.* Triblock copolymer syntheses of mesoporous silica with periodic 50 to 300 angstrom pores. *Science* **279**, 548-552 (1998).

4 Kleitz, F., Choi, S. H. & Ryoo, R. Cubic Ia 3 d large mesoporous silica: synthesis and replication to platinum nanowires, carbon nanorods and carbon nanotubes. *Chemical Communications*, 2136-2137 (2003).

5 Kresge, a. C., Leonowicz, M. E., Roth, W. J., Vartuli, J. & Beck, J. Ordered mesoporous molecular sieves synthesized by a liquid-crystal template mechanism. *Nature* **359**, 710-712 (1992).

6 Li, P. *et al.* Bifunctional electrocatalyst with CoN3 active sties dispersed on N-doped graphitic carbon nanosheets for ultrastable Zn-air batteries. *Applied Catalysis B: Environmental* **316**, 121674 (2022).

7 Yang, H. B. *et al.* Atomically dispersed Ni (i) as the active site for electrochemical CO2 reduction. *Nature energy* **3**, 140-147 (2018).

8 Zheng, T. *et al.* Large-scale and highly selective CO2 electrocatalytic reduction on nickel single-atom catalyst. *Joule* **3**, 265-278 (2019).

9 Teixeira, J. Small-angle scattering by fractal systems. *Applied Crystallography* **21**, 781-785 (1988).

10 Li, T., Senesi, A. J. & Lee, B. Small angle X-ray scattering for nanoparticle research. *Chemical reviews* **116**, 11128-11180 (2016).

11 Ren, Y. & Zuo, X. Synchrotron X‐ray and neutron diffraction, total scattering, and small‐angle scattering techniques for rechargeable battery research. *Small Methods* **2**, 1800064 (2018).

12 Reichhardt, N. *et al.* Removal of Intrawall pores in SBA-15 by selective modification. *Chemistry of Materials* **23**, 3400-3403 (2011).

13 Brückner, S. *et al.* Design and diagnosis of high-performance CO2-to-CO electrolyzer cells. *Nature Chemical Engineering* **1**, 229-239 (2024).

14 Johnson, K. A. & Goody, R. S. The original Michaelis constant: translation of the 1913 Michaelis–Menten paper. *Biochemistry* **50**, 8264-8269 (2011).

15 Seong, H. *et al.* Atomically precise gold nanoclusters as model catalysts for identifying active sites for electroreduction of CO2. *Angewandte Chemie* **133**, 14684-14691 (2021).

16 Li, Y. C. *et al.* CO2 electroreduction from carbonate electrolyte. *ACS Energy Letters* **4**, 1427-1431 (2019).

17 Xiao, Y. C. *et al.* Direct carbonate electrolysis into pure syngas. *EES Catalysis* **1**, 54-61 (2023).

18 Song, H. *et al.* Ethylene production from carbonate using a bipolar membrane electrolysis system. *ACS Applied Energy Materials* **7**, 1224-1233 (2024).

19 Zhang, Z. *et al.* Porous metal electrodes enable efficient electrolysis of carbon capture solutions. *Energy & Environmental Science* **15**, 705-713 (2022).

20 He, D., Ma, X., Zhou, H., Zhang, Y. & Wu, Y. Continuous conversion of flue gas into syngas by a bipolar membrane-integrated single-cell cyclic system. *Joule* (2025).

21 Lees, E. W. *et al.* Electrodes designed for converting bicarbonate into CO. *ACS Energy Letters* **5**, 2165-2173 (2020).

22 Li, T. *et al.* Electrolytic conversion of bicarbonate into CO in a flow cell. *Joule* **3**, 1487-1497 (2019).

23 Song, H. *et al.* Integrated carbon capture and CO production from bicarbonates through bipolar membrane electrolysis. *Energy & Environmental Science* **17**, 3570-3579 (2024).
24 Lees, E. W. *et al.* Electrolytic methane production from reactive carbon solutions. *ACS Energy Letters* **7**, 1712-1718 (2022).
25 Li, T., Lees, E. W., Zhang, Z. & Berlinguette, C. P. Conversion of bicarbonate to formate in an electrochemical flow reactor. *ACS Energy Letters* **5**, 2624-2630 (2020).
26 Nomoto, K., Okazaki, T., Beppu, K., Shishido, T. & Amano, F. Highly selective formate formation via bicarbonate conversions. *EES Catalysis* **2**, 1277-1284 (2024).
27 Lee, J., Liu, H. & Li, W. Bicarbonate Electroreduction to Multicarbon Products Enabled by Cu/Ag Bilayer Electrodes and Tailored Microenviroments. *ChemSusChem* **15**, e202201329 (2022).
28 Liu, H., Chen, Y., Lee, J., Gu, S. & Li, W. Ammonia-mediated CO2 capture and direct electroreduction to formate. *ACS Energy Letters* **7**, 4483-4489 (2022).
29 Gutierrez-Sanchez, O. *et al.* Electrochemical conversion of CO2 from direct air capture solutions. *Energy & Fuels* **36**, 13115-13123 (2022).
30 Lee, G. *et al.* Electrochemical upgrade of CO2 from amine capture solution. *Nature Energy* **6**, 46-53 (2021).
31 Kim, J. H. *et al.* The insensitive cation effect on a single atom Ni catalyst allows selective electrochemical conversion of captured CO 2 in universal media. *Energy & Environmental Science* **15**, 4301-4312 (2022).
32 Keith, D. W., Holmes, G., Angelo, D. S. & Heidel, K. A process for capturing CO2 from the atmosphere. *Joule* **2**, 1573-1594 (2018).
33 Ho, M. T., Allinson, G. W. & Wiley, D. E. Reducing the cost of CO2 capture from flue gases using pressure swing adsorption. *Industrial & Engineering Chemistry Research* **47**, 4883-4890 (2008).
34 Zheng, Y. *et al.* A review of high temperature co-electrolysis of H 2 O and CO 2 to produce sustainable fuels using solid oxide electrolysis cells (SOECs): advanced materials and technology. *Chemical Society Reviews* **46**, 1427-1463 (2017).
35 Belsa, B., Xia, L. & García de Arquer, F. P. CO2 Electrolysis Technologies: Bridging the Gap toward Scale-up and Commercialization. *ACS Energy Letters* **9**, 4293-4305 (2024).
36 Hauch, A. *et al.* Recent advances in solid oxide cell technology for electrolysis. *Science* **370**, eaba6118 (2020).
37 Theofanidis, S.-A., Stergiou, K., Delikonstantis, E. & Stefanidis, G. D. On the Electrification of CO2-Based Methanol Synthesis via a Reverse Water–Gas Shift: A Comparative Techno-Economic Assessment of Thermo-Catalytic and Plasma-Assisted Routes. *Industrial & Engineering Chemistry Research* **63**, 12035-12052 (2024).
38 Ozden, A. *et al.* Cascade CO2 electroreduction enables efficient carbonate-free production of ethylene. *Joule* **5**, 706-719 (2021).
39 Almajed, H. M. *et al.* Closing the Loop: Unexamined Performance Trade-Offs of Integrating Direct Air Capture with (Bi)carbonate Electrolysis. *ACS Energy Lett* **9**, 2472-2483 (2024). https://doi.org:10.1021/acsenergylett.4c00807
40 Sabatino, F., Gazzani, M., Gallucci, F. & van Sint Annaland, M. Modeling, optimization, and techno-economic analysis of bipolar membrane electrodialysis for direct air capture processes. *Industrial & Engineering Chemistry Research* **61**, 12668-12679 (2022).
41 Kim, Y. *et al.* Integrated CO2 capture and conversion to form syngas. *Joule* **8**, 3106-3125 (2024).
